# Machine Learning Prediction for Fe(II) Spin-Crossover Complex in the Same Spin State Using Geometrical and Topological Descriptors

**DOI:** 10.1021/acs.jcim.6c00219

**Published:** 2026-04-16

**Authors:** Natsumi Okawa, Tomoyuki Miyao

**Affiliations:** 1 Graduate School of Science and Technology, 12708 Nara Institute of Science and Technology, Ikoma, Nara 630-0192, Japan; 2 Data Science Center, 12708 Nara Institute of Science and Technology, 8916-5 Takayama-cho, Ikoma, Nara 630-0192, Japan

## Abstract

Spin-crossover (SCO) complexes are molecular materials capable of reversibly switching between high-spin (HS) and low-spin (LS) states in response to external stimuli. Predicting SCO from crystallographic data allows for the efficient design of new SCO complexes. However, no extensive and diverse data sets of complexes with annotated SCO characteristics were available. In the present study, we manually assembled a data set of 500 Fe­(II)–N_6_ coordination complexes with explicit spin states and SCO potential, termed FeN_6_–SSD. Using this data set, we built machine learning models to distinguish between SCO-undergoing and non-SCO complexes crystallized in the same spin state (either HS or LS). The classification results showed that the key factors for predicting SCO activity differed between the two spin states: in the HS regime, local geometric distortions, such as Fe–N bond elongation and octahedral deformation, were important, whereas in the LS regime, ligand-derived chemical and steric factors were important. Overall, the many-body tensor representation as a descriptor set achieved high prediction accuracy in both spin states. The influence of environmental factors, such as solvents and counterions, on SCO classification was inconsistent across representations.

## Graphical abstract

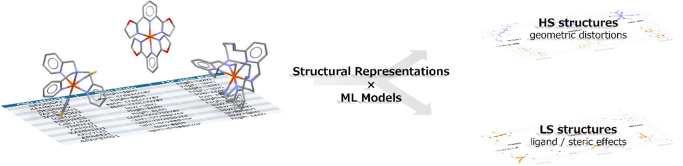

## 1 Introduction

Spin-crossover (SCO) complexes^1–5^ are molecular materials capable of reversibly switching^4^ between high-spin (HS) and low-spin (LS) states in response to external stimuli, including heat,^1–5^ pressure,^2–4^ and light.^2–4,6^ Since the transition between HS and LS states involves changes in molecular structure and visible absorption properties (perceived as color changes), SCO complexes are expected to be utilized for memory devices and sensing materials.^4,5,7,8^ Among various SCO complexes, Fe­(II) complexes,^9–12^ which exhibit paramagnetism in the HS state and diamagnetism in the LS state, have been intensively investigated, supported by the number of registered SCO complexes in the Cambridge Structural Database (CSD).^13^ For Fe­(II) complexes, the HS state corresponds to the quintet state (S = 2), whereas the LS state corresponds to the singlet state (S = 0).

Whether SCO characteristics appear depends not only on molecular-level factors such as ligand-field strength and the energy difference between HS and LS states,^12,14^ but also on bulk-level factors such as steric arrangement and intermolecular interactions.^15^ For example, complexes in the [Fe­(1-bpp)­2]^2+^ series display diverse spin-crossover behavior depending on crystal packing and structural distortions. In this series, some salts enable abrupt or cooperative spin transitions, while others stabilize complexes in the HS state over a range of temperatures, despite having similar coordination environments.

The development of SCO complexes relies heavily on an experimental trial-and-error approach, where candidate complexes are synthesized individually and tested for their spin states. Although experimental validation is indispensable for novel material design, this conventional approach is labor-intensive and time-consuming, and many synthesized complexes fail to exhibit SCO behavior. Therefore, efficient computational approaches are required to predict whether a complex exhibits SCO behavior prior to material synthesis.

Several computational approaches have been developed to predict the SCO behavior of complexes. To predict SCO complexes, computational chemistry and data-driven approaches have been investigated. The former aims to compute the thermodynamic energy difference between the HS and LS states, assuming the two states are in thermal equilibrium. This allows for the use of electronic energies, the Gibbs free energy, and structural parameters derived from optimized geometries—such as Fe–N bond lengths—to assess whether a complex exhibits SCO behavior. However, these methods often exhibit limited predictive accuracy and poor generalizability due to challenges in modeling open-shell systems and to the simplifications involved in ignoring bulk effects, such as crystal packing and counterion interaction.^12,14,16–21^ On the other hand, data-driven approaches leverage crystallographic data, mostly extracted from the CSD, to analyze the relationship between spin states and geometric features (descriptors) such as coordination bond lengths, angles, and torsions.^10,12,22^ Marchi et al. extracted geometric parameters from the metal–ligand complexes of Fe­(II) and tridentate bis-pyrazolylpyridyl (bpp) derivatives from the CSD.^12^ Using a chemometric multivariate analysis tool (e.g., principal component analysis), they examined the structural distribution for HS state complexes from two groups: potentially undergoing SCO and blocked in the HS state. A clear separation between these groups was observed when the analysis was restricted to solvent-free crystal structures, highlighting the importance of bulk environmental effects. Although they further developed a machine learning (ML) model using geometric parameters to predict the transition temperature, they did not report test-set prediction accuracy, limiting a fair assessment of the model’s performance. Vennelakanti et al. utilized topological autocorrelation descriptors, termed revised autocorrelations (RACs),^23^ to predict the transition temperatures of SCO complexes. The ML model was trained on SCO complexes from a manually curated Fe­(II) complex data set: SCO-95.^19^ Although RACs are topological descriptors (that do not use atomic coordinates), the descriptor set showed some advantages in predicting transition temperatures compared with computational chemistry approaches, with a Pearson correlation coefficient of 0.36 between predicted and observed temperatures for a test data set.

Although these studies indicate that data-driven methods are promising for objectively analyzing SCO complexes, several research questions remain, including the generalizability of data-driven models, the importance of geometrical information, the effect of bulk information (solvents and counteranions) on the prediction accuracy of the models, and how to classify complexes capable of undergoing SCO from non-SCO complexes in the same spin state (HS or LS state).

Most studies have focused on a specific series of SCO complexes to understand the structural characteristics essential to SCO complexes,^12,24^ with many predictive approaches being based on structurally constrained classes of compounds, such as Marchi et al.’s analysis of Fe­(II)–bpp derivatives and Phan et al.’s model using homoleptic Fe­(II) tris-diimine complexes. However, SCO behavior has been reported for a wide range of coordination geometries and molecular structures. It is crucial to understand the generalizability (scope and limitations) of ML models (point 1). Previous studies used topological and geometrical descriptors exclusively; no comparison between them was provided. If the prediction accuracies of models using these two types of descriptors are comparable, we could use a topological-descriptor-based model for designing SCO complexes (point 2). Although several studies highlight the importance of bulk information for SCO, ML models utilizing this information have not been developed (the closest research is the analysis by Marchi et al.^12^) (point 3). Finally, since geometrical parameter values differ between spin states, it is not difficult to differentiate spin states using these parameters, including the metal–ligand distance. An open question is whether there are geometrical and topological parameters that distinguish complexes crystallized in the same spin state but differing in SCO activity.

Herein, to provide answers or hints to the questions above, we develop ML models to discriminate SCO-undergoing and non-SCO complexes crystallized in the same spin state (HS or LS). We manually collected and curated CSD-registered Fe­(II) complexes to prepare a high-quality, structurally diverse set of SCO and non-SCO complexes, including associated bulk information. Specifically, we focus on those with N_6_ coordination environments, covering a wide range of ligand denticities from monodentate to hexadentate. The curated data set comprised 235 unique Fe­(II) mononuclear coordination complexes with N_6_ coordination environments, including both SCO and non-SCO species. These are selected from a curated subset of 500 CSD crystal entries — obtained after screening a larger number of Fe­(II)–N_6_ structures — to ensure structural diversity and accurate spin-state assignments. This collection is hereafter referred to as the Fe­(II)–N_6_ Spin-State Data set (FeN_6_–SSD). Using this data set, we construct ML models based on topological and geometrical descriptors, with and without incorporating bulk environmental factors. We hope that the insights and data set provided in this study will lead to rational design using data-driven approaches for future SCO complex development.

## 2 Materials and Methods

### 2.1 FeN_6_–SSD Creation

Understanding the behavior of ML models for predicting SCO complexes requires a data set of crystal structures where spin states are explicitly linked to the structures. While the CSD^13^ provides crystallographic data, it does not include explicit annotations of spin states. Therefore, we manually linked the crystallographic data to their spin state reported in the literature. Fe­(II) mononuclear octahedral complexes coordinated by N_6_ were focused on to enable the construction of a looser, more diverse data set in terms of coordination environments compared with previous studies.^10,12,22^ Our data set, FeN_6_–SSD, was constructed as follows.

#### 2.1.1 Ligand Data Collection and Crystal-Structure Identification

Starting from comprehensive review articles on SCO complexes,^9,10^ we generated a list of ligand structures in the SMILES (Simplified Molecular Input Line Entry System) format, which is provided in Table S1 of the Supporting Information. Ligands with complex structures that are difficult to express in SMILES (e.g., ferrocene) were excluded. Ultimately, 575 unique SMILES were compiled into a query set.

Using the CSD Python API^25^ (v5.41, 2020.2.0, updated through August 2020, CCDC, UK), we retrieved crystallographic data in the CIF and Mol2 file formats for the SMILES queries. When multiple entries were identified for the same complex, the entry satisfying the following filtering criteria was prioritized: the original publication described whether or not SCO behavior was observed explicitly, the crystal structure was measured at low temperature (typically below 200 K) and/or at higher temperatures (typically around room temperature or near the reported SCO transition temperature), and the structures cited in the review articles.^9,10^ For complexes that could not be retrieved via SMILES-based queries, we manually searched them through ConQuest.^26^ In total, 644 crystal structures were identified.

#### 2.1.2 Assignment of Spin State

The relevant literature for each crystal structure was manually scrutinized to determine the spin state. Spin-state assignments were primarily based on experimental evidence reported in the literature, including temperature-dependent magnetic susceptibility measurements, Mössbauer spectroscopy, or EPR spectroscopy. The assigned spin states reflect the behavior observed within the measurement temperature ranges reported in the literature. The measurement temperature of each CSD structure was compared with the reported spin-state behavior to determine whether the structure corresponds to the HS or LS state. For spin-state identification, only experimental measurements that allow verification of spin-state behavior across multiple temperatures were considered. In most cases, temperature-dependent magnetic susceptibility measurements or Mössbauer spectroscopy were used to confirm the spin states. In one exceptional case where such measurements were unavailable, temperature-dependent EPR spectroscopy was used to determine the spin-state behavior. Spin states were assigned as follows:

1. LS state: A structure was classified as low-spin if it remained in the LS state across the reported measurement temperature range based on the available experimental evidence, and if the Fe–N average bond length in the CSD-registered structure (typically around 1.95–2.00 Å) was consistent with the LS state.
2. HS state: A structure was classified as high-spin if it remained in the HS state throughout the reported measurement temperature range based on the same types of experimental data, and if the Fe–N average bond length in the CSD-registered structure (typically around 2.15–2.20 Å) was consistent with the HS state.
3. SCO state: A structure was labeled as SCO if it exhibited LS behavior at low temperature and HS behavior at high temperature based on the same types of experimental evidence. Among such structures, those with Fe–N average bond lengths around 1.95–2.00 Å were treated as LS structures, and those with bond lengths around 2.15–2.20 Å were treated as HS structures.

Structures for which the spin-state information at either temperature was inaccessible were excluded, and the full list of excluded entries, along with the exclusion criteria applied, is provided in Table S3. Additionally, structures were excluded when the reported spin states were known to change due to solvent loss, but no postsolvent-loss crystal structures were available, or when they exhibited severely disordered bonding environments. As a result, a curated data set of 500 crystal structures with explicitly assigned spin states was obtained, as summarized in Table S2 of the Supporting Information.

#### 2.1.3 Extraction of Molecular Structures from a Complex Crystallized Structure

Original complex data (the CIF or Mol2 file formats) contained various components in the unit cell, like coordination complexes, counterions and crystallization solvents. Based on our objectives, which include understanding crystallization environment effects and ligands involved in SCO behavior, each component in a complex data file is extracted and organized accordingly. We noticed that none of the single chemoinformatics toolkits (e.g., RDKit and OpenEye) successfully processed the whole data set. Rather, a combination of toolkits achieved this goal. The detailed description of the curation process is provided in Section S1 of the Supporting Information. By applying the curation process to the complex data, Fe­(II)–N_6_ mononuclear complexes, counterions, and crystallization solvents were separated.

Each coordination complex was further dissected into the central metal atom (Fe) and its coordinated ligands. These ligands were extracted based on their connectivity to the center Fe and saved as individual Mol2 files. For each ligand, a manual inspection was conducted to determine its denticity (coordination number), which was assigned based on bonding patterns and the number of donor groups. The other components of the crystal complex data—specifically the counterions and crystallization solvents—were identified based on their formal charge and were labeled with the ion_ or solv_ prefix in a file name. In the cases where automatic detection was hindered by disorder or ambiguous bonding, manual corrections were conducted.

To summarize the extracted structural components, we compiled component types and counts—complexes, ligands, ions, and solvents—from the curated extraction results. For ions and solvents, the numbers of occurrences (count data) were taken directly from the chemical formulas reported in the CSD, and a binary presence/absence indicator was also generated. The binary indicators were used as environmental descriptors in the ML models in subsequent analyses. Noninteger solvent or counterion counts (e.g., 0.5 or 1.5) originate from crystallographic features reflected in the curated CSD formulas and were therefore retained as reported.

The resulting FeN_6_–SSD data set consists of 500 Fe­(II)–N_6_ mononuclear complexes with explicitly assigned spin states. It includes metadata such as CSD entry identifiers, spin-state assignments, and reference information (see Table S2). The data set is distributed under the Creative Commons Attribution 4.0 International (CC BY 4.0) license (https://creativecommons.org/licenses/by/4.0/); the original crystallographic data remain the property of the CCDC and FIZ Karlsruhe under their respective Terms of Use.

### 2.2 Structural Representations

To address the research questions—understanding the importance of geometrical information and the effect of bulk information (solvents and counterions) on classifying the SCO complexes from non-SCO ones in the same spin state—several representations have been tested. The extended connectivity fingerprint with a radius of 2 (ECFP4)^28^ and revised autocorrelations^23^ (RAC and RAC (no stereo)) were used as topological descriptors, relying primarily on molecular graph connectivity. RAC includes stereochemical distinctions such as axial and equatorial ligands, for which the axial/equatorial assignment was determined mechanically from the 3D geometry provided in each xyz structure, following the implementation in molSimplify.^33,34^ As a result, stereo-RAC values can exhibit slight variations across multiple xyz structures corresponding to the same CCDC entry. We therefore treat RAC as a topology-based descriptor in feature definition, while noting that stereo auxiliaries may show practical geometry dependence in rare cases (see Section 2.2.2 for details). Fully geometric descriptors include the OctaDist descriptors^29^ and the many-body tensor representation (MBTR^30^), both of which explicitly encode 3D structural information.

#### 2.2.1 ECFP4

ECFP^28^ is an atom environment fingerprint with a predefined radius. It can be a de facto standard for any chemoinformatics analysis for drug discovery^31^ and organic chemistry. In the calculation of ECFP, the atom feature assigned to each atom is iteratively updated by merging neighboring atom information, forming a unique feature representing its atom environment. Each atom feature corresponds to a hash value, which is subsequently placed on a bit in a vector by the modulo operation, forming a fixed-length bit vector. In this study, a radius of 2 was used (ECFP4), and the bit length was not predefined, the number of bits was determined as the number of unique hash values for avoiding bit collisions.

In this study, ECFP4 and a variant focusing only on the connection between the metal center and donor atoms were used to highlight the importance of metal–ligand interactions in coordination complexes.

#### 2.2.2 RAC

RAC extends conventional autocorrelation-based descriptors^32^ to better suit metal complexes by explicitly defining three types of origins: metal-centered, ligand-centered, and all-pairs. For each category, atomic correlations at each bond-distance level (i.e., topological depth) are accumulated to form a descriptor. While RAC is fundamentally based on graph topology and thus categorized as a topological descriptor, it also incorporates stereochemical distinctions such as axial/equatorial ligand positions. Here, the axial/equatorial assignment was derived from the xyz geometry according to the molSimplify implementation; consequently, stereo-RAC values can differ slightly across multiple xyz structures corresponding to the same CCDC entry. This implementation-dependent behavior was rare in the present data set and does not affect the main conclusions of this study.

In this study, we calculated the descriptor set commonly referred to as RAC-155 in the literature,^23^ using molSimplify.^33,34^ Atomic correlations were accumulated as features across topological depths (i.e., bond distances) 0–3, based on three perspectives (origins): metal-centered, ligand-centered, and all-atom pairs. While RAC allows the use of interatomic distance weighting, we did not include distance-based terms in our implementation. However, stereochemical auxiliaries such as axial/equatorial ligand distinctions were retained. The HF exchange term was excluded due to the absence of quantum chemical calculations, and the oxidation state was also omitted as all complexes were in a fixed +2 state, resulting in a final set of 153 descriptors. For simplicity, we denote this set as RAC.

For comparison, we also constructed a simplified version, referred to as RAC (no stereo), by removing stereochemical auxiliaries such as axial/equatorial distinctions, relying solely on 2D topological graph information. This version served as a control to isolate the contribution of stereochemical features within the RAC framework.

#### 2.2.3 OctaDist

OctaDist (Octahedral Distortion calculator) is a program for computing octahedral distortion parameters. In this study, we refer to the set of five geometric parameters obtained from OctaDist as OctaDist descriptors. These parameters include the average metal–ligand bond length, bond length distortion, angle distortion, torsional distortion, and tilting distortion parameter, represented by Greek characters as follows: the sigma, theta, zeta, and delta parameters, in addition to the mean metal–ligand bond length (Mean M–L BL). These parameters quantify the degree of geometric distortion in six-coordinate octahedral complexes and have been shown to effectively capture structural changes associated with spin-state transitions.^10,35,36^ The sigma parameter is the sum of the absolute differences between the cis angles in the octahedral geometry and the ideal 90°, quantifying an angular distortion of the octahedron. The theta parameter represents a cumulative torsional (twisting) distortion among the ligand sets by measuring how much the dihedral angles deviate from their ideal values. The zeta parameter represents a tilting distortion of the ligand arrangement and serves as a complementary indicator of overall octahedral deformation. The delta parameter represents a dispersion of the metal–ligand bond lengths relative to their mean value. The mean metal–ligand bond length is the average distance between the metal center and its coordinating ligand atoms, which provides insights into the overall bonding environment.^37^ In this study, OctaDist descriptors were calculated using OctaDist,^29^ with input structures converted from Mol2 to xyz formats.

#### 2.2.4 MBTR

MBTR encodes the 3D structure of a molecule as a continuous high-dimensional vector. It is characterized by a tuple of elements and the function applied to the tuple. In this study, up to three atomic species combinations were considered, and the vectors for these categories are concatenated to form MBTR. Specifically, *k*_1_ (unary term): frequency of atomic species, *k*_2_ (binary term): inverse distances between atomic pairs, and *k*_3_ (ternary term): the distribution of angles formed by atomic triplets were used, based on those adopted in a previous study.^38^ We generated the MBTR descriptor for both entire molecules and only Fe-centered local environments using the DScribe library.^39,40^

#### 2.2.5 Descriptor Variants Focusing on the Center Metal

We hypothesized that the atomic environment surrounding the center metal Fe­(II) is the most important for SCO prediction. Thus, we tested descriptor variants in which only the features associated with Fe­(II) were extracted, denoted by a *-Fe* suffix. In addition, we extended the descriptors by incorporating environmental information using a binary one-hot representation of the components, denoted by the *+env* suffix. As a result, the following descriptors were individually employed for model construction: MBTR, MBTR-Fe, MBTR + env, MBTR-Fe + env, ECFP4, ECFP4-Fe, ECFP4 + env, ECFP4-Fe + env, RAC, RAC + env, RAC (no stereo), RAC (no stereo) + env, OctaDist and OctaDist + env.

As a supplementary analysis, we also examined crystal-packing descriptors derived directly from the CIF files, including a periodic MBTR representation of the full crystal structure and CSD API-based crystal descriptors such as density, packing coefficient, void fraction, and intermolecular contact features. These descriptors were used to construct additional RF models; however, they did not provide a clear improvement in predictive performance. The detailed protocol and results are provided in the Supporting Information.

### 2.3 ML Modeling Methods

The structural representations (descriptors) introduced in Section 2.2 serve as the inputs of ML models. As a modeling method in this study, we used random forests (RF),^41^ a tree-based nonlinear ML method, due to its strong predictive performance and ease of model interpretation through well-designed interfaces. RF models classify SCO-undergoing and non-SCO complexes in the same spin state.

### 2.4 Preprocessing of Data Sets

Before building ML models, descriptor tables were converted into numeric feature matrices indexed by CCDC entry IDs. The intersection of sample IDs across all descriptor sets and the set of nonmissing labels were identified to construct a common data set. This common data set was then stratified into five folds, and the resulting folds were shared across all descriptor sets within the same spin state to conduct stratified cross-validation (CV) experiments.

Descriptor filtering procedures varied based on the analysis purposes and are described in Sections 2.5 and 2.6.

### 2.5 Model Evaluation Procedure

The prediction accuracy of ML models with various representations was evaluated using the Matthews correlation coefficient (MCC)^42,43^ and the F1 score, both of which are robust to data imbalance. For each combination of spin state (LS or HS) and descriptor set, a 5-fold nested CV^44^ was repeated three times. Metric values for these 15 test data sets were averaged, along with their standard deviations, to statistically evaluate differences between representations. For models using the full descriptor sets, descriptor filtering—including removal of descriptors with nonfinite values, variance filtering, and correlation-based pruning—was performed using only the training data within each outer fold to avoid information leakage. The fitted preprocessing transformations were subsequently applied to the corresponding test fold. Hyperparameters (n_estimators and max_features) in the scikit-learn-based RF models^45^ were optimized using inner cross-validation on the training data, and the optimized models were evaluated on the corresponding outer test folds to derive prediction accuracy.

In addition to using all descriptors within each descriptor category, we also evaluated the effect of focusing on important descriptors. Specifically, for each descriptor set and spin-state combination, the five most important features were identified based on feature importance analyses described in Section 2.6. The selected five features were used to retrain and test RF classifiers using the same outer-fold partitions as used for the corresponding full-descriptor models. The models using the selected five descriptors are referred to as Top-5 models. The Top-5 models were assessed for both self-prediction (the spin state for which the features were identified) and cross-prediction (the opposite spin state) regimes, with the same inner 5-fold MCC-optimized hyperparameter search and three random seeds as above. As a supplementary robustness analysis, we performed a group-based nested CV in which crystallographically distinct entries sharing the same Fe­(II)–N_6_ coordination core (i.e., identical ligand sets but different counterions, solvents, or polymorphs) were treated as a single group. Group-aware splitting in both the outer and inner CV loops ensured that no complexes with the same Fe­(II)–N_6_ coordination core appeared in both the training and test sets. The detailed protocol and results are provided in the Supporting Information.

### 2.6 Model Interpretation and Feature Importance

Random Forest (RF) models were built using the Scikit-learn^45,46^ library. Five outer folds were defined once and reused identically across all descriptor sets. Model training was repeated three times, yielding 15 RF models (3 × 5 folds) per descriptor set.

To identify important descriptors, descriptor filtering was first applied to the entire data set to define a candidate feature set, in which descriptors with nonfinite values, low variance, or high pairwise correlation (|r| ≥ 0.90) were removed.

A three-times 5-fold CV procedure was then performed using this feature set. Impurity-based feature importances were extracted from each trained RF model. The resulting 15 sets of importance values were aggregated by feature, and the mean and standard deviation were computed across models. Features were ranked by mean importance, and the top five were defined as the most important features (Top-5). RF models using only the Top-5 features (i.e., the Top-5 models) were then trained and evaluated using the same outer-fold partitions used in Section 2.5.

As a supplementary robustness analysis, we also performed a leakage-free outer-fold protocol in which descriptor filtering, feature-importance estimation, and Top-5 feature selection were conducted independently within each outer-fold training set, followed by evaluation on the corresponding held-out test set. These additional results are presented in the Supporting Information.

Furthermore, for ECFP4-based descriptors, we conducted SHAP (SHapley Additive exPlanations)^47^ analyses using RF models retrained on the entire data set with the full descriptor set. SHAP values were calculated for individual ECFP bits as a local interpretation of a model output. In this study, ECFP bits were mapped to the corresponding SMARTS patterns of substructures, and the SHAP values were projected to the atom- and bond-level contributions of the substructures. For visualization, SHAP values for each molecule were scaled by dividing by the 95th percentile of the absolute SHAP values within that molecule, to reduce the influence of extreme values. The scaled SHAP values were then distributed to atoms and bonds: for each ECFP bit, the contribution was evenly assigned to all atoms and bonds in the corresponding substructure, and atoms and bonds were colored independently within the range [-1, 1], with values outside this range clipped. To obtain a molecular-level importance score, we summed the absolute atomic and bond contributions for each molecule. This score was used to rank molecules, and the top three in each spin-state category were selected as representative examples for visualization. The highlighted colors were projected on example coordination complexes to visualize the model predictions.^48^

## 3 Results and Discussion

### 3.1 FeN_6_–SSD

#### 3.1.1 Profiles of FeN_6_–SSD

Table 1 presents a summary of the FeN_6_–SSD, which consists of 500 CSD crystal entries corresponding to Fe­(II) complexes with N_6_ coordination environments. These 500 entries included 235 distinct Fe­(II) coordination complexes when classified at the complex level, where entries sharing the same FeN_6_ coordination core (i.e., identical ligand set) were regarded as one complex regardless of differences in counterions or crystallization solvents. Unless otherwise noted, all subsequent descriptions and statistics are based on structure-level counting, in which crystallographically distinct entries are treated as separate structures even if they share the same overall composition. The data set included both complexes that exhibit SCO within the reported measurement temperature ranges and those that remain in a single-spin state over those ranges. The spin-state occurrences reported here were based on the original 500 crystal entries: 29 remaining LS, 91 remaining HS, and 380 exhibiting SCO, all within the reported measurement temperature ranges. The SCO group was further divided into spin states identified in the corresponding literature and measurement temperatures, leading to 169 HS entries, 167 LS entries, and 44 entries with mixed-spin states (i.e., crystal structures in which HS and LS molecules coexist within the unit cell).

**Table 1:** FeN_6_–SSD Profiles^a^

			Spin-crossover		
Ligand types	High- spin	Low- spin	High- spin^b^	Low- spin^b^	Mix- state^b^
2 tridentate ligands	39	20	54	80	22
2 monodentate and 1 tetradentate ligands	22	0	32	24	6
2 monodentate and 2 bidentate ligands	12	0	30	28	2
1 hexadentate ligand	8	2	19	17	4
3 bidentate ligands	6	5	14	9	2
6 monodentate ligands	3	0	16	5	8
1 bidentate and 1 tetradentate ligands	0	0	2	2	0
1 monodentate and 1 pentadentate ligands	1	2	0	0	0
3 monodentate and 1 tridentate ligands	0	0	1	1	0
4 monodentate and 1 bidentate ligands	0	0	1	1	0

			Spin-crossover		
Ion and Solvent types	High- spin	Low-spin	High- spin^b^	Low- spin^b^	Mix- state^b^
2 ions	18	11	63	57	19
no ions and no solvents	33	2	53	43	8
2 ions and 1 solvent	15	1	12	23	3
1 solvent	4	0	11	10	1
2 ions and 2 solvents	3	5	4	4	3
2 ions and 3 solvents	3	0	5	5	3
2 solvents	1	1	3	4	1
0.5 solvents	1	0	4	4	1
2 ions and 4 solvents	3	0	1	2	2
2 ions and 0.5 solvents	1	1	4	2	0
2 ions and 1.5 solvents	0	1	2	2	1
1 ion	2	1	1	1	0
3 solvents	0	2	1	1	0
2 ions and 2.5 solvents	0	2	0	0	1
2 ions and 3.25 solvents	0	0	1	1	0
1 ion and 1 solvent	0	1	1	0	0
1.5 solvents	0	0	1	1	0
2 ions and 0.66 solvents	0	0	1	1	0
2 ions and 1.09 solvents	1	0	0	0	0
2 ions and 5.33 solvents	1	0	0	0	0
2 ions and 0.2 solvents	0	0	0	1	0
3 ions and 2 solvents	0	0	0	1	0
2.5 solvents	1	0	0	0	0
2 ions and 0.25 solvents	0	0	0	1	0
2 ions and 8 solvents	1	0	0	0	0
2 ions and 5.5 solvents	0	0	0	1	0
2 ions and 5 solvents	0	0	0	1	0
2 ions and 0.75 solvents	0	0	0	0	1
2 ions and 0.33 solvents	0	0	1	0	0
2 ions and 3.5 solvents	1	0	0	0	0
2 ions and 3.42 solvents	1	0	0	0	0
2 ions and 3.15 solvents	0	0	0	1	0
2 ions and 0.67 solvents	0	1	0	0	0
0.25 solvents	1	0	0	0	0

a The number of crystal entries is reported based on (**a**) the coordinating ligands of complexes and (**b**) the crystallization solvents and counterions. For each category, the counts are classified by spin state and whether the complexes exhibit SCO within the reported measurement temperature ranges.

b Identified spin state from the crystal structure.

In FeN_6_–SSD, SCO complexes account for approximately 76%, a significantly higher proportion than those remaining in a single spin state within the reported measurement temperature ranges. This imbalance likely reflects the limited availability of temperature-dependent measurements for complexes reported as non-switchable in the literature. In many cases, only single-temperature measurements are reported without verification of the spin state across a broader temperature range. In particular, LS complexes often remain LS at low temperatures if they are already LS at room temperature. In the present study, we prioritized the reliability of spin-state assignments and included only those structures for which spin states were explicitly reported at low and high temperatures.

The coordination environments in this data set span a wide variety, from monodentate to hexadentate ligands, providing significant structural diversity in ligand types (Table 1a). As reported in previous studies, common coordination motifs, such as “two tridentate ligands”^12^ and “three bidentate ligands”,^10,24^ appeared with high frequency. The number and composition of solvent molecules and counterions in the crystals vary considerably, reflecting the diverse environment of these structures (Table 1b).

From FeN_6_–SSD, structures with intermediate or ambiguous spin states were excluded, leaving 456 entries that were used for the subsequent machine learning (ML) analyses. These entries were treated at the structure level, meaning that crystallographically distinct entries were considered independent structures even if they shared the same FeN_6_ coordination core or overall chemical composition (e.g., different counterions or solvation forms). Among these, the LS data set comprised 167 SCO-undergoing structures and 29 structures that remain LS within the reported measurement temperature ranges, while the HS data set comprised 169 SCO-undergoing structures and 91 structures that remain HS over those ranges.

#### 3.1.2 Visualization of SCO Complexes in FeN_6_–SSD Using Geometric Parameters

To assess the relationship between structural geometry and spin state, we first evaluated the OctaDist geometric descriptors for complexes in FeN_6_–SSD. Figure 1 presents the descriptor distributions for the five spin-state classes: LS, HS, SCO (LS structure), SCO (HS structure), and SCO (mixed state). The distribution of the mean metal–ligand bond length (Figure 1a) shows a distinct separation between HS and LS states, as this parameter characterizes the spin state.^1–3,35^ The temperature-dependent change in bond length may serve as a proxy for estimating SCO behavior, and indeed, some prior studies have adopted this approach.^49^ However, when comparing structures with the same spin state, i.e., HS vs SCO (HS structure) and LS vs SCO (LS structure), no significant difference in the mean bond length was observed. This indicated that the presence or absence of SCO could not be predicted solely from bond length. Similar distribution characteristics were observed for the other OctaDist descriptors (sigma, theta, zeta, and delta), highlighting the need for ML models and alternative descriptor sets.

**Figure 1:**
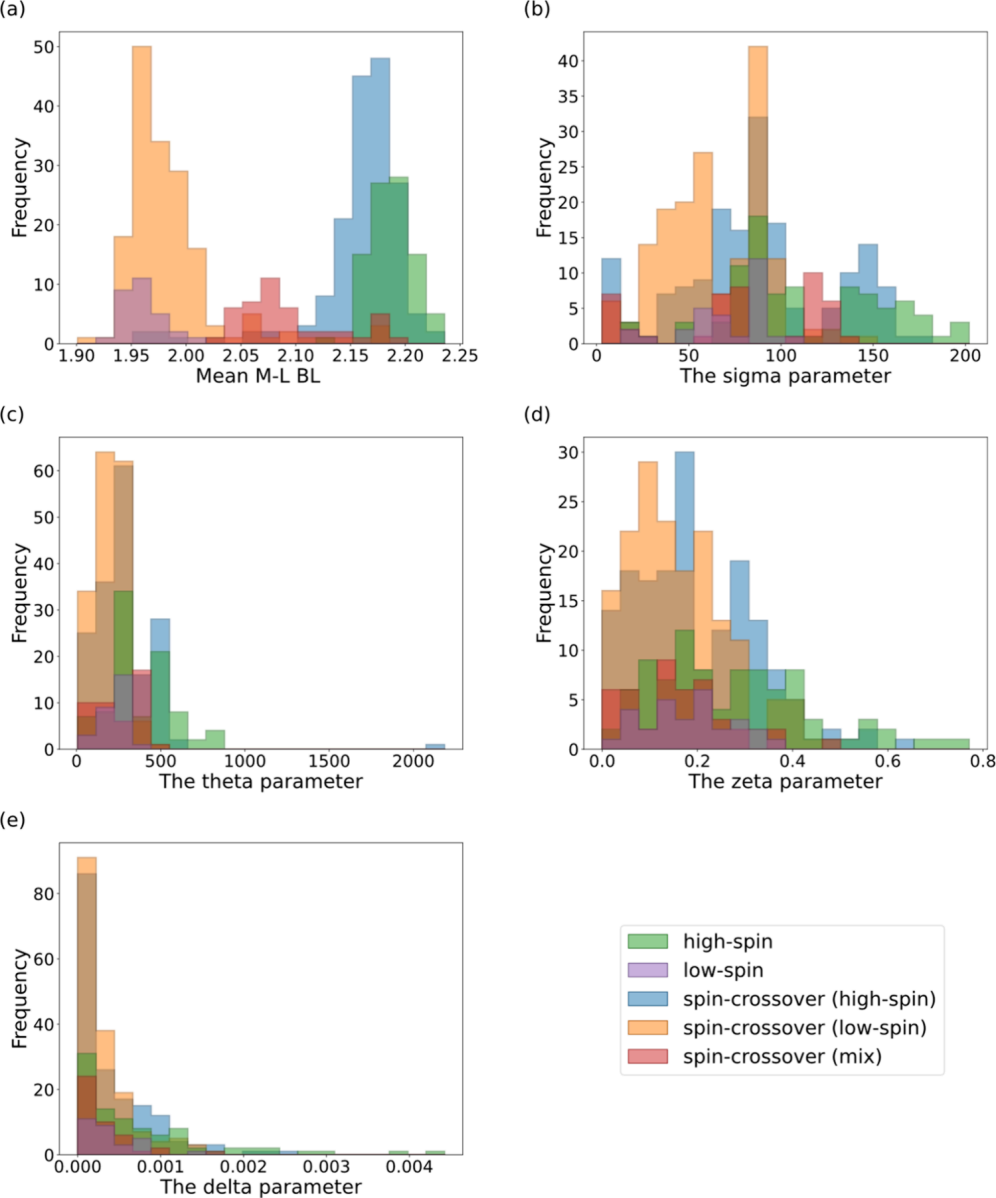
**Distributions of OctaDist descriptors.** Histograms of the five geometrical descriptors are shown, grouped by spin-state category. These descriptors are (**a**) the mean metal–ligand bond length, (**b**) the sigma, (**c**) the theta, (**d**) the zeta, and (**e**) the delta parameters. The sigma parameter is the sum of the absolute differences between the cis angles in the octahedral geometry and the ideal 90°. The theta parameter represents a cumulative torsional (twisting) distortion of the ligand sets relative to their ideal values. The zeta parameter represents additional angular distortions not fully captured by the sigma and theta parameters. The delta parameter represents a dispersion of the metal–ligand bond lengths relative to their mean value. The mean metal–ligand bond length represents the average distance between the metal center and its coordinating ligand atoms.^37^

As a supplementary analysis, we examined crystal-packing descriptors derived from the CIF structures, including density, packing coefficient, void fraction, and intermolecular contact features. As shown in Figure S2, these descriptors exhibited largely overlapping distributions across spin-state classes, indicating that simple packing descriptors alone were insufficient to distinguish SCO and non-SCO complexes.

### 3.2 Classification of SCO-Undergoing and Non-SCO Complexes in the Same Spin State

The comprehensive results for the classification of SCO-undergoing complexes with various descriptors are summarized in Table 2. Overall, MBTR-based descriptors showed strong prediction accuracy for classifying SCO-undergoing complexes from non-SCO complexes in both spin states. In the HS state, MBTR-based descriptors provided the highest prediction accuracy, whereas in the LS state, RAC-based descriptors achieved comparable or slightly higher performance. For the HS state complexes, MBTR-based descriptors achieved MCC values of approximately 0.53–0.55 and F1 scores of 0.78–0.79. For the LS state complexes, the highest performance was obtained with RAC + env, achieving MCC values of 0.43–0.45 and F1 scores of approximately 0.86. In the absence of environmental factors, the prediction accuracy remains largely unchanged. ML-based prediction of SCO-undergoing complexes from HS structures of Fe­(II) coordination complexes was more reliable than from LS structures. As a supplementary analysis, we also examined descriptors derived directly from the crystallographic CIF structures, including crystal packing descriptors (CSD-param) and periodic MBTR representations of the full crystal structure (MBTR-CIF). However, these descriptors did not improve prediction performance compared with those derived from isolated coordination complexes. The detailed results are provided in the Supporting Information (Table S4).

**Table 2:** Prediction Accuracy for SCO Classification^a^

		High spin and high-spin SCO		Low spin and low-spin SCO	
	Descriptor	MCC	F1	MCC	F1
3D	MBTR	0.53 (0.099)	0.78 (0.043)	0.40 (0.21)	0.85 (0.047)
	MBTR + env.	0.53 (0.12)	0.78 (0.051)	0.37 (0.21)	0.85 (0.045)
	MBTR-Fe	0.55 (0.066)	0.79 (0.033)	0.28 (0.17)*	0.83 (0.038)*
	MBTR-Fe + env.	0.54 (0.083)	0.79 (0.037)	0.28 (0.20)	0.83 (0.042)*
	OctaDist	0.50 (0.13)	0.77 (0.057)	0.037 (0.14)*	0.78 (0.032)*
	OctaDist + env.	0.47 (0.14)*	0.76 (0.064)	0.12 (0.20)*	0.80 (0.038)*
2D	RAC	0.26 (0.087)*	0.66 (0.039)*	0.43 (0.20)	0.86 (0.047)
	RAC + env.	0.28 (0.11)*	0.67 (0.047)*	0.45 (0.23)	0.86 (0.053)
	RAC(no stereo)	0.24 (0.10)*	0.66 (0.045)*	0.37 (0.18)	0.85 (0.043)
	RAC(no stereo) + env.	0.24 (0.095)*	0.66 (0.042)*	0.36 (0.14)	0.84 (0.039)*
	ECFP4	0.24 (0.092)*	0.66 (0.041)*	0.38 (0.23)	0.85 (0.051)
	ECFP4 + env.	0.25 (0.13)*	0.66 (0.056)*	0.33 (0.26)	0.84 (0.060)
	ECFP4-Fe	0.23 (0.11)*	0.65 (0.048)*	0.26 (0.21)*	0.82 (0.049)*
	ECFP4-Fe + env.	0.23 (0.16)*	0.65 (0.066)*	0.31 (0.19)*	0.83 (0.044)*

a The prediction accuracy was measured in Matthews correlation coefficient (MCC) and the F1 score. The task was predicting SCO complexes in the high-spin (low-spin) state. Three times 5-fold CV trials were conducted to derive the metric values. The mean and standard deviation are reported, and the * represents a statistically significant result from the Wilcoxon signed-rank test against the top-performing descriptor, and the highest average MCC value is highlighted in bold. Suffix -Fe was the subset of a descriptor set by extracting Fe-related descriptors. Suffix + env. is a concatenation of the descriptor set and the one-hot representations of solvent and counterion.

Because multiple crystallographically distinct entries may share the same Fe­(II)–N_6_ coordination core, we additionally evaluated the models using a group-based nested CV, treating such entries as a single group. This more stringent evaluation resulted in overall slightly lower prediction accuracy, but the relative performance trends among descriptor sets remained largely unchanged, confirming the robustness of the conclusions (Table S5). To further assess the potential influence of performing descriptor filtering outside the outer CV loop, we compared prediction accuracies obtained using global filtering and outer-fold filtering protocols. Similar to the group-based validation, the overall prediction accuracies and descriptor-performance trends remained largely unchanged (Table S7).

The prediction accuracy of 3D descriptors was higher than that of 2D descriptors for the HS state classification task, but no clear difference was observed for the LS state. Interestingly, OctaDist failed to identify SCO-undergoing complexes in the LS state, indicating that there is no difference in the geometrical parameters between the two classes in this spin state. Similarly, MBTR-Fe achieved prediction accuracy comparable to that of MBTR in the HS state, whereas its performance decreased in the LS classification task, indicating that Fe-centered geometric information alone is insufficient to distinguish SCO activity in LS structures. ECFP-Fe achieved moderate prediction accuracy, suggesting that ligand patterns surrounding the Fe center contain useful information even without explicit geometric descriptors. For the HS state, a 3D descriptor set based on MBTR remained effective, possibly because it can represent chemical structural patterns via binned interatomic distances. In contrast, for the LS state, a 2D RAC descriptor achieved the highest accuracy among the tested descriptors, although no statistically significant difference was observed compared with MBTR.

Overall, incorporating environmental information (counterions and solvents) had only a limited impact on prediction accuracy. A modest improvement was observed for RAC descriptors, particularly in the LS classification task, whereas most other descriptor sets showed little change when environmental information was included. This suggests that models using RAC leveraged the environmental information to compensate for the lack of geometric information.

Taken together, these results demonstrate that geometric descriptors—particularly MBTR—provide the most reliable predictive performance for HS complexes. In contrast, the classification of LS complexes is more challenging and appears to rely more heavily on chemical descriptors such as RAC, suggesting that the relative importance of geometric and chemical features varied with spin state. For LS systems, however, chemical descriptors such as RAC also achieved prediction accuracies comparable to those of MBTRs, suggesting that the contribution of chemical features to SCO behavior depended on spin state. The contrasting role of bulk features further underscores the complexity of environmental effects on SCO behavior. To better understand these trends, we investigated which descriptors are responsible for the classification outcomes through feature-importance analysis.

### 3.3 Feature Importance and Model Interpretability

To understand descriptors’ contributions to the prediction models, feature importance values from the 15 RF models described in Section 2.6 were collected (Figures 2–5), where descriptor set-wise boxplots for the highly contributing descriptors (Top 5) are presented. Figure 2 reports the feature importance of the SCO classification models in the HS state, and Figure 3 shows the feature importance in the LS state. Figures 4 and 5 also present the feature importance of the SCO classification models in the HS and LS states, respectively, but using the descriptor sets combined with one-hot vectors representing environmental factors (+env).

**Figure 2:**
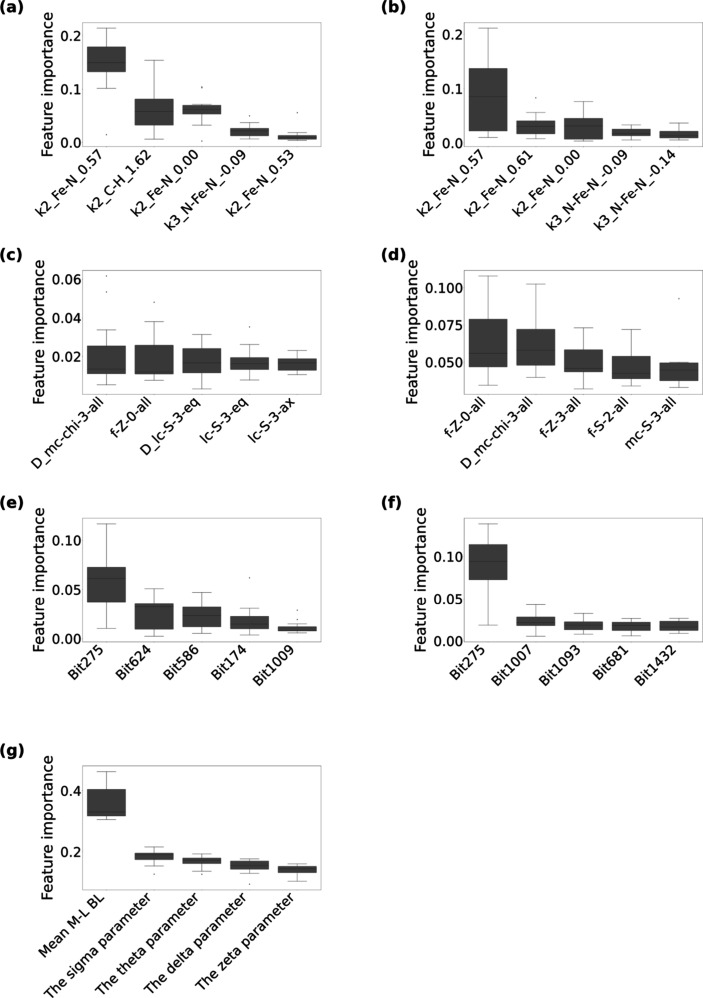
**The top five contributed descriptors for the high-spin and SCO classification models using (a) MBTR, (b) MBTR-Fe, (c) RAC, (d) RAC (no stereo), (e) ECFP4, (f) ECFP4-Fe, (g) OctaDist.** Categorized in descriptors, each boxplot summarizes the 15 feature importance values calculated based on the 15 random forest models (the three repetitions of 5-fold cross-validated models), where the importance score is defined as the mean impurity decrease within decision trees. In MBTR and MBTR-Fe, k represents the interaction order (i.e., the number of atoms involved in the geometric feature), followed by an atom tuple representing the chemical elements involved, and the real number represents the value of the geometric feature (e.g., distance or angle). For example, k2_Fe–N_0.53 represents a pairwise interaction (k = 2) between Fe and N atoms, where the value 0.53 corresponds to the inverse distance (1/Å), indicating a distance of approximately 1.89 Å. As k1, the atomic number function was used, k2, the inverse distance, and k3, the cosine of the angle. MBTR-Fe is a subset of MBTR that includes only descriptors involving iron (Fe) atoms. RAC encodes physicochemical and topological features—such as bond connectivity (i.e., graph-based bond distances), nuclear charge, electronegativity, and covalent radius—based on the molecular graph. It includes both metal-centered and ligand-centered features and is specifically designed for transition metal complexes. Although RAC primarily relies on graph-based bond distances, it also incorporates stereochemical details, such as axial and equatorial distinctions. For comparison, RAC (no stereo) is a simplified version that omits all stereochemical information and relies solely on 2D molecular graph features, thereby enabling explicit assessment of spatial effects on SCO prediction. In ECFP4, each bit corresponds to a specific substructure represented as a SMARTS pattern: Bit275: [#7D3v4 + 1H0R]­(-[#26D6v6 + 0H0R])­(:[#6D3v4 + 0H0R]):[#6D3v4 + 0H0R], Bit624: [#6D2v4 + 0H1R]­(:[#7D2v3 + 0H1R]):[#6D2v4 + 0H1R], Bit586: [#7D2v3 + 0H0R], Bit 174: [#6D2v4 + 0H1R]­(:[#6D3v4 + 0H0R]):[#6D2v4 + 0H1R], Bit 1009: [#6D1v4 + 0H3R0]-[#6D3v4 + 0H0R], Bit 1007: [#7D3v4 + 1H0R]­(-[#26D6v6 + 0H0R]­(-[#7D3v4 + 1H0R])­(-[#7D3v4 + 1H0R])­(-[#7D3v4 + 1H0R])­(-[#7D3v4 + 1H0R])-[#7D3v4 + 1H0R])­(:[#7D3v3 + 0H0R]­(-[#6D3v4 + 0H0R]):[#6D2v4 + 0H1R]):[#6D3v4 + 0H0R]­(-[#6D1v4 + 0H3R0]):[#6D2v4 + 0H1R], Bit1093: [#6D3v4 + 0H0R]­(-[#7D3v3 + 0H0R]­(:[#7D3v4 + 1H0R]):[#6D2v4 + 0H1R])­(:[#7D3v4 + 1H0R]­(-[#26D6v6 + 0H0R]):[#6D3v4 + 0H0R]):[#6D2v4 + 0H1R]:[#6D2v4 + 0H1R], Bit 681: [#6D2v4 + 0H1R]­(:[#6D2v4 + 0H1R]:[#6D2v4 + 0H1R]):[#7D3v4 + 1H0R]­(-[#26D6v6 + 0H0R]):[#6D3v4 + 0H0R], Bit 1432: [#7D3v4 + 1H0R]­(-[#26D6v6 + 0H0R])­(:[#6D3v4 + 0H0R]):[#7D3v3 + 0H0R]. ECFP4-Fe includes only the fingerprint bits corresponding to substructures that contain an Fe atom. A full list of ECFP4 bit–SMARTS mappings is available in the Supporting Information (Table S1). OctaDist quantifies structural distortions in octahedral coordination complexes. These include angular distortions (sigma, theta, and delta parameters) and bond lengths. The OctaDist description is identical to that of Figure 1.

**Figure 3:**
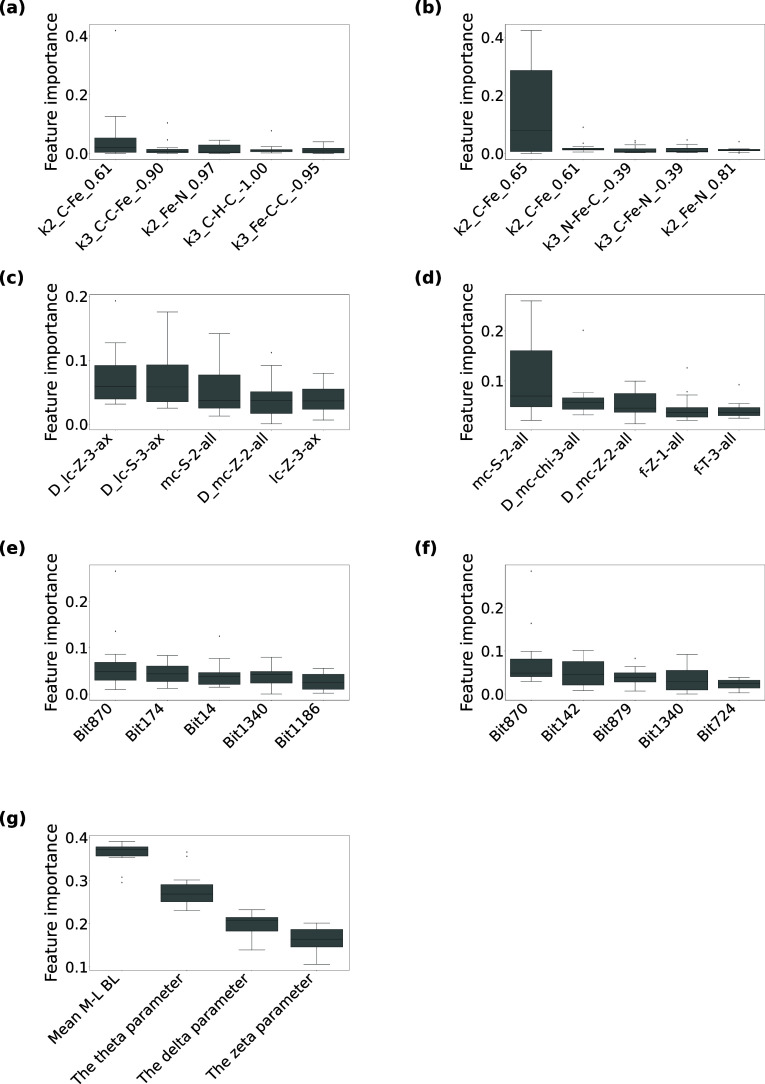
**The top five contributed descriptors for the low-spin and SCO classification models using (a) MBTR, (b) MBTR-Fe, (c) RAC, (d) RAC (no stereo), (e) ECFP4, (f) ECFP4-Fe, (g) OctaDist.** Each boxplot shows 15 feature importance values calculated based on the 15 random forest models (three repetitions of 5-fold cross-validation). The same explanation can be applied as in Figure 2. For ECFP4 (-Fe), the combinations of high bits and SMARTS patterns are Bit870: [#7D3v4 + 1H0R]­(-[#26D6v6 + 0H0R]­(-[#7D3v4 + 1H0R])­(-[#7D3v4 + 1H0R])­(-[#7D3v4 + 1H0R])­(-[#7D3v4 + 1H0R])-[#7D3v4 + 1H0R])­(:[#6D2v4 + 0H1R]:[#6D2v4 + 0H1R]):[#6D3v4 + 0H0R]­(:[#6D3v4 + 0H0R]):[#6D3v4 + 0H0R], Bit14: [#6D2v4 + 0H0R0], Bit1340: [#6D2v4 + 0H2R]­(-[#7D4v4 + 1H0R]­(-[#6D2v4 + 0H2R])­(-[#6D2v4 + 0H2R])-[#26D6v6 + 0H0R])-[#6D2v4 + 0H2R]-[#7D4v4 + 1H0R], Bit1186: [#6D3v4 + 0H0R]­(-[#6D3v4 + 0H0R])­(-[#6D1v4 + 0H3R0])=[#7D3v4 + 1H0R], Bit142: [#7D2v3 + 0H0R0]­(-[#26D6v6 + 0H0R])=[#6D2v4 + 0H0R0], Bit879: [#6D3v4 + 0H0R]­(:[#7D3v4 + 1H0R]­(-[#26D6v6 + 0H0R]):[#6D2v4 + 0H1R])­(:[#6D3v4 + 0H0R]­(:[#6D2v4 + 0H1R]):[#6D2v4 + 0H1R]):[#6D3v4 + 0H0R]­(:[#6D3v4 + 0H0R]):[#7D3v4 + 1H0R], Bit724: [#26D6v6 + 0H0R]­(-[#7D3v4 + 1H0R]­(:[#6D3v4 + 0H0R]):[#6D2v4 + 0H1R])­(-[#7D3v4 + 1H0R]­(:[#6D3v4 + 0H0R]):[#6D2v4 + 0H1R])­(-[#7D3v4 + 1H0R]­(:[#6D3v4 + 0H0R]):[#6D2v4 + 0H1R])­(-[#7D3v4 + 1H0R]­(:[#6D3v4 + 0H0R]):[#6D2v4 + 0H1R])­(-[#7D3v4 + 1H0R]­(:[#6D3v4 + 0H0R]):[#6D2v4 + 0H1R])-[#7D3v4 + 1H0R]­(:[#6D3v4 + 0H0R]):[#6D2v4 + 0H1R].

**Figure 4:**
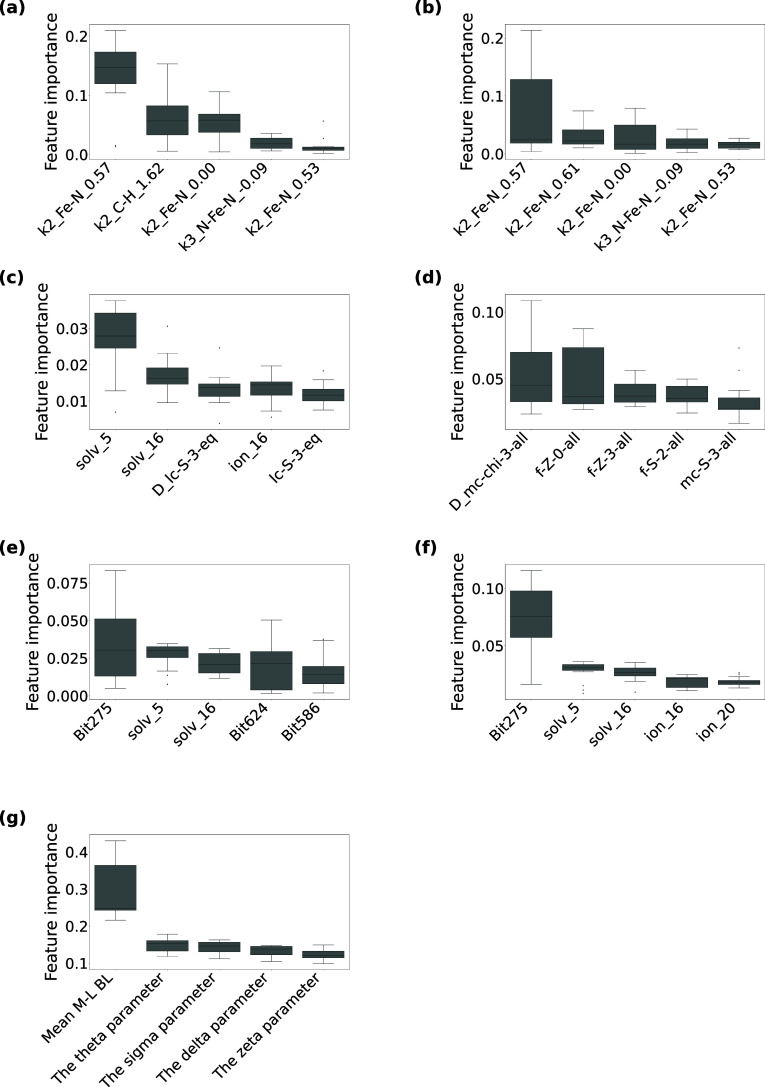
**The top five contributed descriptors for the high-spin and SCO classification models using (a) MBTR, (b) MBTR-Fe, (c) RAC, (d) RAC (no stereo), (e) ECFP4, (f) ECFP4-Fe, (g) OctaDist, all concatenated with environmental features (″+ env.″).** Each boxplot shows 15 feature importance values calculated based on the 15 random forest models (three repetitions of 5-fold cross-validation). The explanation of descriptors follows that of Figure 2. “+ env.” refers to the concatenation of each descriptor with one-hot representations of crystal solvents and counterions. Among these environmental features, the following variables exhibited particularly high importance in this figure: solv_5, corresponding to CC#N; solv_16, representing CO; ion_16, corresponding to [B-]­(F)­(F)­(F)­F; and ion_20, which denotes [Cl-].

**Figure 5:**
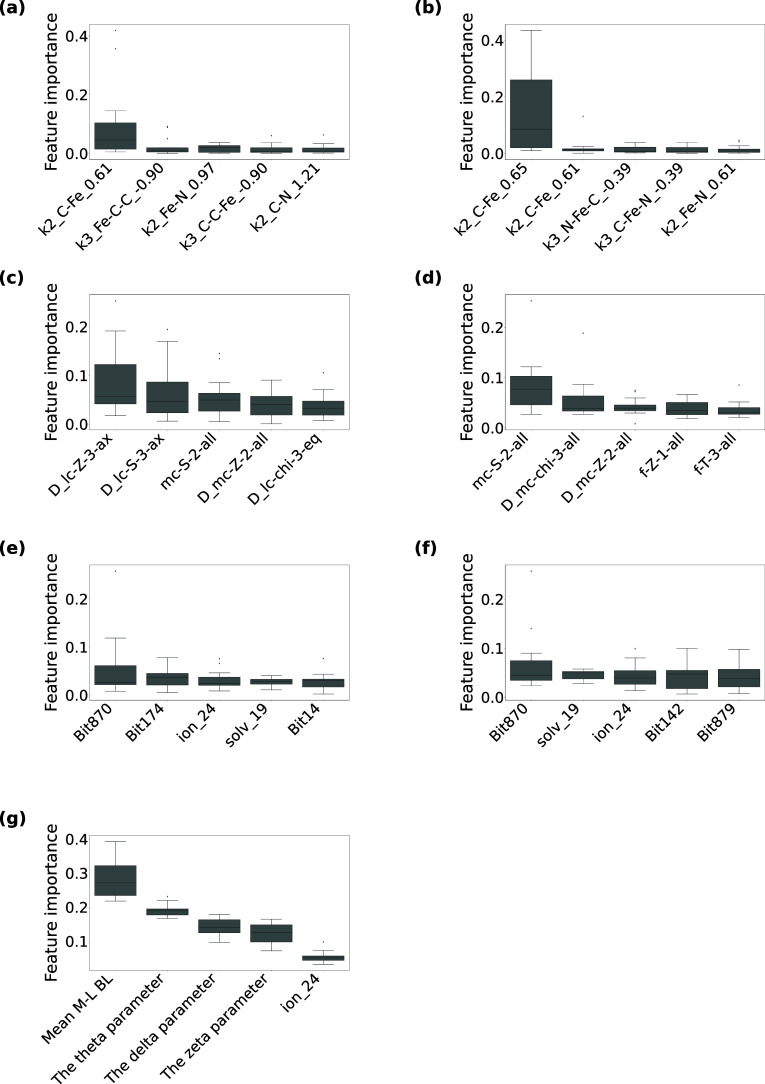
**The top five contributed descriptors for the low-spin and SCO classification models using (a) MBTR, (b) MBTR-Fe, (c) RAC, (d) RAC (no stereo), (e) ECFP4, (f) ECFP4-Fe, (g) OctaDist all concatenated with environmental features (″+ env.″).** Each boxplot shows 15 feature importance values calculated based on the 15 random forest models (three repetitions of 5-fold cross-validation). The explanation of descriptors follows that of Figures 2 and 4. For ECFP4 (and ECFP4-Fe), the bits that showed particularly high importance in this figure: ion_24, representing [O-]­Cl­(=O)­(=O)=O; and solv_19, which denotes O.

For SCO classification in the HS state, the highest-performing geometric descriptors (MBTR and OctaDist) indicated that local structural features, including Fe–N bond lengths and octahedral distortions, were important (Figures 2 and 4). These selected features directly capture bond elongation and distortions of the coordination environment characteristic of the HS state and thus serve as the primary factors distinguishing SCO-undergoing from inactive complexes. For the topological descriptor RAC, several lc and mc descriptors related to the coordination environment appeared among the selected features, suggesting that chemical information around the coordination sphere provides complementary information to the geometric descriptors. A similar trend was observed for the topological descriptor ECFP4, where fragments containing aromatic N atoms linked to the coordination sites were highly important, and environmental descriptors contributed substantially (Figure 4). Based on the fact that ECFP4 + env. showed comparable prediction accuracy to ECFP4, the selected environmental features may compensate for the limited geometric information in some descriptor sets. Overall, in HS systems, local geometric features around the Fe center were identified as dominant factors correlated with SCO activity.

For SCO classification in the LS state, geometric descriptors exhibited lower overall importance than in the HS state (Figures 3 and 5). In particular, in the MBTR descriptors, features representing the coordination environment were only sparsely represented among the top five, indicating that SCO-undergoing and inactive LS complexes showed little pronounced structural difference. For the RAC descriptors, several lc and mc descriptors related to the coordination environment were among the most important features, as was the case for the HS regime. Topological descriptor ECFP4 highlighted not only fragments containing aromatic N atoms but also fragments involving the Fe ion. These indicate that, in the LS system we used, geometric information contributed little to prediction accuracy, and that electronic and steric characteristics around the coordination sphere were more crucial. Unlike the HS state, environmental descriptors appeared among the selected features but their contributions remained secondary compared with ligand-derived descriptors. Additional feature importance analyses for descriptors derived directly from CIF structures (MBTR-CIF and CSD-param) are presented in the Supporting Information **(** Figures S3 and S4).

To provide further chemical intuition and complement the feature-importance analysis, we visualized the contributions of molecular substructures using SHAP analysis with the ECFP4 model retrained on the entire data set. Visualizing atomic contributions to the predicted value on the chemical structure is a well-established approach. We focus only on the LS system because ECFP4 achieved a relatively high prediction accuracy for this spin state.

The top three structures in each class, selected based on the molecular-level importance score derived from the SHAP analysis, are shown in Figure 6. The SHAP results for the HS state system are also provided in Figure S5. In the LS state system, fragments containing Fe-coordinated N=C=S or N=C=Se groups, together with some aromatic nitrogen donors, tended to contribute positively to SCO-undergoing prediction, underscoring the decisive role of ligand electronic and chemical properties. Overall, the SHAP analysis corroborated and refined the conclusions drawn from the feature importance analysis. In particular, the SCO classification model for the LS state was clearly governed by ligand structural features qualitatively different from those for the HS state.

**Figure 6:**
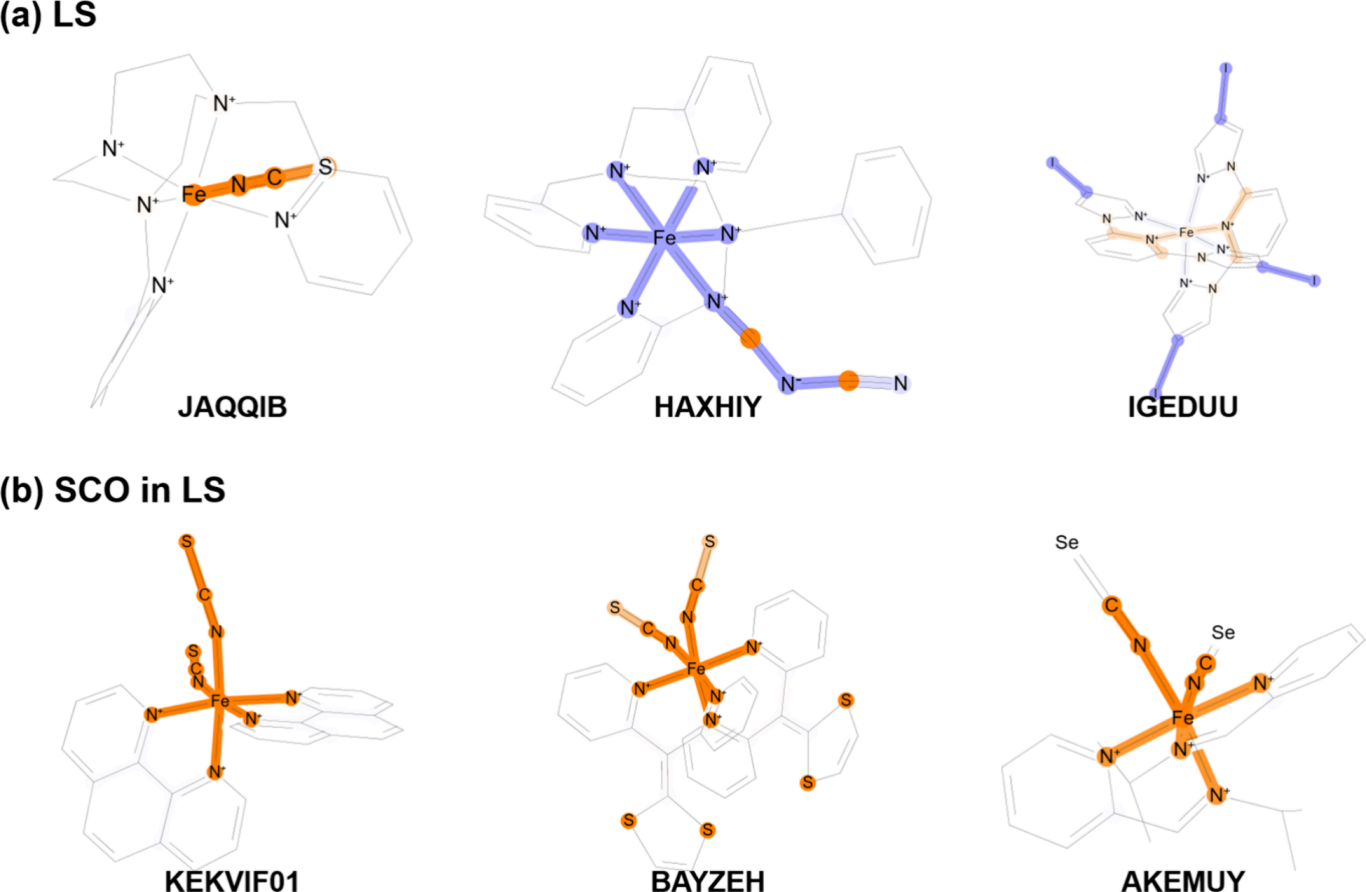
**Visualization of ECFP4 contributions derived with the SHAP analysis for the LS state classification model.** Each panel presents representative molecular structures with the highest positive (orange) and negative SHAP contributions (blue) to the classification model. Substructures with high absolute SHAP values are highlighted to visually indicate both the magnitude and the sign of their contributions. (a) The five non-SCO molecules with the highest SHAP contributions and (b) The five SCO-undergoing molecules with the highest SHAP contributions.

### 3.4 Control Calculation to Validate the Top Five Important Features

To further verify identified important descriptors, we retrained RF models using only the top five most important features and evaluated their predictive performance. There were two objectives in this study: (i) to confirm that the models trained with a small number of dominant features behave as expected for each spin state, and (ii) to examine whether commonly important characteristics exist across the high-spin and low-spin states. The results of these Top-5 feature models, including both self- and cross-prediction settings, are summarized in Table 3.

**Table 3:** Prediction Accuracy in the Matthews Correlation Coefficient (MCC) for the Control Calculation for the Importance of Selected Features^a^

		High spin and high-spin SCO		Low spin and low-spin SCO	
	Descriptor	Self	Cross	Self	Cross
3D	MBTR	0.59 (0.12)	0.31 (0.12)*	0.51 (0.19)	0.29 (0.12)
	MBTR + env.	0.59 (0.12)	0.26 (0.15)*	0.49 (0.23)	0.29 (0.12)
	MBTR-Fe	0.56 (0.086)	0.28 (0.15)*	0.35 (0.28)	0.36 (0.15)
	MBTR-Fe + env.	0.56 (0.10)	0.33 (0.11)*	0.29 (0.21)*	0.31 (0.18)
	OctaDist	0.50 (0.12)*	0.48 (0.12)	0.048 (0.14)*	0.070 (0.16)*
	OctaDist + env.	0.49 (0.12)*	0.47 (0.13)	0.054 (0.15)*	0.10 (0.17)*
2D	RAC	0.19 (0.12)*	0.17 (0.11)*	0.52 (0.22)	0.38 (0.28)
	RAC + env.	0.25 (0.13)*	0.22 (0.15)*	0.52 (0.24)	0.34 (0.23)
	RAC(no stereo)	0.22 (0.12)*	0.22 (0.095)*	0.30 (0.20)*	0.33 (0.14)
	RAC(no stereo) + env.	0.21 (0.12)*	0.22 (0.095)*	0.30 (0.20)*	0.32 (0.14)
	ECFP4	0.33 (0.11)*	0.086 (0.090)*	0.28 (0.12)*	0.074 (0.17)*
	ECFP4 + env.	0.36 (0.11)*	0.011 (0.077)*	0.23 (0.11)*	0.042 (0.17)*
	ECFP4-Fe	0.27 (0.13)*	–0.028 (0.16)*	0.26 (0.23)*	0.11 (0.19)*
	ECFP4-Fe + env.	0.24 (0.089)*	–0.11 (0.12)*	0.21 (0.13)*	–0.012 (0.14)*

a For classifications between SCO-undergoing and non-SCO complexes in either the HS or LS state, two classification models were built using only the Top-5 most important features. One is for the target task itself (Self), and the other is for the task’s counterpart (Cross). For example, when HS state complex classification is targeted, Self denotes the classification of HS state complexes, while Cross denotes HS state complex classification using only the Top-5 most important features for the LS state complex classification. The evaluation procedure and notation for MCC, F1, *, boldface, and descriptor suffixes (−Fe, + env.) are the same as in Table 2.

For the HS state models, the Top-5 feature models’ prediction accuracy was comparable to that of the full-feature models (Table 2). In particular, MBTR achieved MCC ≈ 0.59, indicating that SCO activity is primarily explained by a small set of local geometric parameters such as Fe–N bond elongation and octahedral distortions. The addition of environmental features (+env) produced negligible effects on performance.

When the models were tested under the cross-prediction setting (LS features → HS complexes), the MCC values generally decreased. This was an expected outcome, indicating that the top five features were specific to the spin states. The extent of the decrease varied among descriptors: MBTR, MBTR-Fe, ECFP4, and ECFP4-Fe showed moderate to large decreases, whereas OctaDist and RAC exhibited minor reduction. The apparent stability of OctaDist is likely due to the descriptor set comprising a small number of geometrical distortion parameters, resulting in considerable overlap between the top features selected for the HS and LS systems rather than genuine transferability across spin states.

For the LS state models, the Top-5 feature models achieved performance comparable to, or better than, the full-feature models (Table 2). In particular, RAC-based descriptors exhibited the highest accuracy. This indicates that even when only a limited number of features are used, these descriptors retain essential chemical and electronic information relevant to SCO behavior. Consequently, the overall trend remained consistent: the SCO activity in the LS state is primarily governed by chemical, electronic, and steric factors rather than by geometric parameters alone. The addition of environmental features (+env) had little effect on performance, and improvements in prediction accuracy were limited. In the cross-prediction setting (HS features → LS complexes), overall, MCC values decreased, confirming that the selected features were optimized for the respective spin state, as for the HS state.

Overall, the self- and cross-prediction experiments supported the validity of the feature-importance analysis: the top five features captured meaningful spin-state-specific factors, indicating that the dominant features differ between the HS and LS systems. Furthermore, we found no universally applicable small descriptor set for SCO classification across both spin states. For completeness, additional Top-5 model analyses for CIF-derived descriptors (MBTR-CIF and CSD-param) are provided in the Supporting Information (Table S6). In addition, results obtained using a leakage-free outer-fold protocol for feature-importance estimation and Top-5 feature selection are also provided in the Supporting Information (Table S8).

## 4 Conclusions

We created a high-quality, manually curated data set of 500 Fe­(II)–N_6_ coordination complexes, including spin states and SCO potentials, termed FeN_6_–SSD. Using this data set, we developed machine learning models to identify the structural factors underlying SCO. The classification results revealed that the key factors for predicting SCO activity differed between the spin states: in the HS regime, local geometric distortions, such as Fe–N bond elongation and octahedral deformation, were important, whereas in the LS regime, ligand-derived chemical and steric factors were important. The prediction accuracy for SCO classification in the HS regime reached an MCC value of 0.55 using MBTR. While in the LS regime, it was 0.43 with RAC. Therefore, SCO was more accurately predicted for complexes in the HS state. For well-performed descriptors, the influence of environmental factors on SCO classification was negligible.

To further explore the most influential factors, RF models were retrained using only the top five descriptors. These simplified models reproduced the expected classification trends for each spin state, demonstrating that a small number of features could capture the essential factors. Notably, MBTR maintained relatively high predictive performance for both HS and LS systems even after reduction to only five features, suggesting that a compact subset of MBTR-derived geometric descriptors encodes much of the essential SCO-related information across spin states within the present data set.

Overall, these findings indicate that SCO classification characteristics differ between HS and LS states, and that models trained in one state do not readily generalize to the other. At the same time, the robustness of the reduced MBTR models highlights a promising direction for future descriptor development: enhancing MBTR by incorporating more explicit chemical features. Such chemically enriched geometric descriptors may preserve the strong geometric sensitivity required for HS prediction while improving the chemical discriminability needed for LS systems and may ultimately provide a basis for more reliable and more transferable SCO prediction models.

## Supplementary Material



## Data Availability

The code to reproduce the results presented in this study can be found at https://github.com/natsumi-0/FeN6-SSD-ML.git, and the FeN_6_–SSD is available from the Supporting Information of this manuscript.

## Notes

Data set construction and data curation workflow for FeN_6_–SSD (Section S1), list of 575 unique ligand structures used in this study (Table S1), workflow for creating FeN_6_–SSD (Figure S1), summary of FeN_6_–SSD components (Table S2), excluded crystal structures during curation (Table S3); crystal packing descriptors and CIF-based descriptor analysis (Section S2), distributions of crystal packing descriptors derived from CIF structures (Figure S2), prediction accuracies of SCO classification models using CIF-derived descriptors (Table S4), prediction accuracies obtained using group-based nested cross-validation (Table S5), feature importance of CIF-derived descriptors for HS and LS classification models (Figures S3 and S4), MCC values of Top-5 feature models (Table S6); visualization of ECFP4 contributions based on SHAP analysis for HS state classification models (Figure S5) for feature importance analysis (Section S3); comparison of descriptor filtering protocols (Table S7) and leakage-free Top-5 model evaluation (Table S8) for robustness analyses of model evaluation (Section S4) (PDF)
