# Supplementary material for: Machine Learning Prediction for Fe(II) Spin-Crossover Complex in the Same Spin State Using Geometrical and Topological Descriptors
Source: J Chem Inf Model. 2026 Apr 16;66(8):4620–35. doi: 10.1021/acs.jcim.6c00219 (PMC13126638; doi:10.1021/acs.jcim.6c00219)
Supplement: Supplementary file 1 [file ci6c00219_si_001.pdf]

Supporting Information

for

Machine Learning Prediction for Fe(II) Spin-Crossover Complex in the Same Spin State Using Geometrical and Topological Descriptors

Natsumi Okawa[1] and Tomoyuki Miyao[2,1]*

[1]Graduate School of Science and Technology, Nara Institute of Science and Technology, Ikoma, Nara 630-0192, Japan.

[2]Data Science Center, Nara Institute of Science and Technology, 8916-5 Takayama-cho, Ikoma, Nara, 630-0192, Japan.

*Corresponding author:

E-mail: miyao@dsc.naist.jp

Contents of Supporting Information

Section S1. Dataset Construction and Data Curation

S1.1 Structural Preprocessing and File Conversion

S1.2 Separation of Structural Components

S1.3 Ligand Extraction and Denticity Assignment

S1.4 Summary of Structural Components

Table S1. List of 575 unique ligand structures used in this study

Figure S1. Workflow for creating FeN_6–SSD

Table S2. Components of FeN_6–SSD (500 entries)

Table S3. Excluded crystal structures during curation for FeN_6–SSD

Section S2. Crystal Packing and CIF-Based Descriptor Analysis

S2.1 Crystal Packing Descriptors Derived from CIF Structures

S2.2 Periodic MBTR Representation of CIF Structures

Figure S2. Distributions of crystal packing descriptors derived from CIF structures

Table S4. Prediction accuracies for SCO classification using descriptors derived directly from CIF structures

Table S5. Prediction accuracies for SCO classification obtained using group-based nested cross-validation

Figure S3. The top five contributed descriptors for the SCO classification models in the high-spin (HS) state using descriptors derived directly from CIF structures: (a) MBTR-CIF and (b) CSD-param

Figure S4. The top five contributed descriptors for the SCO classification models in the low-spin (LS) state using descriptors derived directly from CIF structures: (a) MBTR-CIF and (b) CSD-param

Table S6. MCC values of Top-5 feature models for CIF-derived descriptors (MBTR-CIF and CSD-param)

Section S3. Feature Importance Analysis

Figure S5. Visualization of ECFP4 contributions based on SHAP analysis for the HS state classification models

Section S4. Robustness Analyses of Model Evaluation

Table S7. Prediction accuracies obtained using global filtering and outer-fold filtering protocols

Table S8. Prediction accuracies of Top-5 models obtained using a leakage-free outer-fold protocol

Section S1. Dataset Construction and Data Curation

The overall procedure for constructing FeN_6–SSD, including ligand selection, spin-state assignment, and structural extraction, is described in detail in the main text (**Sections 2.1.1–2.1.3**). This section provides additional methodological details necessary to reproduce the preprocessing, file conversion, and component separation processes, which were not described in the main text for brevity. The final curated dataset, FeN_6–SSD, consisting of 500 Fe(II)–N_6 mononuclear complexes with explicitly assigned spin states, is provided as **Table S2** in the Supporting Information. **Table S2** includes the CSD[1] refcodes, assigned spin states, CIF spin states, and reference numbers for each entry.

S1.1 Structural Preprocessing and File Conversion

To ensure robust and reproducible structural separation from CSD entries, several chemoinformatics toolkits were employed, including the OpenEye Toolkit[2] and MOE[3].
When the OpenEye Toolkit was directly applied to crystal structure files obtained from the CSD (the CIF or Mol2 file formats), bonding information was occasionally lost or incomplete, particularly for structures exhibiting disorder or containing multiple components within a single unit cell.
To address these issues, a multi-route preprocessing workflow was implemented as follows:

- 362 structures – The Mol2 files were reloaded and re-saved using MOE , and then processed using the OpenEye Toolkit. This route proved to be the most reliable and was adopted as the primary workflow.
- 109 structures – The Mol2 files were directly processed using the OpenEye Toolkit without any issue.
- 12 structures – The CIF files were directly processed and successfully parsed.
- 17 structures – Manual bond corrections were performed using Mercury[4] due to persistent connectivity issues after automated processing.

This combined workflow ensured high-fidelity separation of coordination complexes from non-complex components (e.g., solvents and counterions) and minimized data loss arising from incomplete bonding information.

S1.2 Separation of Structural Components

Each preprocessed crystal structure was decomposed into coordination complexes, counterions, and crystallization solvents. Counterions and solvents were identified based on their formal charges and labeled with the prefixes “ion_” and “solv_”, respectively. When automatic classification failed due to structural disorder or ambiguous bonding

environments, manual corrections were conducted using Mercury and confirmed visually. The connectivity of each atom was defined based on the coordination to Fe(II).
Fe-centered coordination complexes were isolated and used as the basis for extracting the Fe–N_6 mononuclear species that constitute FeN_6–SSD (see main text, Section 2.1.3).

S1.3 Ligand Extraction and Denticity Assignment

Ligands were extracted from the coordination complexes based on their direct connection to the Fe center. Each ligand was saved as an individual Mol2 file. Manual inspection was performed to determine the denticity (coordination number) based on the number of donor atoms and their bonding patterns. This manual check was especially important for ligands with bridging atoms or conjugated systems, where automated parsing sometimes misidentified the donor connectivity.

S1.4 Summary of Structural Components

From the curated dataset, the types of structural components—coordination complexes, ligands, counterions, and solvents—were organized. For counterions and solvents, quantitative information was derived directly from the chemical formula field reported in the CSD.
Two types of indicators were constructed based on this information:

1. Count data – representing the stoichiometric numbers of each component exactly as reported in the chemical formula field of the corresponding CSD entry. For example, for the formula $[Fe(L)_2](ClO_4)_2 \cdot 2H_2O$, the counts were recorded as 2 for ClO_4^- and 2 for H_2O.
2. Binary presence/absence indicators – representing only whether a particular counterion or solvent was included in the structure, without considering stoichiometric quantities.

These indicators were later used as environmental features in the machine learning model development.

Table S1. List of 575 unique ligand structures used in this study. Each ligand is represented by its SMILES notation and the reference number(s) of the review article(s) from which it was extracted. Ligands that could not be expressed in SMILES (e.g., ferrocene) were excluded. Crystal structures were queried in the CSD using the SMILES together with Fe through the CSD Python API; when no hit was obtained, the ligand was searched by drawing it in ConQuest.

SMILES	Ref.
N1(C2=NC(N3N=CC=C3)=CC=C2)N=CC=C1	5
CC(C=C1)=NN1C2=CC=CC(N3N=C(C)C=C3)=N2	5
CC(C)C1=NN(C2=NC(N3N=C(C(C)C)C=C3)=CC=C2)C=C1	5
O=C(OCC)C1=NN(C2=NC(N3N=C(C(OCC)=O)C=C3)=CC=C2)C=C1	5
C1(C2=CC=CC=C2)=NN(C3=NC(N4N=C(C5=CC=CC=C5)C=C4)=CC=C3)C=C1	5
OCCC1=NN(C2=NC(N3N=C(CCO)C=C3)=CC=C2)C=C1	5
N#CSCC1=CC(N2N=CC=C2)=NC(N3N=CC=C3)=C1	5
N1(C2=NC(N3N=CC=C3)=CN=C2)N=CC=C1	5
CC1=CN(C2=NC(N3N=CC(C)=C3)=CC=C2)N=C1	5
C12=C(C=CC=C2)N(C3=NC(N4N=CC5=C4C=CC=C5)=CC=C3)N=C1	5
ClC1=CN(C2=NC(N3N=CC(Cl)=C3)=CC=C2)N=C1	5
BrC1=CN(C2=NC(N3N=CC(Br)=C3)=CC=C2)N=C1	5
OCC1=CC(N2N=CC=C2)=NC(N3N=CC=C3)=C1	5
O=[N+](C1=CC(N2N=CC=C2)=NC(N3N=CC=C3)=C1)[O-]	5
IC1=CC(N2N=CC=C2)=NC(N3N=CC=C3)=C1	5
CC(SC(C=C1)=CC=C1C#CC2=CC(N3N=CC=C3)=NC(N4N=CC=C4)=C2)=O	5
OC(C=C1)=CC=C1C2=CC(N3N=CC=C3)=NC(N4N=CC=C4)=C2	5
N1(C2=NC(N3N=CC=C3)=CC(C4=CC=NC=C4)=C2)N=CC=C1	5
N1(C2=NC(N3N=CC=C3)=CC(C4=CN=CC=C4)=C2)N=CC=C1	5
CC1=NN(C2=NC(N3N=C(C)C=C3)=CN=C2)C=C1	5
CC1=NN(C2=NC(N3N=CC=C3)=CC=C2)C(C)=C1	5
CC(C=C(C)C=C1C)=C1C2=NN(C3=NC(N4N=C(C5=C(C)C=C(C)C=C5C)C=C4)=CC=C3)C=C2	5
O=C(OCC)C1=CN(C2=NC(N3N=CC(C(OCC)=O)=C3)=CC=C2)N=C1	5
IC1=CN(C2=NC(N3N=CC(I)=C3)=CC=C2)N=C1	5
Cl[Pd+]12[N]3=CC=CC=C3C[N](CC4=[N]2C=CC=C4)1CC5=CC(N6N=CC=C6)=NC(N7N=CC=C7)=C5	5

CC1=NN(C2=NC(N3N=C(C)C=C3C)=CN=C2)C(C)=C1	5
CC(C=C(C)C=C1C)=C1C2=NN(C3=NC(N4N=C(C5=C(C)C=C(C)C=C5C)C=C4)=CN=C3)C=C2	5
Cc1nn(c(c1C)C)[BH-](n1nc(c(c1C)C)C)n1nc(c(c1C)C)C	5
FC(F)(F)c3ccn([BH-](n1ccc(C(F)(F)F)n1)n2ccc(C(F)(F)F)n2)n3	5
c7cn([B-](n2ccc(C1CC1)n2)(n4ccc(C3CC3)n4)n6ccc(C5CC5)n6)nc7C8CC8	5
CC1=NN(C=C1)[B-](N2C=CC(C)=N2)(C3=CC=CC=C3)N4N=C(C)C=C4	5
CC1=NN(C=C1)[B-](N2C=CC(C)=N2)(C3=CC=C(I)C=C3)N4N=C(C)C=C4	5
[BH-](N1C=CC(C2=CC=CC=C2)=N1)(N3C=CC(C4=CC=CC=C4)=N3)N5N=C(C6=CC=CC=C6)C=C5	5
CC1=NN(C=C1)[B-](N2C=CC(C)=N2)(N3C=CC(C)=N3)N4N=C(C)C=C4	5
N#CC(C(C1=CC=CC=C1)=N2)=CN2[BH-](N3C=C(C#N)C(C4=CC=CC=C4)=N3)N5N=CC(C#N)=C5C6=CC=CC=C6	5
CC1=NN(C=C1)[BH-](N2C=CC(C)=N2)N3N=C(C)C=C3	5
CC1=NN(C(C)=C1)[BH-](N2C(C)=CC(C)=N2)N3N=C(C)C=C3C	5
[BH-](N1C=CC(C2CC2)=N1)(N3C=CC(C4CC4)=N3)N5N=C(C6CC6)C=C5	5
Cc4ccn([B-](c1ccc(C#C[Si](C)(C)C)cc1)(n2ccc(C)n2)n3ccc(C)n3)n4	5
Cc5ccn([B-](c2ccc(C#Cc1ccccc1)cc2)(n3ccc(C)n3)n4ccc(C)n4)n5	5
C#Cc4ccc([B-](n1ccc(C)n1)(n2ccc(C)n2)n3ccc(C)n3)cc4	5
[BH-](N1C=CC=N1)(N2C=CC=N2)N3N=CC=C3	5
CC(C)(C)[B-](N1C=CC(C)=N1)(N2C=CC(C)=N2)N3N=C(C)C=C3	5
N1(N=CC=C1)[B-](N2C=CC=N2)(N3C=CC=N3)N4C=CC=N4	5
N1(N=CC=C1)[B-](N2C=CC=N2)(C3=CC=CC=C3)N4C=CC=N4	5
IC(C=C1)=CC=C1[B-](N2C=CC=N2)(N3C=CC=N3)N4N=CC=C4	5
[BH-](N1C(C=CC=C2)=C2C=N1)(N3C(C=CC=C4)=C4C=N3)N5N=CC6=C5C=CC=C6	5
CC(C)(C)[B-](N1C=CC=N1)(N2C=CC=N2)N3N=CC=C3	5
Cc1cc(C)n(C(n2nc(C)cc2C)n3nc(C)cc3C)n1	5
Cc1c(C)c(C)n(C(n2nc(C)c(C)c2C)n3nc(C)c(C)c3C)n1	5
Cc1cc(C)n([C-](n2nc(C)cc2C)n3nc(C)cc3C)n1	5
Cc1ccn(C(n2nccc2C)n3nc(C)cc3)n1	5
n1(C(n2nccc2)n3nccc3)nccc1	5
Cc1cnn(C(n2ncc(C)c2)n3ncc(C)c3)c1	5
OC(n1nccc1)(n2nccc2)n3nccc3	5
c1cccnc1	5
O=P(c1ccccc1)(c2ccccc2)OCC(n3nccc3)(n4nccc4)n5nccc5	5

n1(C(n2nccc2)(COCc3ccc(COCC(n4cccn4)(n5cccn5)n6nccc6)cc3)n7nccc7)nccc1	5
OC1=CC=CC=C1/C=N/CCNCCNCC/N=C/C2=C(O)C=CC=C2	5
OC1=CC=C(OC)C=C1/C=N/CCNCCNCC/N=C/C2=C(O)C=CC(OC)=C2	5
OC1=CC=C(C)C=C1/C=N/CCNCCNCC/N=C/C2=C(O)C=CC(C)=C2	5
OC1=CC=C(C(C)(C)C)C=C1/C=N/CCNCCNCC/N=C/C2=C(O)C=CC(C(C)(C)C)=C2	5
OC1=CC=CC=C1/C=N/CCN(CCCCCC)CCN(CCCCCC)CC/N=C/C2=C(C=CC=C2)O	5
OC1=C(Cl)C=C(Cl)C=C1/C=N/CCNCCNCC/N=C/C2=C(C(Cl)=CC(Cl)=C2)O	5
OC1=CC=C(Cl)C=C1/C=N/CCNCCNCC/N=C/C2=C(O)C=CC(Cl)=C2	5
OC1=CC=C2C(C=CC=C2)=C1/C=N/CCNCCNCC/N=C/C3=C(O)C=CC4=C3C=CC=C4	5
OC1=C(OC)C=CC=C1/C=N/CCNCCCNCC/N=C/C2=C(O)C(OC)=CC=C2	5
OC1=C(OC)C=C(OC)C=C1/C=N/CCNCCCNCC/N=C/C2=C(O)C(OC)=CC(OC)=C2	5
OC(C=CC=C1)=C1/C=N/C2=C3C(C=CC=N3)=CC=C2	5
OC(C=CC=C1)=C1/N=C/C2=CC=CC=N2	5
OC1=CC=CC=C1/C=N/CCNCCCNCC/N=C/C2=C(O)C=CC=C2	5
OC(C=CC1=C2C=CC=C1)=C2/C=N/C3=C4C(C=CC=N4)=CC=C3	5
OC(C=CC(Cl)=C1)=C1/C=N/C2=C3C(C=CC=N3)=CC=C2	5
OC(C(OC)=CC=C1)=C1/C=N/C2=C3C(C=CC=N3)=CC=C2	5
O=C(OCC)/C(C(C)=O)=C/NC1=CC=CC=C1N/C=C(C(C)=O)/C(OCC)=O	5
O=C(C)/C(C(C)=O)=C/NC1=CC=CC=C1N/C=C(C(C)=O)/C(C)=O	5
CN1C=NC=C1	5
CC1=NC=CN1	5
O=C(OCC)/C(C(C1=CC=CC=C1)=O)=C/NC2=CC=CC=C2N/C=C(C(C3=CC=CC=C3)=O<br>)/C(OCC)=O	5
O=C(OCC)/C(C(C)=O)=C/NC1=CC(N/C=C(C(C)=O)/C(OCC)=O)=C(N/C=C(C(C)=O)/C(<br>OCC)=O)C=C1N/C=C(C(C)=O)/C(OCC)=O	5
O=C(C)/C(C(C)=O)=C/NC1=CC(N/C=C(C(C)=O)/C(C)=O)=C(N/C=C(C(C)=O)/C(C)=O)<br>C=C1N/C=C(C(C)=O)/C(C)=O	5
C1=CN=CN1	5
C1(C2=CC=CC=C2)=CC=NC=C1	5
C1(C2=CC=NC=C2)=CC=NC=C1	5
C1(CNCC2=CC=NC=C2)=CC=NC=C1	5
C1(/C=C/C2=CC=NC=C2)=CC=NC=C1	5
N#CC1=CC=NC=C1	5
CN(C)C1=CC=NC=C1	5
C1(CCCC2=CC=NC=C2)=CC=NC=C1	5

CN1C=CN=C1/C=N/CCN(CC/N=C/C2=NC=CN2C)CC/N=C/C3=NC=CN3C	5
CC1=CC=CC(/C=N/CCN(CC/N=C/C2=NC(C)=CC=C2)CC/N=C/C3=CC=CC(C)=N3)=N1	5
CC1=C(C=CC(/C=N/CCN(CC/N=C/C2=CC=C(C(C)=N2)OCCCCCC)CC/N=C/C3=NC(C)=C(C=C3)OCCCCCC)=N1)OCCCCCC	5
C1(/C=N/CCN(CC/N=C/C2=NC=CC=C2)CC/N=C/C3=CC=CC=N3)=NC=CC=C1	5
C1(/C=N/CCN(CC/N=C/C2=CN=CN2)CC/N=C/C3=CN=CN3)=CN=CN1	5
CC1=NC=C(N1)/C=N/CCN(CC/N=C/C2=CN=C(C)N2)CC/N=C/C3=CN=C(C)N3	5
C1(/C=N/CCN(CC/N=C/C2=NC=CN2)CC/N=C/C3=NC=CN3)=NC=CN1	5
C=CCN1C(/C=N/CCN(CC/N=C/C2=CN=CN2CC=C)CC/N=C/C3=CN=CN3CC=C)=CN=C1	5
CCCCCCOC(C=N1)=CC=C1/C=N/CCN(CC/N=C/C2=NC=C(OCCCCCC)C=C2)CC/N=C/C3=CC=C(OCCCCCC)C=N3	5
N#CS	5
C(=C/c1ccncc1)/c2ccccc2	5
C(=C¥c1ccncc1)/c2ccccc2	5
C1C=NOC=1	5
CN1C=NN=N1	5
CCN1C=NN=N1	5
FCCN1C=NN=N1	5
ClCCN1C=NN=N1	5
BrCCN1C=NN=N1	5
ICCN1C=NN=N1	5
CCCN1C=NN=N1	5
CC(C)N1C=NN=N1	5
CCCCCCCCN1C=NN=N1	5
CCCCCCCCCCN1C=NN=N1	5
CCCCCCCCCCCCN1C=NN=N1	5
N#C[B-](c1ccccc1)(c2ccccc2)c3ccccc3	5
N#C[O]	6
NCc1ccccn1	6
[2H]N([2H])Cc1ccccn1	6
CC(N)c1ccccn1	6
[R]/N=C/c1ccccn1	6
C/N=C(C)/c1ccccn1	6
C/N=C/c1cccc(C)n1	6

Cc1cccc(/C=N/N(C)C)n1	6
Cc2cccc(/C=N/c1ccc(I)cc1)n2	6
Cc1cccc(/C=N/C(C)C)n1	6
Cc2cccc(/C=N/c1ccccc1)n2	6
Cc2cccc(/C=N/c1ccccc1C)n2	6
Cc2ccc(/N=C/c1cccc(C)n1)cc2	6
Cc2cccc(/C=N/c1c(C)cccc1C)n2	6
Cc2cccc(/C=N/c1ccc(Cl)cc1)n2	6
COc2ccc(/N=C/c1cccc(C)n1)cc2	6
CCCCCCCCCCCCCCCCOc2ccc(/N=C/c1cccc(C)n1)cc2	6
CCCCCCCCCCCCCCCCOc2ccc(/N=C/c1cccc(C)n1)cc2OCCCCCCCCCCCCCCCC	6
CCCCCCCCCCCCCCCCOc2cc(/N=C/c1cccc(C)n1)cc(OCCCCCCCCCCCCCCCC)c2OC CCCCCCCCCCCCCCC	6
N#C[Se]	6
C(=N¥c2ccc(c1ccccc1)cc2)/c3ccccn3	6
C(#Cc2ccc(/N=C/c1ccccn1)cc2)c3ccccc3	6
C(=N¥c2ccc(/N=N/c1ccccc1)cc2)/c3ccccn3	6
C(=N¥c1ccc3c(c1)Cc2ccccc23)/c4ccccn4	6
C(=N¥c3ccc(c2ccc(c1ccccc1)cc2)cc3)/c4ccccn4	6
C(C#Cc2ccc(/N=C/c1ccccn1)cc2)#Cc3ccccc3	6
C(#Cc3ccc(C#Cc2ccc(/N=C/c1ccccn1)cc2)cc3)c4ccccc4	6
C(#Cc1cccc2ccccc12)c4ccc(/N=C/c3ccccn3)cc4	6
CC(C)c2ccc(/N=C/c1ccccn1)cc2	6
c3ccc(/N=C(c1ccccn1)/c2ccccn2)cc3	6
c2ccc(c1ccccn1)nc2	6
Cc2ccnc(c1cc(C)ccn1)c2	6
c4ccc(c3ccnc(c2cc(c1ccccc1)ccn2)c3)cc4	6
Cc2cccc(c1ccccn1)n2	6
Cc2cccc(c1cccc(C)n1)n2	6
Cc2cc(C)nc(c1cc(C)cc(C)n1)c2	6
Cc1cccnc1c2ncccc2C	6
COC(=O)c1cccnc1c2ncccc2C(=O)OC	6
c4ccc3c(c1nccc2ccccc12)nccc3c4	6
c1cnc3c(c1)ccc2cccnc23	6
Cc2cc1cccnc1c3ncccc23	6

c4ccc(c2cc1cccnc1c3ncccc23)cc4	6
Clc2cc1cccnc1c3ncccc23	6
O=N(=O)c2cc1cccnc1c3ncccc23	6
Cc1ccnc3c1ccc2cccnc23	6
Clc1ccnc3c1ccc2cccnc23	6
N#Cc1ccnc3c1ccc2cccnc23	6
CCOC(=O)c1ccnc3c1ccc2cccnc23	6
CCCCOC(=O)c1ccnc3c1ccc2cccnc23	6
Cc2c(C)c1cccnc1c3ncccc23	6
Cc1ccnc3c1ccc2c(C)ccnc23	6
Clc1ccnc3c1ccc2c(Cl)ccnc23	6
CCCCOC(=O)c1ccnc3c1ccc2c(C(=O)OCCCC)ccnc23	6
Cc3cnc2c(ccc1c(C)c(C)cnc12)c3C	6
Cc1cnc3c(c1)c(C)c(C)c2cc(C)cnc23	6
Cc3ccc2ccc1cccnc1c2n3	6
Clc3ccc2ccc1cccnc1c2n3	6
COc3ccc2ccc1cccnc1c2n3	6
Cc3ccc2ccc1ccc(C)nc1c2n3	6
c3ccc(c2ccc1ccccc1n2)nc3	6
c4ccc3nc(c2ccc1ccccc1n2)ccc3c4	6
c2cnc(c1ncccn1)nc2	6
c2cnnc(c1cccnn1)c2	6
Cc3ccnc(c2cc(/C=C/c1ccccc1)ccn2)c3	6
CCCCCCCCCCCCCCCCCc3ccnc(c2cc(/C=C/c1ccccc1)ccn2)c3	6
FC(F)(F)C(F)(F)C(F)(F)C(F)(F)C(F)(F)C(F)(F)CCCCCCCCCCCCc2ccnc(c1ccccn1)c2	6
CCCCCCCCCCCCCCCCCCOc2cc1c(CCCCCCCCCCCCCCCCC)ccnc1c3nccc(CCCCCC CCCCCCCCCCC)c23	6
O=C(NS(=O)(=O)C(F)(F)F)c2ccnc(c1cc(C(=O)NS(=O)(=O)C(F)(F)F)ccn1)c2	6
c3cnc2c(ccc1nccnc12)n3	6
c5ccc4nc3c1cccnc1c2ncccc2c3nc4c5	6
Cc2nnc(c1ccccn1)nc2C	6
c4ccc(c2nnc(c1ccccn1)nc2c3ccccc3)cc4	6
c4ccc(c3nnc(c1ccccn1)c(c2ccccn2)n3)nc4	6
c2ccc(n1cccn1)nc2	6
Cc2cc(C)n(c1ccccn1)n2	6

c2ccc(c1cc[nH]n1)nc2	6
Cc2cc(c1ccccn1)n[nH]2	6
c3ccc(c2cc(c1ccccn1)n[nH]2)cc3	6
c2ccc(c1nc[nH]n1)nc2	6
Cn2cnc(c1ccccn1)n2	6
Cc2nc(c1ccccn1)n[nH]2	6
Cc2nc(c1ccccn1)nn2C	6
Cn1cnnc1c2ccccn2	6
c2ccc(c1nccs1)nc2	6
Cc2csc(c1ccccn1)n2	6
Cc2cccc(c1nccs1)n2	6
c3ccc(c2nc1ccccc1s2)nc3	6
c2ccc(c1cscn1)nc2	6
Cc2nc(c1ccccn1)cs2	6
c2ccc(c1ncc[nH]1)nc2	6
Cn1ccnc1c2ccccn2	6
Cc2cccc(c1ncc[nH]1)n2	6
c3ccc(c2nc1ccccc1[nH]2)nc3	6
Cn3c(c1ccccn1)nc2ccccc23	6
Cc3cccc(c2nc1ccccc1[nH]2)n3	6
Cc3cccc(c2nc1ccccc1n2C)n3	6
c2ccc(C1=NCCN1)nc2	6
c3ccc(c1nnn2ccccc12)nc3	6
c4ccc(c2nc1ccccc1nc2c3ccccn3)nc4	6
Nn2c(c1ccccn1)nnc2c3ccccn3	6
Cc4cccc(n2c(c1ccccn1)nnc2c3ccccn3)c4	6
Cc4ccc(n2c(c1ccccn1)nnc2c3ccccn3)cc4	6
c2cnc(c1cc[nH]n1)cn2	6
c2cnc(c1nc[nH]n1)cn2	6
Cn2cnc(c1cnccn1)n2	6
c2csc(c1cc[nH]n1)n2	6
c2csc(c1nccs1)n2	6
c2nc(c1cscn1)cs2	6
C2CSC(C1=NCCS1)=N2	6
CC2CSC(C1=NC(C)CS1)=N2	6

CCC2CSC(C1=NC(CC)CS1)=N2	6
CC2CN=C(C1=NCC(C)S1)S2	6
C2CN=C(C1=NCCCS1)SC2	6
c2c[nH]c(c1ncc[nH]1)n2	6
c4ccc3[nH]c(c2nc1ccccc1[nH]2)nc3c4	6
C2CNC(C1=NCCN1)=N2	6
C2CN=C(C1=NCCCN1)NC2	6
C2COC(C1=NCCO1)=N2	6
CC2CN=C(C1=NCC(C)O1)O2	6
c2cnn(C1=NCCN1)c2	6
c3ccc2[nH]c(c1cscn1)nc2c3	6
[BH2-](n1cccn1)n2cccn2	6
c2cnn(Cn1cccn1)c2	6
Cc2cc(C)n(Cn1nc(C)cc1C)n2	6
c2ccc(Nc1ccccn1)nc2	6
c3ccc(C(c1ccccn1)=c2sccs2)nc3	6
O=C(c1ccccn1)c2ccccn2	6
c2c[nH]c(Cc1ncc[nH]1)n2	6
O=C(c1ncc[nH]1)c2ncc[nH]2	6
[R]c2ccc(/N=N/c1nccn1[R])cc2	6
Cc1ccccc1/N=C/c2ncc[nH]2	6
c2ccc(/N=N/c1ccccn1)nc2	6
c2ccc(/N=N/c1ccccn1)cc2	6
O=c2c1cccnc1c3ncccc23	6
CC(C)(C)c2cc(c1ccccn1)n[nH]2	6
Nc2nc(c1ccccn1)cs2	6
Cc3ccc(c2nc1ccccc1n2C)nc3	6
Cc1cccnc1c3nc2ccccc2n3C	6
[BH3-]C#N	6
N#C/C(C#N)=C(C#N)¥C#N	6
c6csc(c5ccn([BH-](n2ccc(c1cccs1)n2)n4ccc(c3cccs3)n4)n5)c6	6
Cc3cnn([BH-](n1cc(C)cn1)n2cc(C)cn2)c3	6
Brc3cnn([BH-](n1cc(Br)cn1)n2cc(Br)cn2)c3	6
Cc3cc(C)n([BH-](n1cccn1)n2cccn2)n3	6
Cc3cc(C)n([BH-](n1cccn1)n2nc(C)cc2C)n3	6

CC(C)c4ccn([B-](n1ccc(C(C)C)n1)(n2ccc(C(C)C)n2)n3ccc(C(C)C)n3)n4	6
Cc4cnn([B-](n1cccn1)(n2cc(C)cn2)n3cc(C)cn3)c4	6
C(#C[B-](n1cccn1)(n2cccn2)n3cccn3)c7ccc(COCC(n4cccn4)(n5cccn5)n6cccn6)cc7	6
c3ncn([BH-](n1cncn1)n2cncn2)n3	6
c1ccc2c(c1)nnn2[BH-](n4nnc3ccccc34)n6nnc5ccccc56	6
Cc3ccn(C(n1ccc(C)n1)n2ccc(C)n2)n3	6
Brc3cnn(C(n1cc(Br)cn1)n2cc(Br)cn2)c3	6
C[Al](c1ccccn1)(c2ccccn2)c3ccccn3	6
c4ccc(Nc3cccc(c2cccc(Nc1ccccn1)n2)n3)nc4	6
c1cnc4c(c1)c2nsnc2c3cccnc34	6
Cc4ccnc(Nc3cccc(c2cccc(Nc1cc(C)ccn1)n2)n3)c4	6
Cc1cccnc1Nc4cccc(c3cccc(Nc2ncccc2C)n3)n4	6
c6ccc5c(Nc4cccc(c3cccc(Nc1nccc2ccccc12)n3)n4)nccc5c6	6
c4ccc(c3ccc(CCc2cccc(c1ccccn1)n2)cn3)nc4	6
Cc4ccc(Nc3cccc(c2cccc(Nc1ccc(C)cn1)n2)n3)nc4	6
c4ccc(c3cccc(Nc2cccc(c1ccccn1)n2)n3)nc4	6
c4cnn(Cc3cccc(c2cccc(Cn1cccn1)n2)n3)c4	6
Cc4cc(C)n(Cc3cccc(c2cccc(Cn1nc(C)cc1C)n2)n3)n4	6
c7ccc6c(c4nc3c1cccnc1c2ncccc2c3[nH]4)c5ccccc5cc6c7	6
c1cnc5c(c1)c3nc(c2ccsc2)[nH]c3c4cccnc45	6
c1cnc5c(c1)c3nc(c2cccs2)[nH]c3c4cccnc45	6
c5cncc(c4nc3c1cccnc1c2ncccc2c3[nH]4)c5	6
c1cnc5c(c1)c3nc(c2ccncc2)[nH]c3c4cccnc45	6
COc5ccc4CCc3cc2CCc1cccnc1c2nc3c4c5c%10c(OC)ccc9CCc8cc7CCc6cccnc6c7nc8c9%10	6
FC(F)(F)c1ccccc1c5nc4c2cccnc2c3ncccc3c4[nH]5	6
c1cnc4c(c1)c2nccnc2c3cccnc34	6
Cc4cnc3c1cccnc1c2ncccc2c3n4	6
N#C[N-]C#N	6
CCOC(=O)Cn1ccnc1c2ccccn2	6
C(=Nn2c(c1ccccn1)nnc2c3ccccn3)c4cccc5ccccc45	6
CCCCCCCCCCCCCCCC(=O)Nn2c(c1ccccn1)nnc2c3ccccn3	6
N#CCCSc3sc(=C(c1ccccn1)c2ccccn2)sc3SCCC#N	6
Clc2ccc(/N=N/c1ccccc1)nc2	6
c5ccc(/N=N/c4ccc3ccc2cc(/N=N/c1ccccc1)ncc2c3n4)cc5	6

CCN1CCN=C1/N=N/c2ccc(C)cc2	6
c3ccc(c2cccc(c1ccccn1)n2)nc3	6
Cc3cccc(c2cccc(c1cccc(C)n1)n2)n3	6
c5ccc(c4cccc(c3cccc(c2cccc(c1ccccc1)n2)n3)n4)cc5	6
Cc5cc(c1ccccc1)cc(c4cccc(c3cc(c2ccccc2)cc(C)n3)n4)n5	6
c7ccc(c6cc(c1ccccc1)nc(c5cccc(c4cc(c2ccccc2)cc(c3ccccc3)n4)n5)c6)cc7	6
Cc5ccc(c4cccc(c3cccc(c2cccc(c1ccc(C)cc1)n2)n3)n4)cc5	6
c5ccc(c4cc(c1ccccc1)nc(c3cccc(c2ccccn2)n3)c4)cc5	6
c4ccc(c3cccc(c2cccc(c1ccccn1)n2)n3)nc4	6
c4ccc(Nc3ccc2ccc1cccnc1c2n3)nc4	6
c3ccc(Nc2cccc(Nc1ccccn1)n2)nc3	6
Cc5ccc(c1cccnc1c4cccc(c2ncccc2c3ccc(C)cc3)n4)cc5	6
c4ccc(c3cc(c1ccncc1)cc(c2ccccn2)n3)nc4	6
c7ccc(c6cc(c1ccc2CCC4CCCc3ccc1c2c34)cc(c5ccccn5)n6)nc7	6
CCCCCCCCCCCCCCCCOc3cc(c1ccccn1)nc(c2ccccn2)c3	6
O=n4ccc(c3cc(c1ccccn1)nc(c2ccccn2)c3)cc4	6
c3cc(c1cc[nH]n1)nc(c2cc[nH]n2)c3	6
c3cnn(Cc2cccc(Cn1cccn1)n2)c3	6
Cc3cc(C)n(Cc2cccc(Cn1nc(C)cc1C)n2)n3	6
Cc3cc(C)n(Cc2cccc(Cn1cccn1)n2)n3	6
c3cc(c1ncc[nH]1)nc(c2ncc[nH]2)c3	6
c3cc(C1=NCCN1)nc(C2=NCCN2)c3	6
c5cc(c2nc1ccccc1[nH]2)nc(c4nc3ccccc3[nH]4)c5	6
Cc5cc(c2nc1ccccc1[nH]2)nc(c4nc3ccccc3[nH]4)c5	6
Clc5cc(c2nc1ccccc1[nH]2)nc(c4nc3ccccc3[nH]4)c5	6
Oc5cc(c2nc1ccccc1[nH]2)nc(c4nc3ccccc3[nH]4)c5	6
CCCCCCCCCCCCOc5cc(c2nc1ccccc1[nH]2)nc(c4nc3ccccc3[nH]4)c5	6
c7ccc6[nH]c(c5cc(c2nc1ccccc1[nH]2)nc(c4nc3ccccc3[nH]4)c5)nc6c7	6
c%10ccc9[nH]c(c8cc(OCOCOCOCOCOCOc5cc(c2nc1ccccc1[nH]2)nc(c4nc3ccccc3[nH]4)c5)cc(c7nc6ccccc6[nH]7)n8)nc9c%10	6
Cn5c(c3cccc(c2nc1ccccc1n2C)n3)nc4ccccc45	6
COc7cc(Cn6c(c4cccc(c2nc1ccccc1n2Cc3cc(OC)cc(OC)c3)n4)nc5ccccc56)cc(OC)c7	6
COc8cc(Cn7c(c5cc(c1ccccc1)cc(c3nc2ccccc2n3Cc4cc(OC)cc(OC)c4)n5)nc6ccccc67)cc(OC)c8	6

COc8cc(Cn7c(c5cc(c1ccc(N(=O)=O)cc1)cc(c3nc2ccccc2n3Cc4cc(OC)cc(OC)c4)n5)nc6ccccc67)cc(OC)c8	6
c3cc(c1nccs1)nc(c2nccs2)c3	6
Cc3csc(c2cccc(c1nc(C)cs1)n2)n3	6
c3cc(c1cscn1)nc(c2cscn2)c3	6
Cc3nc(c2cccc(c1csc(C)n1)n2)cs3	6
c5cc(c2nc1ccccc1s2)nc(c4nc3ccccc3s4)c5	6
c5cc(c2nc1ccccc1o2)nc(c4nc3ccccc3o4)c5	6
c3cc(c1nc[nH]n1)nc(c2nc[nH]n2)c3	6
Cn3cnc(c2cccc(c1ncn(C)n1)n2)n3	6
Cc3nc(c2cccc(c1n[nH]c(C)n1)n2)n[nH]3	6
Cc3nc(c2cccc(c1nc(C)n(C)n1)n2)nn3C	6
c3ccc(c2ccn(c1ccccn1)n2)nc3	6
S=C=Nc2c(N=C=S)c(c1ccccn1)[nH]c2c3ccccn3	6
c3ccc(c2csc(c1ccccn1)n2)nc3	6
c3ccc(Nc2nc(c1ccccn1)cs2)nc3	6
c3ccc(c2csc(Nc1cnccn1)n2)nc3	6
c3ccc(Cn2ccc(c1ccccn1)n2)nc3	6
c3ccc(c2cccc(c1nccs1)n2)nc3	6
Cc3csc(c2cccc(c1ccccn1)n2)n3	6
c1cnc4c(c1)ccc3ccc(c2nccs2)nc34	6
c5ccc4sc(c3ccc2ccc1cccnc1c2n3)nc4c5	6
c1cnc4c(c1)ccc3ccc(C2=NCCS2)nc34	6
c5ccc(c4csc(c3ccc2ccc1cccnc1c2n3)n4)nc5	6
c5ccc4[nH]c(c3ccc2ccc1cccnc1c2n3)nc4c5	6
c1cnc4c(c1)ccc3ccc(C2=NCCN2)nc34	6
c3ccc(c2cccc(n1cccn1)n2)nc3	6
Cc3cc(C)n(c2cccc(c1ccccn1)n2)n3	6
Cc4cc(C)n(c3ccc2ccc1cccnc1c2n3)n4	6
c3ccc(c2cccc(Cn1cccn1)n2)nc3	6
Cc3cc(C)n(Cc2cccc(c1ccccn1)n2)n3	6
c3ccc(c2cccc(c1nc[nH]n1)n2)nc3	6
Cn3cnc(c2cccc(c1ccccn1)n2)n3	6
Cc3nc(c2cccc(c1ccccn1)n2)n[nH]3	6
Cc3nc(c2cccc(c1ccccn1)n2)nn3C	6

c1cnc4c(c1)ccc3ccc(c2nc[nH]n2)nc34	6
Cn4cnc(c3ccc2ccc1cccnc1c2n3)n4	6
Cc4nc(c3ccc2ccc1cccnc1c2n3)n[nH]4	6
Cc4nc(c3ccc2ccc1cccnc1c2n3)nn4C	6
Cc4nc(c3ccc2ccc1cccnc1c2n3)no4	6
c3c[nH]c(Cc2nc(Cc1c[nH]cn1)c[nH]2)n3	6
C(=N¥Nc1ccccc1)/c3cccc(c2ccccn2)n3	6
C(=N¥Nc1ccc[nH]1)/c3cccc(c2ccccn2)n3	6
CN/N=C/c3ccc2ccc1cccnc1c2n3	6
C(=N¥Nc1ccccc1)/c4ccc3ccc2cccnc2c3n4	6
C(=N¥Nc1ccc[nH]1)/c4ccc3ccc2cccnc2c3n4	6
C(=N¥Nc1ccccc1)/c5ccc4ccc3ccc(/C=N/Nc2ccccc2)nc3c4n5	6
CC(C)(C)/N=C/c3ccc2ccc1cccnc1c2n3	6
COc2cc(/C=N/O)nc(c1ccccn1)c2	6
NC(=O)c2cccc(c1ccccn1)n2	6
NC(=S)c2cccc(c1ccccn1)n2	6
C/N=C/c1cccc(/C=N/C)n1	6
C(=N¥Cc1ccccc1)/c3cccc(/C=N/Cc2ccccc2)n3	6
O/N=C/c1cccc(/C=N/O)n1	6
C/N=C(C)/c1cccc(/C(C)=N/C)n1	6
CCCC/N=C(C)/c1cccc(/C(C)=N/CCCC)n1	6
COCC/N=C(C)/c1cccc(/C(C)=N/CCOC)n1	6
C/C(=N¥c1ccccc1)c3cccc(/C(C)=N/c2ccccc2)n3	6
C/C(=N¥c1ccccc1F)c3cccc(/C(C)=N/c2ccccc2F)n3	6
C/C(=N¥c1ccc(C)c(C)c1)c3cccc(/C(C)=N/c2ccc(C)c(C)c2)n3	6
C/C(=N¥Cc1ccccc1)c3cccc(/C(C)=N/Cc2ccccc2)n3	6
COc3ccc(C/N=C(C)/c2cccc(/C(C)=N/Cc1ccc(OC)cc1)n2)cc3	6
C/C(=N¥Cc1cccs1)c3cccc(/C(C)=N/Cc2cccs2)n3	6
C/C(=N¥Cc1ccncc1)c3cccc(/C(C)=N/Cc2ccncc2)n3	6
NCc1cccc(CN)n1	6
c2ccc(CNCc1ccccn1)nc2	6
CN(Cc1ccccn1)Cc2ccccn2	6
c3ccc(N(Cc1ccccn1)Cc2ccccn2)cc3	6
C(=N¥Cc1ccccn1)/c2ccccn2	6
NCC/N=C/c1ccccn1	6

NCCC/N=C/c1ccccn1	6
O=C(NC(=O)c1ccccn1)c2ccccn2	6
Cn1ccnc1CNCc2nccn2C	6
CCN(Cn1cccn1)Cn2cccn2	6
c4ccc3[nH]c(CNCc2nc1ccccc1[nH]2)nc3c4	6
CN(Cc2nc1ccccc1[nH]2)Cc4nc3ccccc3[nH]4	6
C1CNCCNCCN1	6
CN1CCN(C)CCN(C)CC1	6
CC(C)N1CCN(C(C)C)CCN(C(C)C)CC1	6
CC(C)(C)N1CCN(C(C)(C)C)CCN(C(C)(C)C)CC1	6
c3ccc(CN(Cc1ccccn1)Cc2ccccn2)nc3	6
CC(c1ccccn1)N(Cc2ccccn2)Cc3ccccn3	6
Cc3cccc(CN(Cc1ccccn1)Cc2ccccn2)n3	6
c4ccc(c3cccc(CN(Cc1ccccn1)Cc2ccccn2)n3)cc4	6
c3ccc(CCN(Cc1ccccn1)Cc2ccccn2)nc3	6
Cc3ccnc(CN(Cc1cc(C)ccn1)Cc2cc(C)ccn2)c3	6
Cc1cccnc1CN(Cc2ncccc2C)Cc3ncccc3C	6
Cc3cccc(CN(Cc1ccccn1)Cc2cccc(C)n2)n3	6
Cc3cccc(CN(Cc1cccc(C)n1)Cc2cccc(C)n2)n3	6
c4ccc(CN(Cc1ccccn1)Cc3ccc2ccccc2n3)nc4	6
c5ccc(CN(Cc2ccc1ccccc1n2)Cc4ccc3ccccc3n4)nc5	6
NCCN(Cc1ccccn1)Cc2ccccn2	6
CN(C)CCN(Cc1ccccn1)Cc2ccccn2	6
NCCCN(Cc1ccccn1)Cc2ccccn2	6
CN(C)CCN(CCN(C)C)Cc1ccccn1	6
NCCCN(CCCN)Cc1ccccn1	6
Cc3cc(C)n(CN(Cn1nc(C)cc1C)Cn2nc(C)cc2C)n3	6
c2ccc(CNCCNCc1ccccn1)nc2	6
CN(CCN(C)Cc1ccccn1)Cc2ccccn2	6
c3ccc(CN2CCCN(Cc1ccccn1)CC2)nc3	6
COc4cc(CN(CCN(Cc1cc(OC)c(OC)c(OC)c1)Cc2ccccn2)Cc3ccccn3)cc(OC)c4OC	6
Cc2cccc(CNCCNCc1ccccn1)n2	6
Cc2cccc(CNCCNCc1cccc(C)n1)n2	6
Cc2cccc(CN(C)CCN(C)Cc1cccc(C)n1)n2	6
CN(Cc1ccccn1)C2CCCCC2N(C)Cc3ccccn3	6

Cc3cccc(CN(C)C1CCCCC1N(C)Cc2cccc(C)n2)n3	6
CCNCc1ccccn1.CCNCc1ccccn1	6
COC(=O)C13CN(C)CC(C(=O)OC)(C1=O)C(Cc2ccccn2)N(C)C3Cc4ccccn4	6
COC(=O)C13CN(C)CC(C(=O)OC)(C1=O)C(Cc2cccc(C)n2)N(C)C3Cc4cccc(C)n4	6
C(=N¥CCN1CCNC1c2ccccn2)/c3ccccn3	6
c3ccc(c2cc(c1ccccn1)[nH]n2)nc3	6
C/C(C/C(C)=N/Cc1ccccn1)=N¥Cc2ccccn2	6
Cc2cc(C)n(CN(C)CCN(C)Cn1nc(C)cc1C)n2	6
C1CNCCNCCCNCCNC1	6
C/C1=N¥CCNC(C)(C)C/C(C)=N/CCNC(C)(C)C1	6
C1CNCCNCCNCCNC1	6
C1CNCCCNCCNCCCNC1	6
C1CNCCCNCCCNCCCNC1	6
CN1CCCN2CCCN(CCCN(C)CC1)CC2	6
CCCCCCCCCCCCCCCCOc5cc(c2nc1ccccc1n2CCCCCCCCCCCCCCCC)nc(c4nc3ccccc3 n4CCCCCCCCCCCCCCCC)c5	6
Cn5c(c3cccc(c2nc1ccccc1[nH]2)n3)nc4ccccc45	6
CCn5c(c3cccc(c2nc1ccccc1[nH]2)n3)nc4ccccc45	6
Oc1ccccc1c5cc(c4cccc(c3cc(c2ccccc2O)[nH]n3)n4)n[nH]5	6
COc1ccccc1c5cc(c4cccc(c3cc(c2ccccc2OC)[nH]n3)n4)n[nH]5	6
Cc5ccc4ccc3ccc(c2nc1ccccc1[nH]2)nc3c4n5	6
c4ccc(c3ccn(c2cccc(c1ccccn1)n2)n3)cc4	6
c4ccc(c3cccc(n2ccc(c1ccccn1)n2)n3)nc4	6
c1cnc4c(c1)ccc3ccc(c2nn[nH]n2)nc34	6
Cn4nnc(c3ccc2ccc1cccnc1c2n3)n4	6
CCn4nnc(c3ccc2ccc1cccnc1c2n3)n4	6
CCCCn4nnc(c3ccc2ccc1cccnc1c2n3)n4	6
CCCCCn4nnc(c3ccc2ccc1cccnc1c2n3)n4	6
CCCCCCCCCCCCCCCCn4nnc(c3ccc2ccc1cccnc1c2n3)n4	6
CC3(C)COC(c2cccc(C1=NC(C)(C)CO1)n2)=N3	6
C/C(=N¥c1c(F)cccc1F)c3cccc(/C(C)=N/c2c(F)cccc2F)n3	6
c5ccc(CC4COC(c3cccc(C2=NC(Cc1ccccc1)CO2)n3)=N4)cc5	6
c7cc(c3cc(c2ccc1ccccc1c2)[nH]n3)nc(c6cc(c5ccc4ccccc4c5)[nH]n6)c7	6
c5ccc(c4cc(c3cccc(C2Nc1ccccc1N2)n3)n[nH]4)cc5	6
C/C(=N¥c1ccc(F)cc1F)c3cccc(/C(C)=N/c2ccc(F)cc2F)n3	6

c3cc(C1=NCCO1)nc(C2=NCCO2)c3	6
C/C(=N¥N)c1cccc(/C(C)=N/N)n1	6
Cc3cc(c2cccc(c1cc(C)[nH]n1)n2)n[nH]3	6
COc3ccc(/N=C(C)¥c2cccc(/C(C)=N/c1ccc(OC)cc1)n2)cc3	6
Cc5ccc(c4cc(c3cccc(C2Nc1ccccc1N2)n3)n[nH]4)cc5	6
Cc5cc(C)c(c4cc(c3cccc(C2Nc1ccccc1N2)n3)n[nH]4)c(C)c5	6
Cc5c(C)c(C)c(c4cc(c3cccc(C2Nc1ccccc1N2)n3)n[nH]4)c(C)c5C	6
C/C(=N/C1CCCCC1)c3cccc(/C(C)=N/C2CCCCC2)n3	6
C/C2=N/CCCOCCOCC/N=C(C)¥c1cccc(n1)/C(C)=N¥CCOCCOCC/N=C(C)¥c3cccc2n3	6
C/C(=N/c3cc(c1ccc(F)cc1)c(C)c(c2ccc(F)cc2)c3)c7cccc(/C(C)=N/c6cc(c4ccc(F)cc4)c(C)c(c5ccc(F)cc5)c6)n7	6
Clc3cc(C1=NCCO1)nc(C2=NCCO2)c3	6
C/C(=N/O)c1cccc(/C(C)=N/O)n1	6
C/C(=N¥c1c(F)cccc1F)c4cccc(C3Nc2ccccc2N3C)n4	6
C/C(=N¥C(C)c1ccccc1)c3cccc(/C(C)=N/C(C)c2ccccc2)n3	6
C/C(=N¥c1ccc(N(=O)=O)cc1)c3cccc(/C(C)=N/c2ccc(N(=O)=O)cc2)n3	6
C=CCn3ccc(c2cccc(c1ccn(CC=C)n1)n2)n3	6
c5ccc(Cn4ccc(c3cccc(c2ccn(Cc1ccccc1)n2)n3)n4)cc5	6
CC(C)n3ccc(c2cccc(c1ccn(C(C)C)n1)n2)n3	6
Cn3ccc(c2cccc(c1ccn(C)n1)n2)n3	6
C/C(=N¥c1ccc(C(C)(C)C)cc1)c3cccc(/C(C)=N/c2ccc(C(C)(C)C)cc2)n3	6
CCc1cccc(CC)c1c5[nH]nc(c4cccc(c3n[nH]c(c2c(CC)cccc2CC)c3O)n4)c5O	6
Oc4c(c3cccc(c2n[nH]c(c1c(F)cccc1F)c2O)n3)n[nH]c4c5c(F)cccc5F	6
CC3(C)CSC(c2cccc(C1=NC(C)(C)CS1)n2)=N3	6
c3cc(C1=NCCS1)nc(C2=NCCS2)c3	6
C/C1=N¥CCNCCCNCC/N=C(C)/c2cccc1n2	6
C/C1=N¥CCCNCCNCC/N=C(C)/c2cccc1n2	6
C/C(=N/CCN)c1ccccn1	6
CNCC/N=C(C)¥c1ccccn1	6
c4ccc(CN(Cc1ccccn1)C(c2ccccn2)c3ccccn3)nc4	6
COc4cccc(CN(Cc1cccc(OC)n1)C(c2ccccn2)c3ccccn3)n4	6
Brc4cccc(CN(Cc1cccc(Br)n1)C(c2ccccn2)c3ccccn3)n4	6
COC(=O)c4cccc(CN(Cc1cccc(C(=O)OC)n1)C(c2ccccn2)c3ccccn3)n4	6
OC(c1ccccn1)(c2ccccn2)c5cccc(C(O)(c3ccccn3)c4ccccn4)n5	6
COC(c1ccccn1)(c2ccccn2)c5cccc(C(OC)(c3ccccn3)c4ccccn4)n5	6

CN(CCN(Cc1ccccn1)Cc2ccccn2)Cc3ccccn3	6
c4ccc(CN(CCN(Cc1ccccn1)Cc2ccccn2)Cc3ccccn3)cc4	6
C(=N¥CCN(Cc1ccccn1)Cc2ccccn2)/c3ccccn3	6
C(=N¥c1ccccc1N(Cc2ccccn2)Cc3ccccn3)/c4ccccn4	6
CC(=N)C(/C(C)=N/Cc1ccccn1)¥C(C)=N¥Cc2ccccn2	6
C/C(=N¥Cc1ccccn1)C(/C(C)=N/Cc2ccccn2)C(=N)c3ccccc3	6
C(=N¥CCCNCCC/N=C/c1ccccn1)/c2ccccn2	6
CN1CCCN2Cc4cccc(CN(CCC1)Cc3cccc(C2)n3)n4	6
CN3CCN(C)CCN(C(c1ccccn1)c2ccccn2)CC3	6
c3ccc(CN2CCNCCN(Cc1ccccn1)CC2)nc3	6
c6ccc(CN5CCN(CCN3CCN(Cc1ccccn1)CCN(Cc2ccccn2)CC3)CCN(Cc4ccccn4)CC5)nc6	6
c5ccc(c4cccc(c3cccc(c2cccc(c1ccccn1)n2)n3)n4)nc5	6
c7ccc(c6cc(c1ccccn1)nc(c5cccc(c4cc(c2ccccc2)cc(c3ccccn3)n4)n5)c6)cc7	6
COC(=O)C13CNCC(C(=O)OC)(C1=O)C(Cc2ccccn2)N(C)C3Cc4ccccn4	6
COC(=O)C14CN(C)CC(C(=O)OC)(C1=O)C(Cc2ccccn2)N(Cc3ccccn3)C4Cc5ccccn5	6
COC(=O)C14CN(CCO)CC(C(=O)OC)(C1=O)C(Cc2ccccn2)N(Cc3ccccn3)C4Cc5ccccn5	6
C/C1=N¥CCOCCOCC/N=C(C)/c2cccc1n2	6
C/C1=N¥CCNCCNCC/N=C(C)/c2cccc1n2	6
COC5c6cccc(/C=N/c1ccccc1/N=C/c2cccc(n2)C4Nc3ccccc3N45)n6	6
c4ccc(CN(CCN(Cc1ccccn1)Cc2ccccn2)Cc3ccccn3)nc4	6
Cc4cccc(CN(CCN(Cc1ccccn1)Cc2cccc(C)n2)Cc3ccccn3)n4	6
c5ccc(C[N@@](Cc1ccccn1)C2CCCCC2N(Cc3ccccn3)Cc4ccccn4)nc5	6
CC(CN(Cc1ccccn1)Cc2ccccn2)N(Cc3ccccn3)Cc4ccccn4	6
CC(C(C)N(Cc1ccccn1)Cc2ccccn2)N(Cc3ccccn3)Cc4ccccn4	6
c4ccc(CN(CCCN(Cc1ccccn1)Cc2ccccn2)Cc3ccccn3)nc4	6
Cc4cccc(CN(CCN(Cc1ccccn1)Cc2ccccn2)Cc3ccccn3)n4	6
Cc4cccc(CN(CCN(Cc1ccccn1)Cc2ccccn2)Cc3cccc(C)n3)n4	6
Cc4ccc(CN(CCN(Cc1ccccn1)Cc2ccc(C)cn2)Cc3ccccn3)nc4	6
Cc1cccnc1CN(CCN(Cc2ccccn2)Cc3ncccc3C)Cc4ccccn4	6
Cc4ccnc(CN(CCN(Cc1cc(C)ccn1)Cc2cc(C)ccn2)Cc3cc(C)ccn3)c4	6
COc4ccnc(CN(CCN(Cc1cc(OC)ccn1)Cc2cc(OC)ccn2)Cc3cc(OC)ccn3)c4	6
Clc4ccnc(CN(CCN(Cc1cc(Cl)ccn1)Cc2cc(Cl)ccn2)Cc3cc(Cl)ccn3)c4	6
O=N(=O)c4ccnc(CN(CCN(Cc1cc(N(=O)=O)ccn1)Cc2cc(N(=O)=O)ccn2)Cc3cc(N(=O)=O)ccn3)c4	6
c4cnn(CN(CCN(Cn1cccn1)Cn2cccn2)Cn3cccn3)c4	6

c3ccc(CNCCCN(CCCNCc1ccccn1)Cc2ccccn2)nc3	6
CN(Cc1ccccn1)CC(CN(C)Cc2ccccn2)CN(C)Cc3ccccn3	6
COC(c1ccccn1)N(CCNCc2ccccn2)CCNCc3ccccn3	6
CCOC(c1ccccn1)N(CCNCc2ccccn2)CCNCc3ccccn3	6
CCCCOC(c1ccccn1)N(CCNCc2ccccn2)CCNCc3ccccn3	6
CN(CCN(CCN(C)Cc1ccccn1)Cc2ccccn2)Cc3ccccn3	6
COC(=O)C25CN(Cc1ccccn1)CC(C(=O)OC)(C2=O)C(Cc3ccccn3)N(Cc4ccccn4)C5Cc6ccccn6	6
Cc2nc(/C=N/CCCNCCNCCC/N=C/c1c[nH]c(C)n1)c[nH]2	6
c6ccc(CN(Cc1ccccn1)Cc5cccc(c4cccc(CN(Cc2ccccc2)Cc3ccccn3)n4)n5)cc6	6
c4ccc(CN3CCN(Cc1ccccn1)CCN(Cc2ccccn2)CC3)nc4	6
Cc4cccc(CN3CCN(Cc1ccccn1)CCN(Cc2ccccn2)CC3)n4	6
c4cnn(CN3CCN(Cn1cccn1)CCN(Cn2cccn2)CC3)c4	6
c4cc(CN3CCN(Cc1cc[nH]n1)CCN(Cc2cc[nH]n2)CC3)n[nH]4	6
Cn1ccnc1CN4CCN(Cc2nccn2C)CCN(Cc3nccn3C)CC4	6
c4ccc(CN3CCCN(Cc1ccccn1)CCN(Cc2ccccn2)CC3)nc4	6
c4ccc(CN3CCCN(Cc1ccccn1)CCCN(Cc2ccccn2)CCC3)nc4	6
C1CNCC2CNCCNCC(CN1)CNCCNC2	6
NC12CNCCNCC(N)(CNCCNC1)CNCCNC2	6
[NH3+]C12CNCCNCC([NH3+])(CNCCNC1)CNCCNC2	6
C1CNCCNCCNCCNCCNCCN1	6
Cc3cccc(/C=N/CCN(CC/N=C/c1ccccn1)CC/N=C/c2ccccn2)n3	6
Cc1nc(/C=N/CCN(CC/N=C/c2cccc(C)n2)CC/N=C/c3nc(C)ccc3)ccc1	6
c3ccc(CNCCN(CCNCc1ccccn1)CCNCc2ccccn2)nc3	6
Cc3ncc(/C=N/CCN(CC/N=C/c1cnc(C)[nH]1)CC/N=C/c2cnc(C)[nH]2)[nH]3	6
C(=N¥CCN(CC/N=C/c2cnc(c1ccccc1)[nH]2)CC/N=C/c4cnc(c3ccccc3)[nH]4)/c6cnc(c5ccccc5)[nH]6	6
c6ccc(CN(Cc1ccccn1)Cc5cccc(c4cccc(CN(Cc2ccccn2)Cc3ccccn3)n4)n5)nc6	6
c5ccc(CN(Cc1ccccn1)Cc4cccc(CN(Cc2ccccn2)Cc3ccccn3)n4)nc5	6
CN4CCN(Cc1ccccn1)CCN(Cc2ccccn2)CCN(Cc3ccccn3)CC4	6
c5ccc(CN4CCN(Cc1ccccn1)CCN(Cc2ccccn2)CCN(Cc3ccccn3)CC4)nc5	6
C1CNCCN2CCNCCNCCN(CCN1)CNCCNC2	6
[CH3]C#N	6
[C-]#N	6
Cc3cccc(CN(CCc1ccccn1)Cc2cccc(C)n2)n3	6

Cc2cccc(CN(CCN(C)C)Cc1cccc(C)n1)n2	6
CN(CCN(C)Cc2ccc1ccccc1n2)Cc4ccc3ccccc3n4	6
c4ccc(CN(CCN(Cc1ccccc1)Cc2ccccn2)Cc3ccccn3)cc4	6
Clc2ccnc(CNCCNCc1cc(Cl)ccn1)c2	6
c3ccc(CNC1CCCCC1NCc2ccccn2)nc3	6
c3ccc(CN2CCCNCCN(Cc1ccccn1)CCCNCC2)nc3	6
c4ccc(CN3CCCN2CCN(CCCN(Cc1ccccn1)CC2)CC3)nc4	6
C/C(=N¥Cc1ccccn1)C(C(=N)Cc2ccccc2)/C(C)=N/Cc3ccccn3	6
C/C1=N/CCOCCCOCC/N=C(C)¥c2cccc1n2	6
O=C([O-])c1ccccc1	6
[N-]=[N+]=[N-]	6

Figure S1. Workflow for creating FeN_6–SSD. Each ligand is represented by its SMILES notation and the reference number(s) of the review article(s) from which it was extracted. Ligands that could not be expressed in SMILES (e.g., ferrocene) were excluded. Crystal structures were queried in the CSD using the SMILES together with Fe through the CSD Python API; when no hit was obtained, the ligand was searched by drawing it in ConQuest.

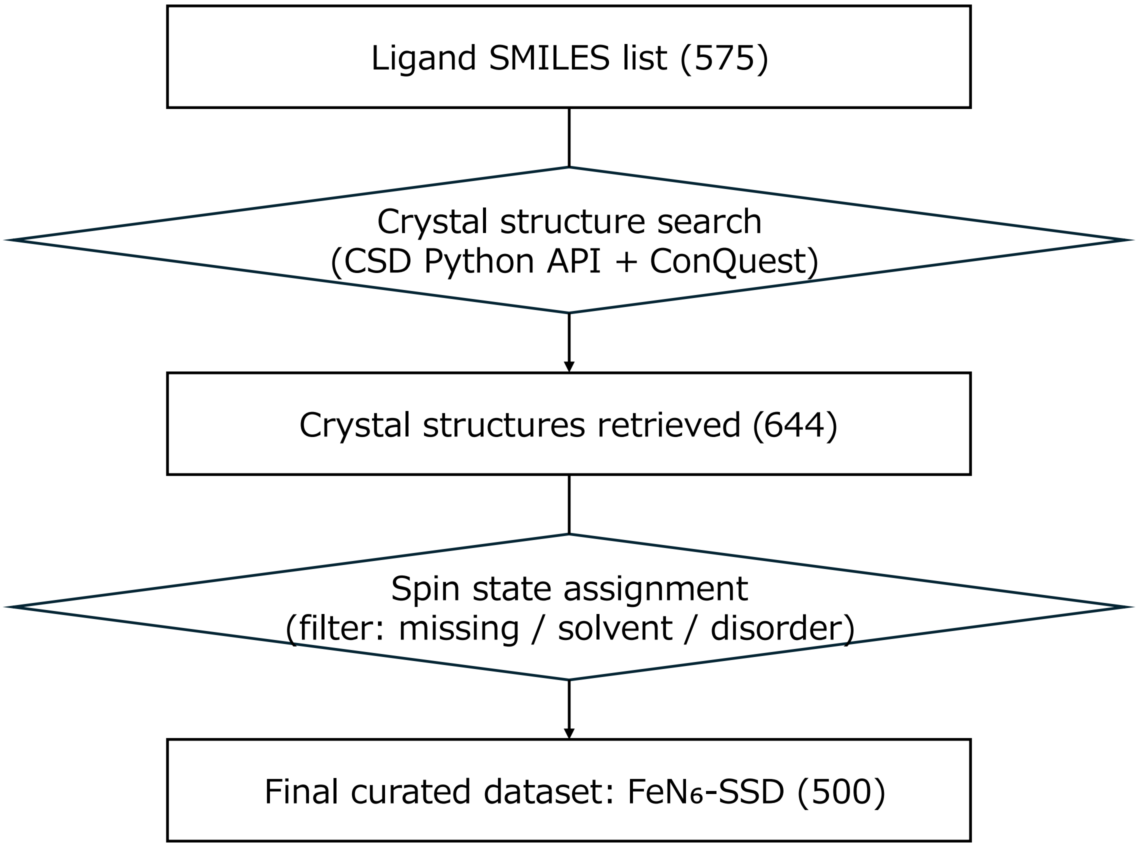

Table S2. Components of FeN_6–SSD (500 entries). The dataset consists of Fe(II) complexes with a FeN_6 coordination environment. The table lists the CSD refcode, assigned spin state (LS, HS, or SCO), CIF-reported spin state, temperature information of experimental measurements reported in the literature (e.g., magnetic susceptibility ranges, Mössbauer measurement points, or EPR measurement ranges), and reference numbers. Entries whose CIF spin state is annotated as "MIX." (indicating crystal structures in which HS and LS molecules coexist within the unit cell) were not used for model construction in this study.

License: FeN_6–SSD is distributed under the Creative Commons Attribution 4.0 International (CC BY 4.0) license (https://creativecommons.org/licenses/by/4.0/). Users are free to share and adapt the dataset, provided appropriate credit is given. The original crystallographic data remain the property of the CCDC and FIZ Karlsruhe and are subject to their respective Terms of Use.

CSD	Spin State	Spin state (CIF file)	Magnetic susceptibility Temp. range	Mössbauer temp. range	EPR Temp. range	Ref.
GAPSIA	HS	HS	5-300K			7
ADIYUH	HS	HS	10-330K			8
GAPSOG	HS	HS	5-300K			7
OZOGIV	HS	HS	5-300K			9
ECELAA	HS	HS	5-300K			10
ECELEE01	SCO	MIX.	5–300 K (from figure axis)			9
ECELII	HS	HS	5-300K			10
GAPTAT	HS	HS	5-300K			7
GAPTEX	HS	HS	5-300K			7
GAPSUM	HS	HS	5-300K			7

GAPSUM01	HS	HS	5-300K			7
GAPTIB	HS	HS	5-300K			7
FOYYAU	HS	HS	4-350K			11
DEDNOQ	HS	HS	5-300K<br>(from figure axis)			12
XENBEX06	SCO	HS	5-300K			13
XENBEX07	SCO	LS	5-300K			13
ADIYOB	SCO	LS	10-330K			8
LUXMOH	SCO	HS	5-270K			14
LUXMOH01	SCO	LS	5-270K			14
AFANEB02	SCO	HS	5-300 K<br>(from figure axis)			15
AFANEB	SCO	LS	5-300 K<br>(from figure axis)			15
AFANIF02	SCO	HS	5-250K			15
NESVUD	SCO	HS	10-290K<br>(from figure axis)			16
NESVUD01	SCO	LS	10-290K<br>(from figure axis)			16
REWPUF	SCO	HS	5-300K<br>(from figure axis)			17
REWPUF02	SCO	LS	5-300K<br>(from figure axis)			17
REWPOZ	SCO	HS	5-300K<br>(from figure axis)			17

IYUFOX01	SCO	HS	5-300K (from figure axis)			18
IYUFOX	SCO	LS	5-300K (from figure axis)			18
NESVOX	SCO	HS	10-290K (from figure axis)			16
NESVOX02	SCO	LS	10-290K (from figure axis)			16
FOYYAU06	SCO	HS	4-350K			11
FOYYAU02	SCO	LS	4-350K			11
COMRAY	SCO	LS	5-300K (from figure axis)			19
XOCFIF	SCO	LS	4.5-380K			20
COMREC	SCO	LS	5-300K (from figure axis)			19
YUZTOD	SCO	LS	5-480K			21
YUZTIX	SCO	LS	5-480K			21
JAFCEZ	SCO	HS	5-300K			22
JAFCEZ01	SCO	LS	5-300K			22
JAFBOI01	SCO	HS	5-300K (from figure axis)			23
JAFBOI03	SCO	LS	5-300K (from figure axis)			23
JAFBUO01	SCO	HS	5-300K (from figure axis)			23

JAFBUO03	SCO	LS	5-300K (from figure axis)			23
IGEDUU	LS	LS	5-360K (from figure axis)			24
RESWES	HS	HS	5-300K (from figure axis)			25
OBUJAW	HS	HS		4K,293K		26
CERDEJ01	HS	HS	5-300K (from figure axis)			27
MALJIT	HS	HS	5-300K (from figure axis)			28
HMPBFE	SCO	HS	50-300K			29
FIWXIT	SCO	HS	5-300K (from figure axis)			30
FIWXEP	SCO	HS	5-300K (from figure axis)			30
RESWIW	SCO	HS	5-300K (from figure axis)			25
RESWIW01	SCO	MIX.	5-300K (from figure axis)			25
MALJIT01	SCO	HS		4.2K,78K, 155K,225K, 295K		28
MALJIT03	SCO	LS		4.2K,78K, 155K,225K, 295K		28

MALJOZ	SCO	HS		4.2K,78K, 295K		28
MALJOZ01	SCO	LS		4.2K,78K, 295K		28
FIWXOZ02	SCO	HS	4-350 K (up to 350 K depending on compound)			30
FIWXOZ	SCO	LS	4-350 K (up to 350 K depending on compound)			30
HPZBFE	SCO	LS	SCO at 393 K (reported in Hutchinson et al., Chem. Commun., in press)			31
HOQFID	SCO	LS	300-460K			32
MALJEP	LS	LS		78K,293K		28
MOFREE	SCO	HS	5-325K			33
MOFRAA	SCO	HS	5-325K			33
VUJRIC	SCO	LS	7-350K	100K,255K, 265K,300K		34
YUJBUB01	SCO	MIX.	5-300K (from figure axis)			35
YUJBUB	SCO	LS	5-300K (from figure axis)			35

YIMYEZ	SCO	LS	4-370K (from figure axis)			36
YIMYEZ02	SCO	HS	4-295K (from figure axis)			36
YIMYEZ01	SCO	LS	4-295K (from figure axis)			36
YIMYID04	SCO	HS	4-295K (from figure axis)			36
YIMYID02	SCO	LS	4-295K (from figure axis)			36
YIMYOJ	SCO	LS	40-350K (from figure axis)			36
XAMNII	SCO	LS	4-350K			37
XAMNII01	SCO	LS	4-350K			37
XEVYUS	SCO	LS	5-300K (from figure axis)			38
JIQDAP	HS	HS	5-300K			39
ISULAJ	HS	HS	5-300K			39,40
GAZQUT	HS	HS	15-300K			41
QOSVOJ	HS	HS	4-300K			42
ULODUV	HS	HS	5-300K			43
ULOFAD	HS	HS	5-300K			43
ULOFEH	HS	HS	5-300K			43
NIQFUP11	SCO	HS	5-300K (from figure axis)			44

NIQFUP	SCO	LS	5-300K<br>(from figure axis)			44
HIXFUQ01	SCO	HS	10-400K			45
HIXFUQ	SCO	LS	10-400K			45
JIQDET	SCO	HS	5-300K			39
JIQDET04	SCO	LS	5-300K			39
EBUQAU01	SCO	HS	5-300K			46
EBUQAU	SCO	LS	5-300K			46
NAVYEP	SCO	HS	5-300K			47
YELWOC	SCO	HS	5-300K			48
YELWOC01	SCO	MIX.	5-300K			48
YELWUI	SCO	HS	5-300K			48
YELWUI02	SCO	LS	5-300K			48
YELXET	SCO	HS	5-300K			48
YELXET01	SCO	LS	5-300K			48
YELXAP	SCO	HS	5-300K			48
ULODIJ	SCO	HS	5-300K			43
ULODIJ01	SCO	MIX.	5-300K			43
PASDOD01	SCO	HS		77K,295K		49
ENUBAR	SCO	HS		77K,295K		50
ENUBAR02	SCO	LS		77K,295K		50
EYIJEB	SCO	LS		77K,298K		51
HIXFOK	LS	LS	10-400K			45

FETPYR	HS	HS	77K, 294.5K (2 points)			52
WIGBAP	HS	HS	25-300K (from figure axis)			53
WIFZUG	SCO	HS	75-300K (from figure axis)			53
ULAHIY	HS	HS	5-300K (from figure axis)			54
QAHPIY02	SCO	MIX.	5-300K			55
QAHPIY01	SCO	MIX.	5-300K			55
FEISXC01	SCO	HS	5-300K			55
JUVHIR01	SCO	HS		~5K,~160K, RT		56
JUVHIR03	SCO	MIX.	5-300K (from figure axis)			57
JUVHEN	SCO	MIX.		~5K,~160K, RT		56
LAGJEK01	SCO	MIX.	5-300K (from figure axis)			58
LAGJEK02	SCO	MIX.	5-300K (from figure axis)			58
TUNBIN	SCO	MIX.	10-295K (from figure axis)			59
RAKZIM	SCO	HS	5-300K (from figure axis)			60
RAKZIM01	SCO	LS	5-300K (from figure axis)			60,61

PEJQIF	SCO	HS	30-300K			62,63
PEJQIF01	SCO	LS	30-300K			62,63
YAGYIP01	SCO	HS	5-250K<br>(from figure axis)			64
YAGYIP	SCO	LS	5-250K<br>(from figure axis)			64
YAGYUB01	SCO	HS	120-300K<br>(from figure axis)			64
YAGYUB	SCO	LS	120-300K<br>(from figure axis)			64
JANSAS	SCO	HS			77-295K	65
JANSAS06	SCO	LS			77-295K	65
JUVGUC	SCO	HS		~5K,~160K,<br>RT		56
NEFVID	SCO	HS	20-180K<br>(from figure axis)			66
YEQKIR	SCO	HS	5-300K			67
APAFEH	LS	LS	LS throughout<br>(no SCO;<br>reported in text)			68
APAFEM01	SCO	HS	5-300K			68,69
APAFEM05	SCO	LS	5-300K			68,69
FEPICC36	SCO	HS	2-298K			70
FEPICC37	SCO	LS	2-298K			70

IBIXEX	SCO	HS	2-30K and 230-300K			71
IBIWUM	HS	HS	2-30K and 230-300K			71
IBIXAT10	SCO	HS	50-250K (from figure axis)			72
IBIXAT14	SCO	LS	50-250K (from figure axis)			72
IBIXIB	HS	HS	2-30K and 230-300K			71
DIXLUR10	SCO	HS	5-298K (from figure axis)	125.5K, 280.0K		73
PUTGOC01	SCO	HS	2-400K			74
PUTGOC	SCO	LS	2-400K			74
PUTHAP01	SCO	HS	2-400K			74
PUTHAP	SCO	LS	2-400K			74
PUTHIX01	SCO	HS	2-400K			74
PUTHIX	SCO	LS	2-400K			74
AKENAF	SCO	HS	100-400K			75
AKENAF01	SCO	LS	100-400K			75
AKEMUY	SCO	LS	100-400K			75
RONPIT41	SCO	HS	5-300K (from figure axis)			76

RONPIT39	SCO	LS	5-300K (from figure axis)			76
RONPIT26	SCO	HS	5-300K (from figure axis)			76
RONPIT34	SCO	LS	5-300K (from figure axis)			76
NOWBIK01	SCO	HS	80-300K (from figure axis)			77
NOWBIK	SCO	LS	80-300K (from figure axis)			77
WATJEJ	SCO	MIX.	50-400K (from figure axis)			78
WATJEJ01	SCO	LS	50-400K (from figure axis)			78
XECNAU01	SCO	HS	50-300K (from figure axis)			79
XECNAU	SCO	LS	50-300K (from figure axis)			79
XECMIB01	SCO	HS	5-300K (from figure axis)			79
XECMIB02	SCO	LS	5-300K (from figure axis)			79
COMQUR	SCO	HS	5-300K (from figure axis)			80
COMQUR01	SCO	LS	5-300K (from figure axis)			80

UFIPIJ01	SCO	HS	5-300K<br>(from figure axis)			81
UFIPIJ	SCO	LS	5-300K<br>(from figure axis)			81
VEWVEY	SCO	HS		77K,298K		82
CATWUS	HS	HS	5-300K<br>(from figure axis)			83
KABYER	SCO	MIX.	89-323K			84
SIXJUE	LS	LS	303-323K	77K		85
QALMAR	SCO	HS	4-325K	4.2K,80K,<br>150K,205K		86
QALMAR01	SCO	MIX.	4-325K			86
YIVSEB	SCO	HS	89-303K	77K,298K		87
OJIVOS	LS	LS	2-300K			88
ABIWAK	SCO	LS	5-340K			89
DEVDUD	SCO	LS	111-333K			90
ZIMMAJ	HS	HS	2-300K	2K,20K,50K,<br>100K,300K		91
VEYTIC	HS	HS	2K,25K,45K,<br>100K,300K<br>(5 points)			92
VEYTEY	HS	HS	2K,25K,45K,<br>100K,300K<br>(5 points)			92
NICSEA	LS	LS	5-400K<br>(from figure axis)			93

POGXOA	HS	HS	1.9-300K			94
POGXUG	HS	HS	1.9-300K			94
OLAYAC	SCO	HS	5-300K			95
OLAYAC01	SCO	HS	5-300K			95
IMAHIO01	SCO	HS	5-300K (from figure axis)			96
IMAHIO	SCO	LS	5-300K (from figure axis)			96
FEBPYC04	SCO	HS	77-293K			97,98
FEBPYC02	SCO	LS	77-293K			97,98
KEKVIF	SCO	HS	130-290K			99
KEKVIF01	SCO	LS	130-290K			99
PAGXAW	HS	HS	6K-RT	4K,2K, 78K,300K		100
POHLON	SCO	HS	4.5-295K			101
POHLON01	SCO	LS	4.5-295K			101
POWSID	SCO	HS	86-275K			102
LAYREJ01	HS	HS	4-300K			103
LAYQUY	HS	HS	4-300K			103
LAYRAF	HS	HS	4-300K			103
LOQPAI	HS	HS	4-200K			104
QAHVIE	SCO	HS	120-315K			105
QAHVOK	SCO	HS	120-315K			105
ZUCROE01	SCO	MIX.	7-460K			106

ZUCROE	SCO	LS	7-460K			106
FADVUC	SCO	HS	75-300K (from figure axis)			107
GAKKOS03	SCO	HS	1.8-375K			108
GAKKOS05	SCO	LS	1.8-375K			108
GAKKOS04	HS	HS	1.8-375K			108
PASGOF	SCO	HS	100-300K			109
PASGOF01	SCO	LS	100-300K			109
BAYZEH01	SCO	HS	5-300K (from figure axis)			110
BAYZEH	SCO	LS	5-300K (from figure axis)			110
POGMED	HS	HS	4-300K			111
FIJTIC	HS	HS	2-300K			112
QOMDAY	SCO	HS	3-300K			113
QOMDAY01	SCO	MIX.	3-300K			113
QOMDAY02	SCO	LS	3-300K			113
QUNYAA	SCO	LS	2-400K			114
QUNYEE01	SCO	HS	2-400K			114
QUNYEE	SCO	LS	2-400K			114
VASYOF	HS	HS	5-350K			115
VASYUL01	SCO	HS	5-350K			115
VASYUL	SCO	LS	5-350K			115
VASZEW	SCO	HS	5-350K			115

VASZEW01	SCO	LS	5-350K			115
MIWJUZ	HS	HS	2-300K			116
KUHRUC	HS	HS	30-300K			117
DUWHIO03	SCO	HS	290-450K			118
DUWHIO	SCO	LS	290-450K			118
WIDGEW	SCO	HS	50-290K			119
WIDGIA	HS	HS	50-290K			119
WIDGOG	HS	HS	50-290K			119
ZUBNAO01	SCO	HS	10-395K			120
ZUBNAO	SCO	LS	10-395K			120
ZUBNES	SCO	HS	10-395K			120
ZUBNIW01	SCO	LS	10-395K			120
ZUBNOC	HS	HS	10-395K			120
ATAVAU	SCO	LS	10-400K			121
ATAVEY01	SCO	HS	10-400K			121
ATAVEY	SCO	LS	10-400K			121
DUKJAW01	SCO	HS	10-300K			122
DUKJAW	SCO	LS	10-300K			122
DUKJIE	HS	HS	10-300K			122
HAPDOU	HS	HS	2-300K			123
KULTAO04	SCO	HS	10-400K			124
KULTAO05	SCO	LS	10-400K			124

POWSID01	SCO	HS	5-300K<br>(from figure axis)			125
TISWIC	SCO	HS	1.8-298K			126
TISWIC01	SCO	LS	1.8-298K			126
BOCYOK02	SCO	HS	4-400K			127
BOCYOK	SCO	LS	4-400K			127
BOCZEB03	SCO	HS	4-400K			127
BOCZEB	SCO	LS	4-400K			127
UZAXUQ	SCO	HS	5-300K<br>(from figure axis)			128
ZOYKUV01	SCO	HS	4-300K			129
ZOYKUV	SCO	LS	4-300K			129
AJAXEP	SCO	HS	2-300K<br>(from figure axis)			130
FIWXOZ01	SCO	MIX.	4-350K			30
XEFDER02	SCO	HS	4-300K<br>(from figure axis)			131
XEFDER06	SCO	MIX.	4-300K<br>(from figure axis)			131
ZEQMEP02	SCO	HS	80-280K<br>(from figure axis)			132
JOJQEE	HS	HS	4-290K	80K		133
GOGDUB	LS	LS		78K,296K		134
EMIPIZ01	SCO	HS	5-300K<br>(from figure axis)			22,135

NEMSAZ	SCO	HS	99-303K	78K,298K		136
NEMSED	SCO	HS	99-303K	78K,298K		136
UJEDOC01	SCO	HS	99-298K	80K,298K		137
UJEDOC	SCO	LS	99-298K	80K,298K		137
ILACON	SCO	LS	2-300K			138
LAQYIM	SCO	MIX.	275-400K			139
HECCOJ01	SCO	HS	5-300K			140
HECCOJ	SCO	LS	5-300K			140
NERDIA01	SCO	HS	5-400K (from figure axis)			141
NERDIA	SCO	LS	5-400K (from figure axis)			141
SOJKUY	HS	HS	2-400K			142
VEVQOE	SCO	MIX.	5-300K (from figure axis)			143
NETBUJ	SCO	LS	4.2-420K			144
FEKYEA	SCO	LS	100-385K (from figure axis)			145
MEQXIS01	SCO	HS	5-300K (from figure axis)			146
MEQXIS	SCO	LS	5-300K (from figure axis)			146
UCIGUJ	HS	HS	1.7-300K			147
COLJAO	HS	HS	99-305K			148
RIZSOI	SCO	LS	94-383K			149

KOKKUR	SCO	HS	5-300K (from figure axis)			150
KOKKUR02	SCO	LS	5-300K (from figure axis)			150
PUBBOD	SCO	LS	94-383K			151
TETXAR	SCO	LS	89-343K	298K		152
TESMAF	SCO	MIX.	89-303K	78K,298K		153
TESLUY	LS	LS	89-303K	78K,298K		153
ZOKBOQ	SCO	HS	2-300K			154
QERDOH	SCO	LS	10-400K	80K,293K		155
EGIPOC	SCO	LS	100-400K			156
ABUFOV	LS	LS	1.9-300K (from figure axis)			157
ABUGAI	LS	LS	1.9-300K (from figure axis)			157
ILAWUP	HS	HS	5-200K (from figure axis)			158
ZIKKUC04	SCO	HS	100-400K (from figure axis)			159
ZIKKUC	SCO	MIX.	100-400K (from figure axis)			159
ZIKMAK09	SCO	MIX.	100-400K (from figure axis)			159
ZIKMAK05	SCO	LS	100-400K (from figure axis)			159

MEQVEM02	SCO	HS	5-300K (from figure axis)			146
MEQVEM	SCO	LS	5-300K (from figure axis)			146
OCEVEA01	SCO	HS	2-400K	8.6K,100K, 200K,293K, 330K,350K		160
OCEVEA	SCO	LS	2-400K	8.6K,100K, 200K,293K, 330K,350K		160
LARZAF	HS	HS	79K,89K, 293K,303K (4 points)			161
DOMQED	LS	LS	2-400K			162
DOMQIH	LS	LS	2-400K			162
DOMQON	HS	HS	2-400K			162
DOMQUT02	SCO	HS	2-400K			162
DOMQUT01	SCO	MIX.	2-400K			162
DOMQUT	SCO	LS	2-400K			162
JAQVIG	SCO	LS	94-303K			163
JAQVOM	SCO	LS	94-383K			163
JAQVUS	SCO	LS	94-303K			163
JAQWAZ	SCO	LS	94-303K			163
XADFEN	SCO	LS	94-383K			164
QERDIB	SCO	HS	10-400K	80K,165K, 293K		155

QERDIB01	SCO	LS	10-400K	80K,165K,<br>293K		155
QIDJET	SCO	HS	2-400K			165
QIDJET01	SCO	LS	2-400K			165
QIDJIX	SCO	LS	2-400K			165
QIDJOD	SCO	LS	2-400K			165
QIDJUJ	LS	LS	2-400K			165
QIDKAQ	SCO	LS	2-400K			165
QIDKEU	SCO	LS	2-400K			165
QIDKOE	SCO	LS	2-400K			165
QIDKUK	SCO	LS	2-400K			165
QIDLUL	SCO	HS	2-400K			165
QIDLUL01	SCO	MIX.	2-400K			165
DUSMAH	SCO	HS	10-300K	77K,298K		166
DUSMAH03	SCO	LS	10-300K	77K,298K		166
DUSMUB	HS	HS	10-300K	77K,298K		166
QOZNOL	HS	HS	20-300K<br>(from figure axis)			167
AWUKEK01	HS	HS	10-300K			168
FOSBOG	HS	HS	10-300K			169
FOSCAT	SCO	MIX.	10-300K			169
FOSCEX	SCO	HS	10-300K			169
FOSCEX01	SCO	MIX.	10-300K			169
FOSCOH	SCO	MIX.	10-300K			169

GIVZIV	HS	HS	5-400K (from figure axis)			170
GIVZAN	SCO	LS	5-400K (from figure axis)			170
GIVZER	SCO	MIX.	5-400K (from figure axis)			170
HECBUO	HS	HS	5-300K			140
HECCEZ	HS	HS	5-300K			140
HECCID	HS	HS	5-300K			140
HIXDOK01	SCO	HS	5-300K			171
HIXDOK	SCO	LS	5-300K			171
HOMXIT	SCO	HS	5-370K			172
HOMXIT01	SCO	MIX.	5-370K			172
HOMXIT02	SCO	LS	5-370K			172
ITINOP	SCO	HS	5-300K			173
ITINOP03	SCO	LS	5-300K			173
IZADEU	SCO	HS	2-300K			168
IZADEU01	SCO	MIX.	2-300K			168
IZADOE	HS	HS	10-300K			168
IZADUK01	SCO	HS	10-300K (from figure axis)			168
IZADUK	SCO	LS	10-300K (from figure axis)			168
IZAFEW03	SCO	HS	10-400K (from figure axis,			168

			up to 500 K depending on compound)			
IZAFEW	SCO	LS	10-400K (from figure axis, up to 500 K depending on compound)			168
IZAFIA	SCO	LS	10-400K (from figure axis, up to 500 K depending on compound)			168
IZAFOG01	SCO	HS	10-400K (from figure axis, up to 500 K depending on compound)			168
IZAFOG	SCO	LS	10-400K (from figure axis, up to 500 K depending on compound)			168
IZAGAT02	SCO	MIX.	10-400K (from figure axis, up to 500 K depending on compound)			168
IZAGAT	SCO	LS	10-400K (from figure axis, up to 500 K			168

			depending on compound)			
KALWUR	SCO	MIX.	2-350K			174
KALXAY01	SCO	HS	2-350K			174
KALXAY	SCO	LS	2-350K			174
MEGDEH	LS	LS	2-298K			175
NIXWEZ	SCO	MIX.	1.8-300K			176
NIXXAW	SCO	MIX.	1.8-300K			176
NIXXEA	HS	HS	1.8-300K			176
VILZAT	HS	HS	5-300K			177
VILZEX	HS	HS	5-300K			177
VILZOH	HS	HS	5-300K			177
VIMBAW	HS	HS	5-300K			177
VISCAD	SCO	HS	20-300K			178
XODCEB	LS	LS	5-300K			171
TULHEQ	SCO	LS	3-350K			179
TULHIU	HS	HS	3-350K			179
DULBUK02	SCO	HS	2-400K			180
DULBUK	SCO	LS	2-400K			180
DULCIZ	SCO	LS	2-400K			180
DULCOF	SCO	LS	2-400K			180
DULDAS	LS	LS	2-400K			180
DOQRAC	SCO	HS	2-400K			181

DOQRAC01	SCO	LS	2-400K			181
WEKJED	LS	LS		78K,RT		182
WEKJIH	LS	LS	5-300K			182
NEFSIA01	SCO	HS	2-300K<br>(from figure axis)			183
NEFSIA02	SCO	LS	2-300K<br>(from figure axis)			183
NUMBEC01	SCO	HS	4.5-295K			184
NUMBEC	HS	HS	4.5-295K			184
NUMBEC02	SCO	HS	4.5-295K			184
UZELAN	HS	HS	2-300K			185
TIHTAF01	SCO	HS	77-290K	80K,170K,<br>190K,210K,<br>225K,295K		186
TIHTAF	SCO	LS	77-290K	80K,170K,<br>190K,210K,<br>225K,295K		186
IQIJAT02	SCO	HS	150-200K<br>(from figure axis)			187
IQIJAT	SCO	LS	150-200K<br>(from figure axis)			187
BAXXAB01	SCO	HS	10-295K<br>(from figure axis)			188
YUYHOR01	SCO	HS	10-400K			189
YUYHOR	SCO	LS	10-400K			189

ABISOV	SCO	HS	2-300K (from figure axis)			190
ABISOV02	SCO	LS	2-300K (from figure axis)			190
ABITEM	SCO	HS	2-300K (from figure axis)			190
ABITEM01	SCO	LS	2-300K (from figure axis)			190
ABITUC01	SCO	HS	2-300K (from figure axis)			190
ABITUC	SCO	LS	2-300K (from figure axis)			190
ABIVAK	SCO	HS	2-300K (from figure axis)			190
ABIVAK01	SCO	LS	2-300K (from figure axis)			190
CECVUD	HS	HS	2-300K			191
EBOTOG	SCO	HS	2-300K (from figure axis)			190
EBOTOG01	SCO	LS	2-300K (from figure axis)			190
EBOVAU	HS	HS	2-300K (from figure axis)			190
LUWSUS01	SCO	HS	2-300K			192
LUWSUS	SCO	MIX.	2-300K			192
LUWSUS03	SCO	HS	5-300K (from figure axis)			193

LUWSUS02	SCO	MIX.	5-300K (from figure axis)			193
LUWSUS06	SCO	HS	5-400K (from figure axis)			193
LUWSUS05	SCO	MIX.	5-400K (from figure axis)			193
LUWSUS04	SCO	LS	5-400K (from figure axis)			193
LUWSUS09	SCO	HS	5-400K (from figure axis)			193
LUWSUS07	SCO	LS	5-400K (from figure axis)			193
LUWTED02	SCO	HS	2-360K			192
LUWTED01	SCO	MIX.	2-360K			192
LUWTED	SCO	LS	2-360K			192
WAHKEX01	SCO	HS	2-300K			194
WAHKEX	SCO	LS	2-300K			194
TEKHAU	HS	HS	5-300K	40K,298K		195
AFOYUS02	SCO	HS	2-395K			196
AFOYUS	SCO	LS	2-395K			196
AFUJOD01	SCO	HS	2-395K			196
AFOYOM	SCO	LS	2-395K			196
TEKHEY	HS	HS	5-300K	40K,298K		195
TEKHIC	HS	HS	5-300K	40K,298K		195
HOLSIN	HS	HS	5-300K			197

HOLSUZ	SCO	HS	5-300K			197
HOLTUA	HS	HS	5-300K			197
JAQQIB	LS	LS	4.2K-RT			198
LINRAC	HS	HS	1.8-300K			199
MIFRIF	HS	HS	2-300K	80K,300K		200
XUGGEO	SCO	HS	10-300K<br>(from figure axis)			201
XUGGEO02	SCO	LS	10-300K<br>(from figure axis)			201
XUGGEO01	SCO	HS	10-300K<br>(from figure axis)			201
XUGGEO03	SCO	LS	10-300K<br>(from figure axis)			201
XUGGIS01	SCO	HS	10-300K<br>(from figure axis)			201
XUGGIS	SCO	LS	10-300K<br>(from figure axis)			201
XUGHAL	SCO	HS	10-300K<br>(from figure axis)			201
XUGGIS02	SCO	LS	10-300K<br>(from figure axis)			201
XUGGUE	HS	HS	10-300K			201
HOLTAG	HS	HS	5-300K			197
HOLTOU	HS	HS	5-300K			197
MIFROL01	SCO	HS	2-300K	80K,300K		200

MIFROL	SCO	LS	2-300K	80K,300K		200
QUHWAT	SCO	HS	10-300K (from figure axis)			202
QUHWAT01	SCO	MIX.	10-300K (from figure axis)			202
QUHWAT02	HS	HS	10-300K (from figure axis)			202
HOLTEK	SCO	HS	5-300K			197
HOLSOT	SCO	LS	5-300K			197
MIFSEC	SCO	HS	2-300K	80K,300K		200
MIFSAY	SCO	LS	2-300K	80K,300K		200
BUNSIN	SCO	HS	5-300K (from figure axis)			203
BUNSIN01	SCO	LS	5-300K (from figure axis)			203
ETUHIM	SCO	HS	2-400K			204
ETUHIM02	SCO	LS	2-400K			204
ETUJEK	SCO	HS	2-400K			204
KIFLOD	SCO	LS	5-400K			205
KIFLUJ	SCO	MIX.	5-400K			205
KIFMAQ01	SCO	HS	5-400K			205
KIFMAQ	SCO	LS	5-400K			205
HAXHIY	LS	LS	4.2-400K			206
KEZPIO01	SCO	HS	5-350K (from figure axis)			207

KEZPIO	SCO	LS	5-350K (from figure axis)			207
KEZPEK	SCO	LS	5-350K (from figure axis)			207
YAMXAL	SCO	LS	5.01-320K			208
MELLOF02	SCO	HS	15-290K	5K,80K, 155K,200K, 293K		209
MELLOF01	SCO	MIX.	15-290K	5K,80K, 155K,200K, 293K		209
MELLOF	SCO	LS	15-290K	5K,80K, 155K,200K, 293K		209
NAVYEP01	SCO	LS	5-300K			47
NOZJAN	HS	HS	80K,300K (2 points)			210
PASDOD	SCO	HS		77K,295K		49
QIDLAR	LS	LS	2-400K			165
QIDLEV	LS	LS	2-400K			165
QIDLIZ	LS	LS	2-400K			165
QIDLOF	LS	LS	2-400K			165
DUCFOW	LS	LS	303-406K	100K,340K		211
NESWAK	SCO	LS	10-290K (from figure axis)			16
YUZTUJ	SCO	LS	5-480K			21

FENFOW	LS	LS	2-295K			212
TEKHOI	HS	HS	5-300K	40K,298K		195
TEKHUO	HS	HS	5-300K	40K,298K		195
QIDKIY	SCO	LS	2-400K			165
PIVFEG	SCO	HS	4.3-294K			213
VOJPAM	SCO	HS	5-250K (from figure axis)	80K,120K, 180K,293K		214
VIFNAC	SCO	HS	4.3-294K			215
VIFNAC01	SCO	MIX.	4.3-294K			215
NIGXUY01	SCO	HS	5-275K (from figure axis)			216
NIGXUY02	SCO	HS	5-275K (from figure axis)			216
OCOPAA	LS	LS	5-300K			217
RUCJEF	LS	LS	5-400K			218
YECMAW	SCO	LS	35-400K			219

Table S3. Excluded crystal structures during curation for FeN_6–SSD. The table lists the CSD refcodes of Fe(II) FeN_6 complexes that were retrieved from the CSD but excluded from the final curated dataset. Entries were excluded for several reasons, including solvent-related instability, where the spin state or spin-transition behavior changes upon solvent loss or desorption; unverified spin transition, where a spin transition was reported or suspected but the experimental evidence was ambiguous or inconclusive; ambiguous or nonstandard composition, where the chemical composition, coordination environment, or structural representation is nonstandard or inconsistent, making spin-state assignment or data preprocessing (e.g., descriptor generation) unreliable; insufficient or missing data, where key measurements required to evaluate spin state or transition (e.g., temperature-dependent data) were not available or not reported; structural disorder, where positional disorder or partial occupancy complicates structural interpretation or preprocessing; and photo-induced (non-thermal) transition, where the spin transition is triggered by light irradiation rather than temperature change.

CSD	Spin State	Spin state (CIF file)	Note	Ref.
ARIXEF	HS	HS	Solvent-related instability	220
FILCAF	HS	HS	Unverified spin transition	221
FILCAF01	HS	HS	Unverified spin transition	221
POLLAE	HS	HS	Ambiguous or nonstandard composition	222
POLLEI	HS	HS	Ambiguous or nonstandard composition	222
POLLIM	HS	HS	Ambiguous or nonstandard composition	222
DEDNUW	HS	HS	Unverified spin transition	12
BOZCAV	SCO	LS	Insufficient or missing data	223
GIZZUL	LS	LS	Unverified spin transition	224

XIWGIT	LS	LS	Insufficient or missing data	225
QAMDOY	LS	LS	Unverified spin transition	226
TUFXOI	LS	LS	Unverified spin transition	227
JAFCID	LS	LS	Unverified spin transition	22
JAFCOJ	LS	LS	Unverified spin transition	22
JETTIL	HS	HS	Unverified spin transition	228
HPZBFE05	SCO	MIX.	Insufficient or missing data	229
HPZBFE03	SCO	LS	Insufficient or missing data	229
ZAWHUA	LS	LS	Unverified spin transition	230
ZAWJAI	LS	LS	Unverified spin transition	230
WAZMIT	LS	LS	Insufficient or missing data	231
HOQFEZ	LS	LS	Unverified spin transition	32
WOJMAJ	SCO	HS	Structural disorder	232
TRPYFI03	LS	LS	Insufficient or missing data	233
IXUZOQ01	LS	LS	Unverified spin transition	234
ZEXCUA	LS	LS	Unverified spin transition	235
ENOZIR	LS	LS	Unverified spin transition	50
NIPPIM	LS	LS	Unverified spin transition	236
PYFEFE	HS	HS	Insufficient or missing data	237
MENTEF	HS	HS	Unverified spin transition	238
FEPICA	SCO	MIX.	Insufficient or missing data	239
QUQNAT01	SCO	HS	Insufficient or missing data	240
QUQNAT	SCO	LS	Insufficient or missing data	240

DEKHUW	LS	LS	Unverified spin transition	241
IFAFUR	LS	LS	Unverified spin transition	242
MEMSON	LS	LS	Unverified spin transition	243
NUZKOI	LS	LS	Unverified spin transition	244
SEGGOB	LS	LS	Unverified spin transition	245
WOBTIQ	LS	LS	Unverified spin transition	246
CEPZOM	SCO	HS	Unverified spin transition	247
RUSQUR	LS	LS	Unverified spin transition	248
NENTAB	LS	LS	Insufficient or missing data	249
KINQUT	LS	LS	Solvent-related instability	250
QAJKUH	LS	LS	Unverified spin transition	251
RUZNOP01	LS	LS	Unverified spin transition	252
ABIWEO	SCO	MIX.	Unverified spin transition	89
KINTOQ	SCO	HS	Structural disorder	250
QOSNIW01	HS	HS	Unverified spin transition	253
KAJXUQ	LS	LS	Insufficient or missing data	254
HIKGEQ	HS	HS	Unverified spin transition	255
QOSNOC	LS	LS	Ambiguous or nonstandard composition	253
UDUYOJ	SCO	HS	Insufficient or missing data	256
LAYQOS01	SCO	HS	Solvent-related instability	103
XOPJUH	SCO	HS	Unverified spin transition	257
QALYOU03	SCO	HS	Solvent-related instability	258

QALYOU	SCO	LS	Solvent-related instability	258
QAMDEQ01	LS	LS	Solvent-related instability	258
QAMDOA01	LS	LS	Solvent-related instability	258
QAMFAO	HS	HS	Unverified spin transition	258
HAYBEQ	LS	LS	Insufficient or missing data	259
FENGAJ	LS	LS	Insufficient or missing data	212
GASCAF	LS	LS	Unverified spin transition	260
YEBNUO	SCO	LS	Unverified spin transition	261
IBUXAE	LS	LS	Unverified spin transition	262
VEVYUQ	LS	LS	Unverified spin transition	263
WOMXAX	LS	LS	Unverified spin transition	264
XOWDES	LS	LS	Unverified spin transition	265
CAPRIV	LS	LS	Unverified spin transition	266
PARVEK	LS	LS	Unverified spin transition	267
QAMDUE	HS	HS	Unverified spin transition	226
QEGVON	HS	HS	Unverified spin transition	268
NOYJEQ	LS	LS	Unverified spin transition	269
CAZJET	LS	LS	Unverified spin transition	270
WEYVUR	SCO	LS	Unverified spin transition	271
WEYWAY	SCO	LS	Unverified spin transition	271
NUPNIV	SCO	LS	Insufficient or missing data	272
WUJCEK	HS	HS	Solvent-related instability	273
WUJCIO	HS	HS	Solvent-related instability	273

AXUJUA	LS	LS	Solvent-related instability	274
JOHKID	LS	LS	Solvent-related instability	275
CAZJIX	HS	HS	Unverified spin transition	270
WEYWEC	SCO	MIX.	Insufficient or missing data	276
PUBBUJ	LS	LS	Insufficient or missing data	151
FOZKAH	LS	LS	Unverified spin transition	277
ZOFJOT	LS	LS	Unverified spin transition	278
WAZVAU	HS	HS	Unverified spin transition	279
JALJAH	LS	LS	Unverified spin transition	280
LANTOJ	LS	LS	Unverified spin transition	281
NARWIM	HS	HS	Insufficient or missing data	282
DETTOL	LS	LS	Unverified spin transition	283
PAFZIF	LS	LS	Insufficient or missing data	284
ILAXAW	HS	HS	Unverified spin transition	158
RINQEN	SCO	LS	Unverified spin transition	285
LAQYIM01	HS	HS	Solvent-related instability	139
RIZQUO	HS	HS	Solvent-related instability	286
SAVYUM01	SCO	HS	Insufficient or missing data	287
SAVYUM	SCO	LS	Insufficient or missing data	287
SEDJIU	LS	LS	Unverified spin transition	288
SEDJOA	LS	LS	Unverified spin transition	288
SOJFUT	LS	LS	Unverified spin transition	289
TORCIO	LS	LS	Insufficient or missing data	290

VIFPOQ	LS	LS	Unverified spin transition	291
VILZIB	HS	HS	Unverified spin transition	177
VILZUN	HS	HS	Solvent-related instability	177
XEMGII	LS	LS	Unverified spin transition	292
NELGUG	LS	LS	Insufficient or missing data	293
EBORIW	LS	LS	Unverified spin transition	294
EBOROC	LS	LS	Unverified spin transition	294
LAMGEL	HS	HS	Unverified spin transition	295
RUFYUM	HS	HS	Unverified spin transition	296
BAXXAB	SCO	MIX.	Unverified spin transition	188
UHEFES	HS	HS	Unverified spin transition	297
RAXZEW	LS	LS	Unverified spin transition	298
RAKMOG	LS	LS	Unverified spin transition	299
RIYTIC	LS	LS	Unverified spin transition	300
RUFYOG01	LS	LS	Insufficient or missing data	293
DUBFOY01	SCO	HS	Photo-induced (non-thermal) transition	301
DUBFOY	SCO	LS	Photo-induced (non-thermal) transition	301
ZOYZUI01	LS	LS	Unverified spin transition	302
IROHUS	LS	LS	Unverified spin transition	303
IROHOM	LS	LS	Unverified spin transition	303
RIRHAB	LS	LS	Unverified spin transition	304
WUPKUN	LS	LS	Unverified spin transition	305

OFURIQ	LS	LS	Unverified spin transition	306
RIYTOI	LS	LS	Unverified spin transition	300
VASZIY	LS	LS	Unverified spin transition	307
VASZOE	LS	LS	Unverified spin transition	307
GUJJUQ	LS	LS	Unverified spin transition	308
TABNOA	LS	LS	Unverified spin transition	309
ECOHOT	LS	LS	Unverified spin transition	310
ECOHIN	LS	LS	Unverified spin transition	310
YATSOC	LS	LS	Unverified spin transition	311
RUKLOY	HS	HS	Unverified spin transition	312
RUKLUE	HS	HS	Unverified spin transition	312
FIWGIB	HS	HS	Unverified spin transition	313
WOJLUC	HS	HS	Unverified spin transition	232
FEJCAZ	HS	HS	Unverified spin transition	314
SACTOG01	SCO	HS	Structural disorder	315
SACTOG	SCO	LS	Structural disorder	315
RIZQOI	LS	LS	Solvent-related instability	286
YUYJAF	LS	LS	Unverified spin transition	189
LAYQOS	SCO	HS	Solvent-related instability	103
VIFNEG	SCO	LS	Structural disorder	215
NIGXUY	SCO	MIX.	Unverified spin transition	216

Section S2. Crystal Packing and CIF-Based Descriptor Analysis

S2.1 Crystal Packing Descriptors Derived from CIF Structures

To evaluate whether crystal-packing information contributes to the prediction of SCO behavior, several packing-related descriptors were computed directly from the CIF structures using the CSD Python API. These descriptors characterize the overall packing density, intermolecular interactions, and void space within the crystal lattice. Hereafter, this set of descriptors is referred to as CSD-param.

The following descriptors were calculated for each crystal structure:

- Crystal density, obtained from the calculated density of the crystal structure.
- Packing coefficient, representing the fraction of the unit-cell volume occupied by molecules.
- Void fraction, calculated using the void-volume analysis implemented in the CSD API. The probe radius was set to 1.2 Å and the grid spacing to 0.2 Å.
- Intermolecular hydrogen-bond count, defined as the number of intermolecular hydrogen bonds identified by the CSD API.
- Total intermolecular contact count, representing the number of intermolecular contacts detected within the crystal structure.
- Minimum intermolecular contact distance, defined as the shortest distance among all intermolecular contacts.

In addition, the number of intermolecular contacts shorter than 3.6 Å was counted to characterize the frequency of close intermolecular interactions in the crystal packing environment.

These descriptors were used to construct additional machine-learning models to assess whether crystal-level packing information provides complementary predictive power compared with the descriptors derived from isolated coordination complexes.

S2.2 Periodic MBTR Representation of CIF Structures

Periodic MBTR representations were generated directly from the CIF structures to evaluate whether crystal-level structural information improves the prediction of SCO behavior. The MBTR formalism used in this analysis is identical to that described in the main text (Section 2.2.4). Hereafter, this representation is referred to as MBTR-CIF.

In contrast to the molecular MBTR used in the main analysis, this representation was constructed from the full crystallographic unit cell with periodic boundary conditions. All atoms present in the CIF structure were included in the representation.

The MBTR-CIF representations were calculated using the DScribe library with periodic boundary conditions enabled. The same interaction orders were considered: k1 (atomic number distribution), k2 (inverse interatomic distances), and k3 (angular distributions of atomic triplets), using parameter settings based on those adopted in a previous study[316].

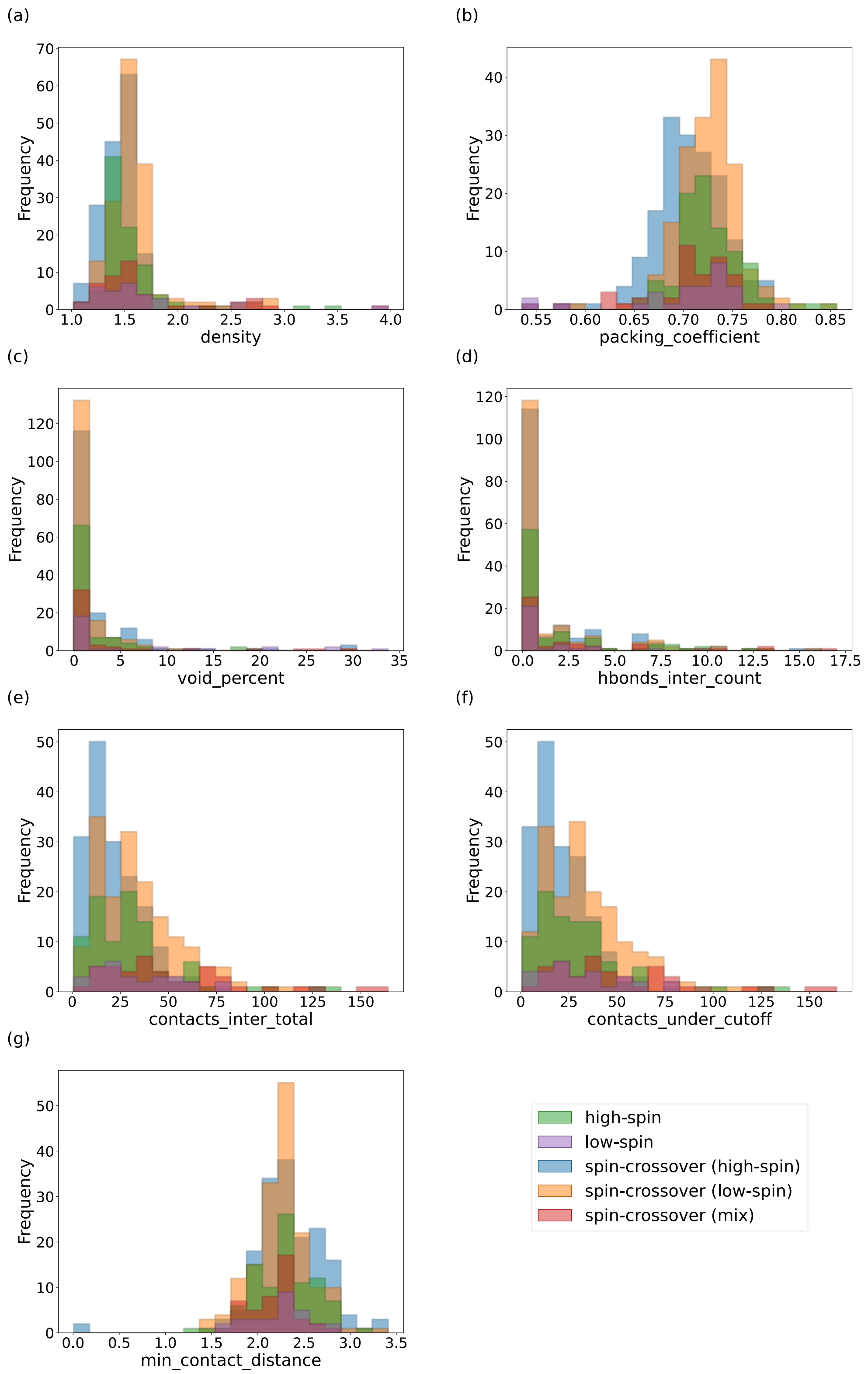

Figure S2. Distributions of crystal packing descriptors derived from CIF structures. Histograms of the crystal packing descriptors are shown, grouped by spin-state category. These descriptors are (a) density, (b) packing coefficient, (c) void

fraction, (d) intermolecular hydrogen-bond count, (e) total intermolecular contact count, (f) intermolecular contacts shorter than 3.6 Å, and (g) minimum intermolecular contact distance. The distributions largely overlap between SCO and non-SCO complexes within the same spin state, indicating that simple crystal packing descriptors alone do not clearly distinguish SCO-active and inactive complexes.

Table S4. Prediction accuracies for SCO classification using descriptors derived directly from CIF structures. The descriptor sets include crystal packing descriptors obtained from the CSD Python API (CSD-param) and periodic MBTR representations of the full crystal structures (MBTR-CIF). Prediction accuracies were evaluated using the Matthews correlation coefficient (MCC) and the F1 score for the classification of SCO-undergoing and non-SCO complexes in the high-spin (HS) and low-spin (LS) states. Three repetitions of five-fold cross-validation were conducted to derive the metric values. Mean values and standard deviations are reported. The evaluation protocol is identical to that used for Table 2 in the main text.

Descriptor	High spin and high-spin SCO		Low spin and low-spin SCO	
	MCC	F1	MCC	F1
MBTR-CIF	0.43 (0.11)	0.73 (0.043)	0.27 (0.21)	0.82 (0.038)
CSD-param	0.13 (0.11)	0.61 (0.050)	0.24 (0.20)	0.82 (0.037)

Table S5. Prediction accuracies for SCO classification obtained using group-based nested cross-validation. Prediction accuracies were evaluated using the Matthews correlation coefficient (MCC) and the F1 score. The task was predicting SCO complexes in the high-spin (low-spin) state. Three times 5-fold nested CV trials were conducted to derive the metric values. The mean and standard deviation values are reported. Suffix -Fe indicates a subset of a descriptor set containing Fe-related descriptors. Suffix + env. indicates concatenation of the descriptor set with the one-hot representations of solvent and counterion.

	Descriptor	High spin and high-spin SCO				Low spin and low-spin SCO			
		MCC		F1		MCC		F1	
		No-group	Group	No-group	Group	No-group	Group	No-group	Group
3D	MBTR	0.53 (0.10)	**0.52 (0.12)**	0.78 (0.044)	0.78 (0.059)	0.40 (0.22)	0.32 (0.32)	0.85 (0.048)	0.83 (0.087)
	MBTR + env.	0.53 (0.12)	0.49 (0.13)	0.78 (0.053)	0.77 (0.061)	0.37 (0.22)	0.26 (0.29)	0.85 (0.047)	0.83 (0.078)
	MBTR-Fe	**0.55 (0.068)**	0.48 (0.16)	0.79 (0.034)	0.76 (0.078)	0.28 (0.18)	0.23 (0.29)	0.83 (0.039)	0.82 (0.085)
	MBTR-Fe + env.	0.54 (0.085)	0.50 (0.13)	0.79 (0.038)	0.77 (0.067)	0.28 (0.21)	0.32 (0.22)	0.83 (0.044)	0.83 (0.070)
	OctaDist	0.50 (0.13)	0.47 (0.12)	0.77 (0.059)	0.76 (0.058)	0.037 (0.14)	0.025 (0.17)	0.78 (0.034)	0.78 (0.062)
	OctaDist + env.	0.47 (0.15)	0.47 (0.13)	0.76 (0.066)	0.76 (0.065)	0.12 (0.21)	-0.015 (0.12)	0.80 (0.039)	0.78 (0.068)
	MBTR-CIF	0.43 (0.11)	0.29 (0.17)	0.73 (0.044)	0.67 (0.10)	0.27 (0.22)	0.18 (0.29)	0.82 (0.039)	0.81 (0.078)
	CSD-param	0.13 (0.12)	0.074 (0.12)	0.61 (0.052)	0.58 (0.065)	0.24 (0.21)	0.20 (0.19)	0.82 (0.038)	0.82 (0.047)
2D	RAC	0.26 (0.090)	0.25 (0.18)	0.66 (0.040)	0.66 (0.089)	0.43 (0.21)	0.39 (0.27)	0.86 (0.049)	0.85 (0.068)
	RAC + env.	0.28 (0.11)	0.23 (0.14)	0.67 (0.049)	0.65 (0.075)	**0.45 (0.24)**	**0.41 (0.22)**	0.86 (0.055)	0.85 (0.063)
	RAC(no stereo)	0.24 (0.11)	0.25 (0.17)	0.66 (0.047)	0.66 (0.082)	0.37 (0.19)	0.32 (0.20)	0.85 (0.045)	0.83 (0.057)
	RAC(no stereo) + env.	0.24 (0.098)	0.24 (0.18)	0.66 (0.044)	0.65 (0.086)	0.36 (0.15)	0.28 (0.23)	0.84 (0.040)	0.83 (0.056)

	ECFP4	0.24<br>(0.095)	0.22<br>(0.14)	0.66<br>(0.043)	0.65<br>(0.070)	0.38<br>(0.24)	0.32<br>(0.26)	0.85<br>(0.052)	0.83<br>(0.075)
	ECFP4 + env.	0.25<br>(0.13)	0.19<br>(0.14)	0.66<br>(0.058)	0.64<br>(0.063)	0.33<br>(0.27)	0.27<br>(0.29)	0.84<br>(0.062)	0.81<br>(0.085)
	ECFP4-Fe	0.23<br>(0.12)	0.15<br>(0.13)	0.65<br>(0.050)	0.61<br>(0.071)	0.26<br>(0.21)	0.23<br>(0.26)	0.82<br>(0.051)	0.81<br>(0.078)
	ECFP4-Fe + env.	0.23<br>(0.16)	0.17<br>(0.14)	0.65<br>(0.069)	0.62<br>(0.082)	0.31<br>(0.20)	0.18<br>(0.28)	0.83<br>(0.046)	0.81<br>(0.068)

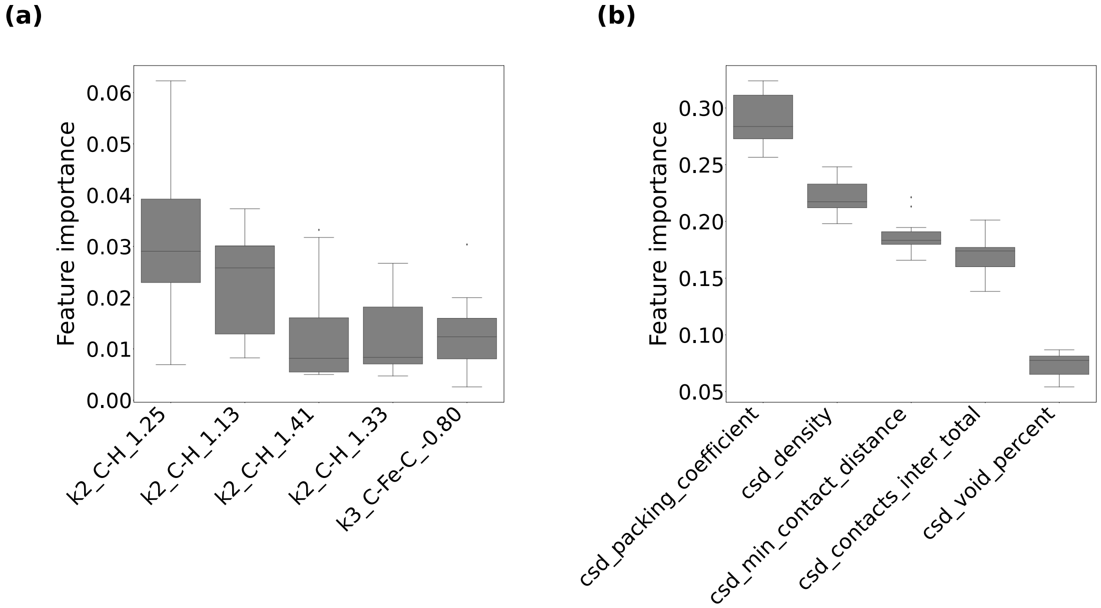

Figure S3. The top five contributed descriptors for the SCO classification models in the high-spin (HS) state using descriptors derived directly from CIF structures: (a) MBTR-CIF and (b) CSD-param. Each boxplot summarizes the 15 feature importance values obtained from the 15 random forest models (three repetitions of five-fold cross-validation), where the importance score is defined as the mean impurity decrease within decision trees. In MBTR-CIF, the descriptor notation follows the same convention as that used for MBTR in the main text. Here, k denotes the interaction order (i.e., the number of atoms involved in the geometric feature), followed by the atom tuple representing the chemical elements involved, and a real value representing the geometric feature (such as inverse distance or angular information). The MBTR-CIF representation was constructed from the full crystallographic unit cell with periodic boundary conditions. CSD-param corresponds to crystal packing descriptors derived directly from CIF structures using the CSD Python API. These descriptors include packing coefficient, crystal density, void fraction, intermolecular contact counts, and minimum intermolecular contact distance, which collectively characterize the packing density and intermolecular interactions within the crystal lattice.

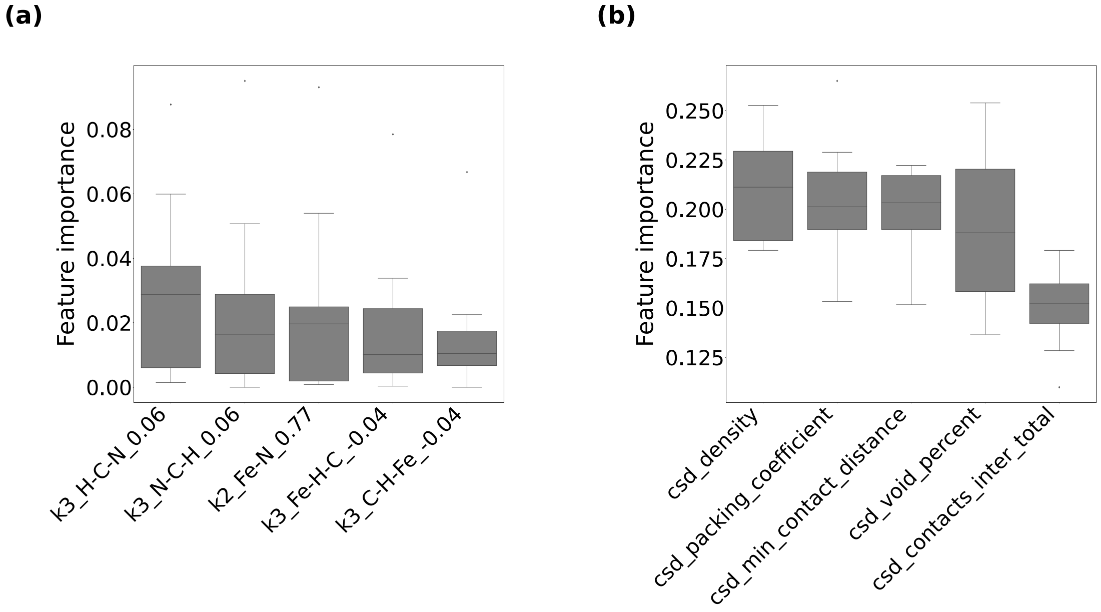

Figure S4. The top five contributed descriptors for the SCO classification models in the low-spin (LS) state using descriptors derived directly from CIF structures: (a) MBTR-CIF and (b) CSD-param. Each boxplot summarizes the 15 feature importance values obtained from the 15 random forest models (three repetitions of five-fold cross-validation), where the importance score is defined as the mean impurity decrease within decision trees. The descriptor definitions are identical to those described for Figure S3. MBTR-CIF represents periodic many-body tensor representations constructed from the full CIF structures, while CSD-param denotes crystal packing descriptors calculated using the CSD Python API.

Table S6. MCC values of Top-5 feature models for CIF-derived descriptors (MBTR-CIF and CSD-param). For SCO classification in the HS and LS states, two models were constructed using only the five most important features: the target task itself (Self) and the counterpart task (Cross). The evaluation procedure and notation for MCC and F1 are identical to those used in Table 2.

	Descriptor	High spin and high-spin SCO		Low spin and low-spin SCO	
		Self	Cross	Self	Cross
3D	MBTR	0.32 (0.11)	0.20 (0.16)	0.44 (0.15)	0.16 (0.20)
	CSD-param	0.12 (0.13)	0.16 (0.11)	0.30 (0.24)	0.32 (0.25)

Section S3. Feature Importance Analysis

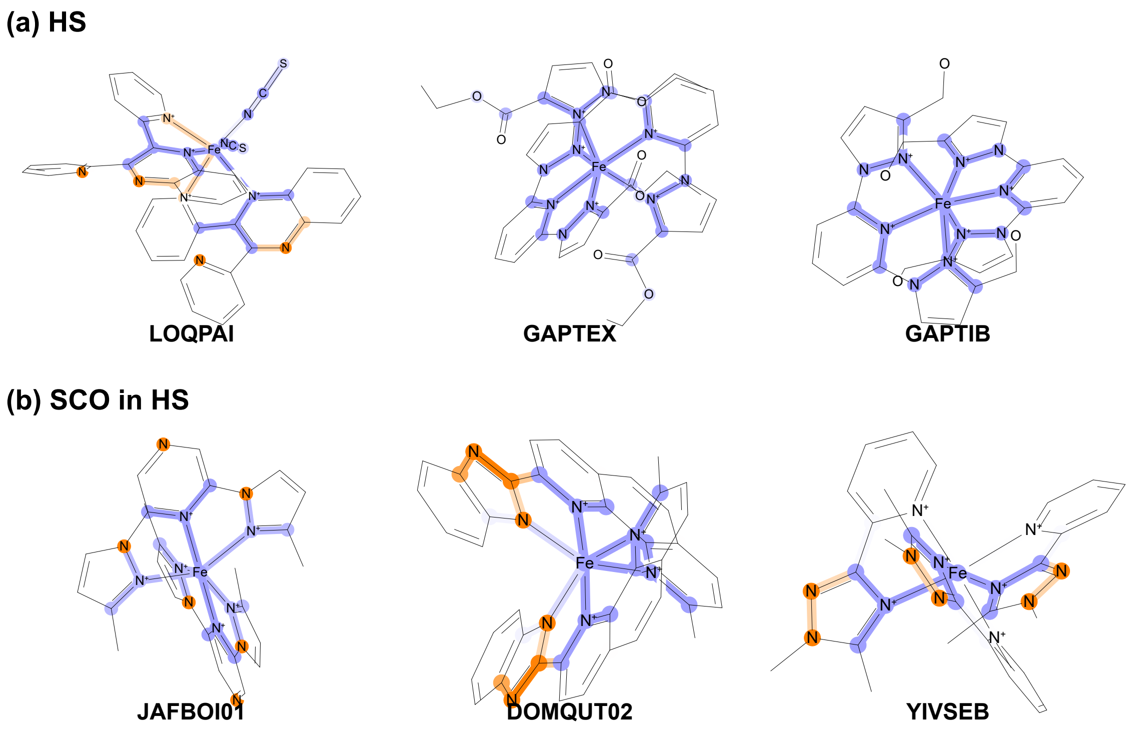

Figure S5. Visualization of ECFP4 contributions based on SHAP analysis for the HS state classification models. Each panel presents representative molecular structures with strong positive (orange) and negative (blue) SHAP contributions to the classification models. Substructures with strong SHAP values are highlighted to indicate both their magnitude and sign visually.

Section S4. Robustness Analyses of Model Evaluation

Table S7. Prediction accuracies obtained using global filtering and outer-fold filtering protocols. To assess the potential influence of descriptor filtering outside the outer cross-validation loop, prediction accuracies obtained using global filtering and outer-fold filtering protocols are compared.

	Descriptor	High spin and high-spin SCO				Low spin and low-spin SCO			
		MCC		F1		MCC		F1	
		Global filter	Outer-fold filter	Global filter	Outer-fold filter	Global filter	Outer-fold filter	Global filter	Outer-fold filter
3D	MBTR	0.56 (0.11)	0.53 (0.10)	0.79 (0.048)	0.78 (0.044)	0.40 (0.24)	0.40 (0.22)	0.85 (0.056)	0.85 (0.048)
	MBTR + env.	0.53 (0.11)	0.53 (0.12)	0.78 (0.049)	0.78 (0.053)	0.38 (0.23)	0.37 (0.22)	0.85 (0.051)	0.85 (0.047)
	MBTR-Fe	0.54 (0.055)	**0.55 (0.068)**	0.79 (0.026)	0.79 (0.034)	0.29 (0.19)	0.28 (0.18)	0.83 (0.043)	0.83 (0.039)
	MBTR-Fe + env.	**0.55 (0.079)**	0.54 (0.085)	0.79 (0.037)	0.79 (0.038)	0.29 (0.22)	0.28 (0.21)	0.83 (0.047)	0.83 (0.044)
	OctaDist	0.51 (0.13)	0.50 (0.13)	0.78 (0.060)	0.77 (0.059)	0.037 (0.14)	0.037 (0.14)	0.78 (0.034)	0.78 (0.034)
	OctaDist + env.	0.48 (0.13)	0.47 (0.15)	0.76 (0.058)	0.76 (0.066)	0.11 (0.18)	0.12 (0.21)	0.80 (0.034)	0.80 (0.039)
	MBTR-CIF	0.41 (0.12)	0.43 (0.11)	0.72 (0.049)	0.73 (0.044)	0.23 (0.20)	0.27 (0.22)	0.82 (0.035)	0.82 (0.039)
	CSD-param	0.13 (0.12)	0.13 (0.12)	0.61 (0.052)	0.61 (0.052)	0.20 (0.21)	0.24 (0.21)	0.82 (0.038)	0.82 (0.038)
2D	RAC	0.26 (0.097)	0.26 (0.090)	0.67 (0.043)	0.66 (0.040)	0.42 (0.21)	0.43 (0.21)	0.86 (0.049)	0.86 (0.049)
	RAC + env.	0.28 (0.12)	0.28 (0.11)	0.67 (0.053)	0.67 (0.049)	**0.45 (0.22)**	**0.45 (0.24)**	0.86 (0.053)	0.86 (0.055)
	RAC(no stereo)	0.23 (0.099)	0.24 (0.11)	0.65 (0.044)	0.66 (0.047)	0.39 (0.18)	0.37 (0.19)	0.85 (0.043)	0.85 (0.045)
	RAC(no stereo) + env.	0.26 (0.080)	0.24 (0.098)	0.67 (0.036)	0.66 (0.044)	0.37 (0.19)	0.36 (0.15)	0.84 (0.047)	0.84 (0.040)
	ECFP4	0.24	0.24	0.65	0.66	0.40	0.38	0.85	0.85

		(0.12)	(0.095)	(0.053)	(0.043)	(0.24)	(0.24)	(0.052)	(0.052)
	ECFP4 + env.	0.26<br>(0.12)	0.25<br>(0.13)	0.66<br>(0.053)	0.66<br>(0.058)	0.34<br>(0.26)	0.33<br>(0.27)	0.84<br>(0.060)	0.84<br>(0.062)
	ECFP4-Fe	0.25<br>(0.11)	0.23<br>(0.12)	0.66<br>(0.045)	0.65<br>(0.050)	0.27<br>(0.22)	0.26<br>(0.21)	0.82<br>(0.053)	0.82<br>(0.051)
	ECFP4-Fe + env.	0.26<br>(0.13)	0.23<br>(0.16)	0.67<br>(0.060)	0.65<br>(0.069)	0.33<br>(0.21)	0.31<br>(0.20)	0.84<br>(0.046)	0.83<br>(0.046)

Table S8. Prediction accuracies of Top-5 models obtained using a leakage-free outer-fold protocol. To assess the potential influence of information leakage during feature-importance estimation and Top-5 feature selection, prediction accuracies were obtained using a leakage-free outer-fold protocol in which these procedures were performed independently within each outer-fold training set. For comparison, results obtained using the original protocol are also shown.

	Descriptor	High spin and high-spin SCO				Low spin and low-spin SCO			
		MCC		F1		MCC		F1	
		leak	No-leak	leak	No-leak	leak	No-leak	leak	No-leak
3D	MBTR	**0.59 (0.12)**	0.58 (0.084)	0.81 (0.054)	0.81 (0.037)	0.51 (0.19)	0.34 (0.24)	0.88 (0.044)	0.84 (0.054)
	MBTR + env.	**0.59 (0.12)**	**0.59 (0.081)**	0.81 (0.054)	0.81 (0.035)	0.49 (0.23)	0.37 (0.17)	0.87 (0.054)	0.85 (0.041)
	MBTR-Fe	0.56 (0.086)	0.50 (0.12)	0.80 (0.041)	0.77 (0.052)	0.35 (0.28)	0.33 (0.24)	0.84 (0.062)	0.84 (0.056)
	MBTR-Fe + env.	0.56 (0.10)	0.45 (0.14)	0.80 (0.048)	0.75 (0.064)	0.29 (0.21)	0.32 (0.19)	0.83 (0.051)	0.83 (0.047)
	OctaDist	0.50 (0.12)	0.50 (0.12)	0.77 (0.053)	0.77 (0.052)	0.048 (0.14)	0.048 (0.14)	0.78 (0.033)	0.78 (0.033)
	OctaDist + env.	0.49 (0.12)	0.51 (0.14)	0.77 (0.055)	0.78 (0.065)	0.054 (0.15)	0.043 (0.16)	0.79 (0.029)	0.79 (0.034)
	MBTR-CIF	0.32 (0.11)	0.28 (0.13)	0.69 (0.049)	0.68 (0.057)	0.44 (0.15)	0.29 (0.20)	0.86 (0.036)	0.83 (0.041)
	CSD-param	0.12 (0.13)	0.11 (0.12)	0.60 (0.059)	0.60 (0.053)	0.30 (0.24)	0.26 (0.21)	0.83 (0.042)	0.82 (0.041)
2D	RAC	0.19 (0.12)	0.21 (0.11)	0.63 (0.057)	0.64 (0.053)	**0.52 (0.22)**	**0.47 (0.25)**	0.88 (0.053)	0.86 (0.065)
	RAC + env.	0.25 (0.13)	0.20 (0.089)	0.65 (0.063)	0.63 (0.041)	**0.52 (0.24)**	0.43 (0.28)	0.88 (0.055)	0.86 (0.073)
	RAC(no stereo)	0.22 (0.12)	0.21 (0.11)	0.65 (0.051)	0.64 (0.048)	0.30 (0.20)	0.33 (0.16)	0.82 (0.050)	0.83 (0.043)
	RAC(no stereo) + env.	0.21 (0.12)	0.20 (0.091)	0.64 (0.055)	0.64 (0.039)	0.30 (0.20)	0.29 (0.20)	0.82 (0.050)	0.82 (0.055)
	ECFP4	0.33 (0.11)	0.23 (0.14)	0.69 (0.052)	0.64 (0.064)	0.28 (0.12)	0.22 (0.10)	0.71 (0.097)	0.72 (0.087)

	ECFP4 + env.	0.36<br>(0.11)	0.22<br>(0.17)	0.70<br>(0.049)	0.64<br>(0.074)	0.23<br>(0.11)	0.16<br>(0.14)	0.76<br>(0.068)	0.73<br>(0.067)
	ECFP4-Fe	0.27<br>(0.13)	0.24<br>(0.13)	0.65<br>(0.058)	0.64<br>(0.060)	0.26<br>(0.23)	0.27<br>(0.19)	0.79<br>(0.11)	0.79<br>(0.096)
	ECFP4-Fe + env.	0.24<br>(0.089)	0.20<br>(0.089)	0.65<br>(0.043)	0.62<br>(0.039)	0.21<br>(0.13)	0.20<br>(0.22)	0.76<br>(0.058)	0.76<br>(0.084)

References

(1) Groom, C. R.; Bruno, I. J.; Lightfoot, M. P.; Ward, S. C. The Cambridge Structural Database. *Acta Crystallogr. Sect. B Struct. Sci. Cryst. Eng. Mater.* **2016**, *72* (2), 171–179. https://doi.org/10.1107/S2052520616003954.

(2) OpenEye, Cadence Molecular Sciences. OEChem TK 20230424, OEDepict TK 20230424; OpenEye, Cadence Molecular Sciences, Santa Fe, NM, 2023. http://www.eyesopen.com/.

(3) Molecular Operating Environment (MOE), 2022.02; Chemical Computing Group ULC, 910-1010 Sherbrooke St. W., Montreal, QC H3A 2R7, 2025. https://www.chemcomp.com/.

(4) Macrae, C. F.; Sovago, I.; Cottrell, S. J.; Galek, P. T. A.; McCabe, P.; Pidcock, E.; Platings, M.; Shields, G. P.; Stevens, J. S.; Towler, M.; Wood, P. A. Mercury 4.0: From Visualization to Analysis, Design and Prediction. *J. Appl. Crystallogr.* **2020**, *53* (1), 226–235. https://doi.org/10.1107/S1600576719014092.

(5) Halcrow, M. A. Structure:Function Relationships in Molecular Spin-Crossover Complexes. *Chem. Soc. Rev.* **2011**, *40* (7), 4119–4142. https://doi.org/10.1039/C1CS15046D.

(6) Halcrow, M. A. The Spin-States and Spin-Transitions of Mononuclear Iron(II) Complexes of Nitrogen-Donor Ligands. *Polyhedron* **2007**, *26* (14), 3523–3576. https://doi.org/10.1016/j.poly.2007.03.033.

(7) Elhaïk, J.; Evans, D. J.; Kilner, C. A.; Halcrow, M. A. A Structural, Magnetic and Mössbauer Spectroscopic Study of an Unusual Angular Jahn–Teller Distortion in a Series of High-Spin Iron(II) Complexes. *Dalton Trans* **2005**, No. 9, 1693–1700. https://doi.org/10.1039/B502175H.

(8) Holland, J. M.; McAllister, J. A.; Kilner, C. A.; Thornton-Pett, M.; Bridgeman, A. J.; Halcrow, M. A. Stereochemical Effects on the Spin-State Transition Shown by Salts of [FeL2]2+ [L = 2,6-Di(Pyrazol-1-Yl)Pyridine]. *J. Chem. Soc. Dalton Trans.* **2002**, No. 4, 548–554. https://doi.org/10.1039/b108468m.

(9) Halcrow, M. A. Iron(II) Complexes of 2,6-Di(Pyrazol-1-Yl)Pyridines—A Versatile System for Spin-Crossover Research. *Coord. Chem. Rev.* **2009**, *253* (21–22), 2493–2514. https://doi.org/10.1016/j.ccr.2009.07.009.

(10) Elhaïk, J.; Kilner, C. A.; Halcrow, M. A. Structural Diversity in Iron(II) Complexes of 2,6-Di(Pyrazol-1-Yl)Pyridine and 2,6-Di(3-Methylpyrazol-1-Yl)Pyridine. *Dalton Trans* **2006**, No. 6, 823–830.

https://doi.org/10.1039/B510370C.
(11) Haryono, M.; Heinemann, F. W.; Petukhov, K.; Gieb, K.; Müller, P.; Grohmann, A. Parallel Crystallization of a “Static” and a Spin-Crossover Polymorph of an Iron(II) Complex from the Same Solution. *Eur. J. Inorg. Chem.* **2009**, *2009* (14), 2136–2143. https://doi.org/10.1002/ejic.200900130.
(12) Kilner, C. A.; Halcrow, M. A. An Iron(II) Complex of 2,6-Di(Pyrazol-1-Yl)Pyrazine That Crystallises in Three Forms, Two of Which Exhibit an Unusual Angular Jahn–Teller Distortion. *Polyhedron* **2006**, *25* (2), 235–240. https://doi.org/10.1016/j.poly.2005.06.034.
(13) Carbonera, C.; Kilner, Colin. A.; Létard, J.-F.; Halcrow, M. A. Anion Doping as a Probe of Cooperativity in the Molecular Spin-Crossover Compound $[FeL_2][BF_4]_2$ (L = 2,6-Di{pyrazol-1-Yl}pyridine). *Dalton Trans* **2007**, No. 13, 1284–1292. https://doi.org/10.1039/B618480D.
(14) Nihei, M.; Tahira, H.; Takahashi, N.; Otake, Y.; Yamamura, Y.; Saito, K.; Oshio, H. Multiple Bistability and Tristability with Dual Spin-State Conversions in $[Fe(Dpp)_2][Ni(Mnt)_2]_2 \cdot MeNO_2$. *J. Am. Chem. Soc.* **2010**, *132* (10), 3553–3560. https://doi.org/10.1021/ja910122r.
(15) Money, V. A.; Carbonera, C.; Elhaïk, J.; Halcrow, M. A.; Howard, J. A. K.; Létard, J. Interplay Between Kinetically Slow Thermal Spin-Crossover and Metastable High-Spin State Relaxation in an Iron(II) Complex with Similar $T_{1/2}$ and *T* (LIESST). *Chem. – Eur. J.* **2007**, *13* (19), 5503–5514. https://doi.org/10.1002/chem.200601312.
(16) Carbonera, C.; Sánchez Costa, J.; Money, V. A.; Elhaïk, J.; Howard, J. A. K.; Halcrow, M. A.; Létard, J.-F. Photomagnetic Properties of Iron(II) Spin Crossover Complexes of 2,6-Dipyrazolylpyridine and 2,6-Dipyrazolylpyrazine Ligands. *Dalton Trans* **2006**, No. 25, 3058–3066. https://doi.org/10.1039/B601366J.
(17) Pritchard, R.; Kilner, C. A.; Halcrow, M. A. Iron(II) Complexes with a Terpyridine Embrace Packing Motif Show Remarkably Consistent Cooperative Spin-Transitions. *Chem Commun* **2007**, No. 6, 577–579. https://doi.org/10.1039/B613402E.
(18) Money, V. A.; Elhaïk, J.; Halcrow, M. A.; Howard, J. A. K. The Thermal and Light Induced Spin Transition in $[FeL_2](BF_4)_2$ (L = 2,6-Dipyrazol-1-Yl-4-Hydroxymethylpyridine). *Dalton Trans* **2004**, No. 10, 1516–1518. https://doi.org/10.1039/B401155D.
(19) Madhu, N. T.; Salitros, I.; Schramm, F.; Klyatskaya, S.; Fuhr, O.;

Ruben, M. Above Room Temperature Spin Transition in a Series of Iron(II) Bis(Pyrazolyl)Pyridine Compounds. *Comptes Rendus Chim.* **2008**, *11* (10), 1166–1174. https://doi.org/10.1016/j.crci.2008.06.015.

(20) Chandrasekar, R.; Schramm, F.; Fuhr, O.; Ruben, M. An Iron(II) Spin-Transition Compound with Thiol Anchoring Groups. *Eur. J. Inorg. Chem.* **2008**, *2008* (17), 2649–2653. https://doi.org/10.1002/ejic.200800212.

(21) Šalitroš, I.; Pavlik, J.; Boča, R.; Fuhr, O.; Rajadurai, C.; Ruben, M. Supramolecular Lattice-Solvent Control of Iron(Ii) Spin Transition Parameters. *CrystEngComm* **2010**, *12* (8), 2361. https://doi.org/10.1039/c002082f.

(22) Elhaïk, J.; Money, V. A.; Barrett, S. A.; Kilner, C. A.; Evans, I. R.; Halcrow, M. A. The Spin-States and Spin-Crossover Behaviour of Iron(II) Complexes of 2,6-Dipyrazol-1-Ylpyrazine Derivatives. *Dalton Trans* **2003**, No. 10, 2053–2060. https://doi.org/10.1039/B210368K.

(23) Money, V. A.; Elhaïk, J.; Radosavljevic Evans, I.; Halcrow, M. A.; Howard, J. A. K. A Study of the Thermal and Light Induced Spin Transition in $[FeL_2](BF_4)_2$ and $[FeL_2](ClO_4)_2$ L = 2,6-Di(3-Methylpyrazol-1-Yl)Pyrazine. *Dalton Trans* **2004**, No. 1, 65–69. https://doi.org/10.1039/B311262B.

(24) Pritchard, R.; Lazar, H.; Barrett, S. A.; Kilner, C. A.; Asthana, S.; Carbonera, C.; Létard, J.-F.; Halcrow, M. A. Thermal and Light-Induced Spin-Transitions in Iron(Ii) Complexes of 2,6-Bis(4-Halopyrazolyl)Pyridines: The Influence of Polymorphism on a Spin-Crossover Compound. *Dalton Trans.* **2009**, No. 33, 6656. https://doi.org/10.1039/b907094j.

(25) Reger, D. L.; Gardinier, J. R.; Elgin, J. D.; Smith, M. D.; Hautot, D.; Long, G. J.; Grandjean, F. Structure−Function Correlations in Iron(II) Tris(Pyrazolyl)Borate Spin-State Crossover Complexes. *Inorg. Chem.* **2006**, *45* (22), 8862–8875. https://doi.org/10.1021/ic0607437.

(26) Cecchi, P.; Berrettoni, M.; Giorgetti, M.; Gioia Lobbia, G.; Calogero, S.; Stievano, L. The Effect of the 3-Trifluoromethyl Substituent in Polypyrazolylborato Complexes on the Iron(II) Spin State; X-Ray Diffraction and Absorption and Mössbauer Studies. *Inorganica Chim. Acta* **2001**, *318* (1–2), 67–76. https://doi.org/10.1016/S0020-1693(01)00399-1.

(27) Reger, D. L.; Elgin, J. D.; Smith, M. D.; Grandjean, F.; Rebbouh, L.; Long, G. J. Structural Identification of the Factors That Prevent an Electronic Spin-State Crossover in Fe[(C6H5)B(3-Mepz)3]2 (Pz=pyrazolyl Ring). *Polyhedron* **2006**, *25* (13), 2616–2622. https://doi.org/10.1016/j.poly.2006.03.014.

(28) Reger, D. L.; Gardinier, J. R.; Smith, M. D.; Shahin, A. M.; Long, G.

J.; Rebbouh, L.; Grandjean, F. Polymorphism in Fe[(*p* -IC_6 H_4)B(3-Mepz)$_3$]$_2$ (Pz = Pyrazolyl): Impact of Supramolecular Structure on an Iron(II) Electronic Spin-State Crossover. *Inorg. Chem.* **2005**, *44* (6), 1852–1866. https://doi.org/10.1021/ic048406q.

(29) Iasco, O.; Boillot, M.-L.; Bellec, A.; Guillot, R.; Rivière, E.; Mazerat, S.; Nowak, S.; Morineau, D.; Brosseau, A.; Miserque, F.; Repain, V.; Mallah, T. The Disentangling of Hysteretic Spin Transition, Polymorphism and Metastability in Bistable Thin Films Formed by Sublimation of Bis(Scorpionate) Fe(II) Molecules. *J. Mater. Chem. C* **2017**, *5* (42), 11067–11075. https://doi.org/10.1039/C7TC03276E.

(30) Reger, D. L.; Gardinier, J. R.; Gemmill, W. R.; Smith, M. D.; Shahin, A. M.; Long, G. J.; Rebbouh, L.; Grandjean, F. Formation of Third Generation Poly(Pyrazolyl)Borate Ligands from Alkyne Coupling Reactions of Fe[(*p* -IC_6 H_4)B(3-Rpz)$_3$]$_2$ (R = H, Me; Pz = Pyrazolyl): Pathways toward Controlling an Iron(II) Electronic Spin-State Crossover. *J. Am. Chem. Soc.* **2005**, *127* (7), 2303–2316. https://doi.org/10.1021/ja044900h.

(31) Oliver, J. D.; Mullica, D. F.; Hutchinson, B. B.; Milligan, W. O. Iron-Nitrogen Bond Lengths in Low-Spin and High-Spin Iron(II) Complexes with Poly(Pyrazolyl)Borate Ligands. *Inorg. Chem.* **1980**, *19* (1), 165–169. https://doi.org/10.1021/ic50203a034.

(32) Hamon, P.; Thépot, J.; Le Floch, M.; Boulon, M.; Cador, O.; Golhen, S.; Ouahab, L.; Fadel, L.; Saillard, J.; Hamon, J. Dramatic Remote Substitutent Effects on the Electronic Spin State of Bis(Scorpionate) Iron(II) Complexes. *Angew. Chem. Int. Ed.* **2008**, *47* (45), 8687–8691. https://doi.org/10.1002/anie.200802567.

(33) Reger, D. L.; Little, C. A.; Smith, M. D.; Rheingold, A. L.; Lam, K.-C.; Concolino, T. L.; Long, G. J.; Hermann, R. P.; Grandjean, F. Synthetic, Structural, Magnetic, and Mössbauer Spectral Study of Fe[HC(3,5-Me2pz)3]2I2 and Its Spin-State Crossover Behavior. *Eur. J. Inorg. Chem.* **2002**, *2002* (5), 1190–1197. https://doi.org/10.1002/1099-0682(200205)2002:5%253C1190::AID-EJIC1190%253E3.0.CO;2-U.

(34) Kuzu, I.; Krummenacher, I.; Hewitt, I. J.; Lan, Y.; Mereacre, V.; Powell, A. K.; Höfer, P.; Harmer, J.; Breher, F. Syntheses, Structures and Electronic Properties of Zwitterionic Iron(II) and Cobalt(II) Complexes Featuring Ambidentate Tris(Pyrazolyl)Methanide Ligands. *Chem. – Eur. J.* **2009**, *15* (17), 4350–4365. https://doi.org/10.1002/chem.200802317.

(35) Reger, D. L.; Elgin, J. D.; Foley, E. A.; Smith, M. D.; Grandjean, F.; Long, G. J. Structural, Magnetic, and Mössbauer Spectral Study of the Electronic Spin-State Transition in [Fe{HC(3-Mepz)$_2$ (5-Mepz)}$_2$](BF$_4$)$_2$. *Inorg. Chem.* **2009**, *48* (19), 9393–9401. https://doi.org/10.1021/ic901259e.

(36) Moubaraki, B.; Leita, B. A.; Halder, G. J.; Batten, S. R.; Jensen, P.; Smith, J. P.; Cashion, J. D.; Kepert, C. J.; Létard, J.-F.; Murray, K. S. Structure, Magnetism and Photomagnetism of Mixed-Ligand Tris(Pyrazolyl)Methane Iron(Ii) Spin Crossover Compounds. *Dalton Trans.* **2007**, No. 39, 4413. https://doi.org/10.1039/b708773j.

(37) Anderson, P. A.; Astley, T.; Hitchman, M. A.; Keene, F. R.; Moubaraki, B.; Murray, K. S.; Skelton, B. W.; Tiekink, E. R. T.; Toftlund, H.; White, A. H. Structures and Spectra of Bis-Tripodal Iron(II) Chelates, [FeL2]2+, Where L = Tris(Pyrazol-1-Yl)Methane, Tris(Pyridin-2-Yl)Methane, Bis(Pyrazol-1-Yl)(Pyridin-2-Yl)Methane and Tris(Pyridin-2-Yl)Phosphine Oxide. Magnetism and Spin Crossover in the (Pz)3CH Case. *J. Chem. Soc. Dalton Trans.* **2000**, No. 20, 3505–3512. https://doi.org/10.1039/b003299i.

(38) Reger, D. L.; Little, C. A.; Rheingold, A. L.; Lam, M.; Liable-Sands, L. M.; Rhagitan, B.; Concolino, T.; Mohan, A.; Long, G. J.; Briois, V.; Grandjean, F. A Synthetic, Structural, Magnetic, and Spectral Study of Several {Fe[Tris(Pyrazolyl)Methane]$_2$ }(BF$_4$)$_2$ Complexes: Observation of an Unusual Spin-State Crossover. *Inorg. Chem.* **2001**, *40* (7), 1508–1520. https://doi.org/10.1021/ic001102t.

(39) Lazar, H. Z.; Forestier, T.; Barrett, S. A.; Kilner, C. A.; Létard, J.-F.; Halcrow, M. A. Thermal and Light-Induced Spin-Crossover in Salts of the Heptadentate Complex [Tris(4-{pyrazol-3-Yl}-3-Aza-3-Butenyl)Amine]Iron(Ii). *Dalton Trans.* **2007**, No. 38, 4276. https://doi.org/10.1039/b708971f.

(40) Hardie, M. J.; Kilner, C. A.; Halcrow, M. A. {Tris[4-(1 *H* -Pyrazol-3-Yl)-3-Azabut-3-Enyl]Amine}iron(II) Diperchlorate Monohydrate. *Acta Crystallogr. C* **2004**, *60* (4), m177–m179. https://doi.org/10.1107/S010827010400407X.

(41) Morgenstern-Badarau, I.; Lambert, F.; Deroche, A.; Cesario, M.; Guilhem, J.; Keita, B.; Nadjo, L. Sterically Hindered Iron(II) Complex of a New Tripodal Polyimidazole Ligand: Structure and Reactivity toward Superoxide. *Inorganica Chim. Acta* **1998**, *275–276*, 234–241. https://doi.org/10.1016/S0020-1693(97)05939-2.

(42) Nagasato, S.; Katsuki, I.; Motoda, Y.; Sunatsuki, Y.; Matsumoto, N.;

Kojima, M. Correlation among Crystal Shape, Absolute Configuration, and Circular Dichroism Spectrum of Enantiomorphs of Tris[2-(((2-Phenylimidazol-4-Yl)Methylidene)Amino)Ethyl]- Aminemetal(II) Nitrate−Methanol (1/1). *Inorg. Chem.* **2001**, *40* (11), 2534–2540. https://doi.org/10.1021/ic001254o.

(43) Hagiwara, H.; Matsumoto, N.; Iijima, S.; Kojima, M. Layered Iron(II) Spin Crossover Complex Constructed by NH···Br− Hydrogen Bonds with 2 K Wide Thermal Hysteresis, [FeIIH3LMe]Br·CF3SO3 (H3LMe= Tris[2-(((2-Methylimidazol-4-Yl)Methylidene)Amino)Ethyl]Amine). *Inorganica Chim. Acta* **2011**, *366* (1), 283–289. https://doi.org/10.1016/j.ica.2010.11.048.

(44) Seredyuk, M.; Gaspar, A. B.; Kusz, J.; Bednarek, G.; Gütlich, P. Variable-Temperature X-Ray Crystal Structure Determinations of {Fe[Tren(6-Mepy)$_3$]}(ClO$_4$)$_2$ and {Zn[Tren(6-Mepy)$_3$]}(ClO$_4$)$_2$ Compounds: Correlation of the Structural Data with Magnetic and Mössbauer Spectroscopy Data. *J. Appl. Crystallogr.* **2007**, *40* (6), 1135–1145. https://doi.org/10.1107/S0021889807048571.

(45) Seredyuk, M.; Gaspar, A. B.; Ksenofontov, V.; Galyametdinov, Y.; Kusz, J.; Gütlich, P. Does the Solid−Liquid Crystal Phase Transition Provoke the Spin-State Change in Spin-Crossover Metallomesogens? *J. Am. Chem. Soc.* **2008**, *130* (4), 1431–1439. https://doi.org/10.1021/ja077265z.

(46) Ohta, H.; Sunatsuki, Y.; Kojima, M.; Iijima, S.; Akashi, H.; Matsumoto, N. A Tripodal Ligand Containing Three Imidazole Groups Inducing Spin Crossover in Both Fe(II) and Fe(III) Complexes; Structures and Spin Crossover Behaviors of the Complexes. *Chem. Lett.* **2004**, *33* (3), 350–351. https://doi.org/10.1246/cl.2004.350.

(47) Yamada, M.; Fukumoto, E.; Ooidemizu, M.; Bréfuel, N.; Matsumoto, N.; Iijima, S.; Kojima, M.; Re, N.; Dahan, F.; Tuchagues, J.-P. A 2D Layered Spin Crossover Complex Constructed by NH···Cl$^-$ Hydrogen Bonds: [Fe^{II} H$_3$ L^{Me}]Cl·I$_3$ (H$_3$ L^{Me} = Tris[2-(((2-Methylimidazoyl-4-Yl)Methylidene)Amino)Ethyl]Amine. *Inorg. Chem.* **2005**, *44* (20), 6967–6974. https://doi.org/10.1021/ic050008f.

(48) Yamada, M.; Hagiwara, H.; Torigoe, H.; Matsumoto, N.; Kojima, M.; Dahan, F.; Tuchagues, J.; Re, N.; Iijima, S. A Variety of Spin-Crossover Behaviors Depending on the Counter Anion: Two-Dimensional Complexes Constructed by NH···Cl$^−$ Hydrogen Bonds, [Fe^{II} H$_3$ L^{Me}]Cl·X (X = PF$_6$$^−$, AsF$_6$$^−$, SbF$_6$$^−$, CF$_3$ SO$_3$$^−$; H$_3$ L^{Me} = Tris[2-{[(2-methylimidazol-4-yl)Methylidene]Amino}ethyl]Amine). *Chem. – Eur. J.* **2006**, *12* (17), 4536–4549. https://doi.org/10.1002/chem.200500972.

(49) Brewer, C.; Brewer, G.; Patil, G.; Sun, Y.; Viragh, C.; Butcher, R. J. Conformational Control of Spin State in Iron(II) Tripodal Imidazole Complexes. *Inorganica Chim. Acta* **2005**, *358* (12), 3441–3448. https://doi.org/10.1016/j.ica.2005.04.014.

(50) Alvarado, L.; Brewer, C.; Brewer, G.; Butcher, R. J.; Straka, A.; Viragh, C. Supramolecular Assemblies Prepared from an Iron(II) Tripodal Complex, Tetrafluoroborate, and Alkali Metal Cations. The Effect of Cation Size on Coordination Number, Anion Disorder and Hydrogen Bonding. *CrystEngComm* **2009**, *11* (11), 2297. https://doi.org/10.1039/b905061b.

(51) Brewer, C.; Brewer, G.; Luckett, C.; Marbury, G. S.; Viragh, C.; Beatty, A. M.; Scheidt, W. R. Proton Control of Oxidation and Spin State in a Series of Iron Tripodal Imidazole Complexes. *Inorg. Chem.* **2004**, *43* (7), 2402–2415. https://doi.org/10.1021/ic0351747.

(52) Søtofte, I.; Rasmussen, S. E.; Cyvin, S. J.; Brunvoll, J.; Hagen, G. Crystal Structure and Alleged Isomerism of Dithiocyanate-Tetrapyridine-Iron(II). *Acta Chem. Scand.* **1967**, *21*, 2028–2040. https://doi.org/10.3891/acta.chem.scand.21-2028.

(53) Roux, C.; Zarembowitch, J.; Gallois, B.; Granier, T.; Claude, R. Toward Ligand-Driven Light-Induced Spin Changing. Influence of the Configuration of 4 Styrylpyridine (Stpy) on the Magnetic Properties of FeII(Stpy)4(NCS)2 Complexes. Crystal Structures of the Spin-Crossover Species Fe(Trans-Stpy)4(NCS)2 and of the High-Spin Species Fe(Cis-Stpy)4(NCS)2. *Inorg. Chem.* **1994**, *33* (10), 2273–2279. https://doi.org/10.1021/ic00088a033.

(54) Carver, G.; Tregenna-Piggott, P. L. W.; Barra, A.-L.; Neels, A.; Stride, J. A. Spectroscopic and Structural Characterization of the $[Fe(Imidazole)_6]^{2+}$ Cation. *Inorg. Chem.* **2003**, *42* (18), 5771–5777. https://doi.org/10.1021/ic034110t.

(55) Hibbs, W.; Van Koningsbruggen, P. J.; Arif, A. M.; Shum, W. W.; Miller, J. S. One- and Two-Step Spin-Crossover Behavior of $[Fe^{II}(Isoxazole)_6]^{2+}$ and the Structure and Magnetic Properties of Triangular $[Fe^{III}_3O(OAc)_6(Isoxazole)_3][ClO_4]$. *Inorg. Chem.* **2003**, *42* (18), 5645–5653. https://doi.org/10.1021/ic034226p.

(56) Wiehl, L. Structures of Hexakis(1-Propyltetrazole)Iron(II) Bis(Tetrafluoroborate), $[Fe(CHN_4C_3H_7)_6](BF_4)_2$, Hexakis(1-Methyltetrazole)Iron(II) Bis(Tetrafluoroborate), $[Fe(CHN_4CH_3)_6](BF_4)_2$, and the Analogous Perchlorates. Their Relation to Spin Crossover Behaviour and Comparison of Debye–Waller Factors from Structure Determination and

Mössbauer Spectroscopy. *Acta Crystallogr. B* **1993**, *49* (2), 289–303. https://doi.org/10.1107/S0108768192009042.

(57) Kusz, J.; Spiering, H.; Gütlich, P. X-Ray Structure Study of the Light-Induced Metastable States of the Spin-Crossover Compound $[Fe(Mtz)_6](BF_4)_2$. *J. Appl. Crystallogr.* **2001**, *34* (3), 229–238. https://doi.org/10.1107/S0021889801000462.

(58) Urtizberea, A.; Roubeau, O. Switchable Slow Relaxation of Magnetization in the Native Low Temperature Phase of a Cooperative Spin-Crossover Compound. *Chem. Sci.* **2017**, *8* (3), 2290–2295. https://doi.org/10.1039/C6SC04737H.

(59) Hauser, A.; Gütlich, P.; Hinek, R.; Spiering, H.; Schollmeyer, D. The $[Fe(Etz)_6](BF_4)_2$ Spin-Crossover System—Part One: High-Spin ⇌ Low-Spin Transition in Two Lattice Sites. *Chem. – Eur. J.* **1996**, *2* (11), 1427–1434. https://doi.org/10.1002/chem.19960021115.

(60) Dova, E.; Stassen, A. F.; Driessen, R. A. J.; Sonneveld, E.; Goubitz, K.; Peschar, R.; Haasnoot, J. G.; Reedijk, J.; Schenk, H. Structure Determination of the $[Fe(Teec)_6](BF_4)_2$ Metal Complex from Laboratory and Synchrotron X-Ray Powder Diffraction Data with Grid-Search Techniques. *Acta Crystallogr. B* **2001**, *57* (4), 531–538. https://doi.org/10.1107/S010876810100828X.

(61) Dova, E.; Peschar, R.; Takata, M.; Nishibori, E.; Schenk, H.; Stassen, A. F.; Haasnoot, J. G. Low-Spin State Structure of $[Fe(Chloroethyltetrazole)_6](BF_4)_2$ Obtained from Synchrotron Powder Diffraction Data. *Chem. – Eur. J.* **2005**, *11* (20), 5855–5865. https://doi.org/10.1002/chem.200500036.

(62) Dova, E.; Peschar, R.; Sakata, M.; Kato, K.; Schenk, H. High-Spin- and Low-Spin-State Structures of $[Fe(Chloroethyltetrazole)_6](ClO_4)_2$ from Synchrotron Powder Diffraction Data. *Chem. – Eur. J.* **2006**, *12* (19), 5043–5052. https://doi.org/10.1002/chem.200501594.

(63) Stassen, A. F.; Dova, E.; Ensling, J.; Schenk, H.; Gütlich, P.; Haasnoot, J. G.; Reedijk, J. Spin Crossover in Hexakis(1-(2-Chloroethyl)-Tetrazole)Iron(II) Complexes; Synthesis and Magnetic Properties. *Inorganica Chim. Acta* **2002**, *335*, 61–68. https://doi.org/10.1016/S0020-1693(02)00759-4.

(64) Dova, E.; Peschar, R.; Sakata, M.; Kato, K.; Stassen, A. F.; Schenk, H.; Haasnoot, J. G. Structures of Fe^{II} Spin-Crossover Complexes from Synchrotron Powder-Diffraction Data. *Acta Crystallogr. B* **2004**, *60* (5), 528–538. https://doi.org/10.1107/S0108768104015356.

(65) Ozarowski, A.; McGarvey, B. R. EPR Study of Manganese(II) and Copper(II) in Single Crystals of the Spin-Crossover Complex Hexakis(1-Propyltetrazole)Iron(2+) Tetrafluoroborate(1-). *Inorg. Chem.* **1989**, *28* (12), 2262–2266. https://doi.org/10.1021/ic00311a005.

(66) Jeftić, J.; Hinek, R.; Capelli, S. C.; Hauser, A. Cooperativity in the Iron(II) Spin-Crossover Compound $[Fe(Ptz)_6](PF_6)_2$ under the Influence of External Pressure (Ptz = 1- *n* -Propyltetrazole). *Inorg. Chem.* **1997**, *36* (14), 3080–3087. https://doi.org/10.1021/ic961404o.

(67) Smeets, V.; Wolff, M.; Wolny, J. A.; Schünemann, V.; Dîrtu, M. M.; Ge, J.; Vanacken, J.; Moshchalkov, V.; Garcia, Y. Spin State Crossover, Vibrational, Computational, and Structural Studies of Fe^{II} 1-Isopropyl-1 *H* -tetrazole Derivatives. *Eur. J. Inorg. Chem.* **2018**, *2018* (3–4), 394–413. https://doi.org/10.1002/ejic.201700981.

(68) Greenaway, A. M.; Sinn, E. High-Spin and Low-Spin .Alpha.-Picolylamine Iron(II) Complexes. Effect of Ligand Reversal on Spin State. *J. Am. Chem. Soc.* **1978**, *100* (26), 8080–8084. https://doi.org/10.1021/ja00494a009.

(69) Katz, B. A.; Strouse, C. E. Molecular Transformations in the Solid State. Crystallographic Resolution of the Spin Isomers of Tris(2-Picolylamine)Iron(II) Dichloride and the Structural Relationship between the Methanol and Ethanol Solvates. *J. Am. Chem. Soc.* **1979**, *101* (21), 6214–6221. https://doi.org/10.1021/ja00515a010.

(70) Chernyshov, D.; Hostettler, M.; Törnroos, K. W.; Bürgi, H. Ordering Phenomena and Phase Transitions in a Spin-Crossover Compound—Uncovering the Nature of the Intermediate Phase of $[Fe(2\text{-pic})_3]Cl_2 \cdot EtOH$. *Angew. Chem. Int. Ed.* **2003**, *42* (32), 3825–3830. https://doi.org/10.1002/anie.200351834.

(71) Hostettler, M.; Törnroos, K. W.; Chernyshov, D.; Vangdal, B.; Bürgi, H. Challenges in Engineering Spin Crossover: Structures and Magnetic Properties of Six Alcohol Solvates of Iron(II) Tris(2-picolylamine) Dichloride. *Angew. Chem. Int. Ed.* **2004**, *43* (35), 4589–4594. https://doi.org/10.1002/anie.200460736.

(72) Törnroos, K. W.; Hostettler, M.; Chernyshov, D.; Vangdal, B.; Bürgi, H. Interplay of Spin Conversion and Structural Phase Transformations: Re-Entrant Phase Transitions in the 2-Propanol Solvate of Tris(2-picolylamine)Iron(II) Dichloride. *Chem. – Eur. J.* **2006**, *12* (24), 6207–6215. https://doi.org/10.1002/chem.200600547.

(73) Wiehl, L.; Kiel, G.; Koehler, C. P.; Spiering, H.; Guetlich, P. Structure Determination and Investigation of the High-Spin .Tautm. Low-Spin Transition of

Tris[2-(Aminomethyl)Pyridine]Iron(2+) Dibromide.Monoethanol. *Inorg. Chem.* **1986**, *25* (10), 1565–1571. https://doi.org/10.1021/ic00230a012.

(74) Zhao, X.-H.; Zhang, S.-L.; Shao, D.; Wang, X.-Y. Spin Crossover in $[Fe(2\text{-Picolylamine})_3]^{2+}$ Adjusted by Organosulfonate Anions. *Inorg. Chem.* **2015**, *54* (16), 7857–7867. https://doi.org/10.1021/acs.inorgchem.5b00870.

(75) Hayami, S.; Kawajiri, R.; Juhász, G.; Kawahara, T.; Hashiguchi, K.; Sato, O.; Inoue, K.; Maeda, Y. Study of Intermolecular Interaction for the Spin-Crossover Iron(II) Compounds. *Bull. Chem. Soc. Jpn.* **2003**, *76* (6), 1207–1213. https://doi.org/10.1246/bcsj.76.1207.

(76) Buron-Le Cointe, M.; Hébert, J.; Baldé, C.; Moisan, N.; Toupet, L.; Guionneau, P.; Létard, J. F.; Freysz, E.; Cailleau, H.; Collet, E. Intermolecular Control of Thermoswitching and Photoswitching Phenomena in Two Spin-Crossover Polymorphs. *Phys. Rev. B* **2012**, *85* (6), 064114. https://doi.org/10.1103/PhysRevB.85.064114.

(77) Létard, J.-F.; Guionneau, P.; Codjovi, E.; Lavastre, O.; Bravic, G.; Chasseau, D.; Kahn, O. Wide Thermal Hysteresis for the Mononuclear Spin-Crossover Compound *Cis* -Bis(Thiocyanato)Bis[*N* -(2‘-Pyridylmethylene)-4-(Phenylethynyl)Anilino]Iron(II). *J. Am. Chem. Soc.* **1997**, *119* (44), 10861–10862. https://doi.org/10.1021/ja972441x.

(78) Tailleur, E.; Marchivie, M.; Daro, N.; Chastanet, G.; Guionneau, P. Thermal Spin-Crossover with a Large Hysteresis Spanning Room Temperature in a Mononuclear Complex. *Chem. Commun.* **2017**, *53* (35), 4763–4766. https://doi.org/10.1039/C7CC01806A.

(79) Guionneau, P.; Létard, J.-F.; Yufit, D. S.; Chasseau, D.; Bravic, G.; Goeta, A. E.; Howard, J. A. K.; Kahn, O. Structural Approach of the Features of the Spin Crossover Transition in Iron (II) Compounds. *J. Mater. Chem.* **1999**, *9* (4), 985–994. https://doi.org/10.1039/a808075e.

(80) Létard, J.-F.; Kollmansberger, M.; Carbonera, C.; Marchivie, M.; Guionneau, P. Structural, Magnetic and Photomagnetic Study of the [Fe(PM–NEA)2(NCS)2] Spin Crossover Complex. *Comptes Rendus Chim.* **2008**, *11* (10), 1155–1165. https://doi.org/10.1016/j.crci.2008.05.009.

(81) Oso, Y.; Kanatsuki, D.; Saito, S.; Nogami, T.; Ishida, T. Spin-Crossover Transition Coupled with Another Solid–Solid Phase Transition for Iron(II) Thiocyanate Complexes Chelated with Alkylated *N* -(Di-2-Pyridylmethylene)Anilines. *Chem. Lett.* **2008**, *37* (7), 760–761. https://doi.org/10.1246/cl.2008.760.

(82) Onggo, D.; Hook, J. M.; Rae, A. D.; Goodwin, H. A. The Influence of Steric Effects in Substituted 2,2′-Bipyridine on the Spin State of Iron(II) in [FeN6]2+ Systems. *Inorganica Chim. Acta* **1990**, *173* (1), 19–30. https://doi.org/10.1016/S0020-1693(00)91050-8.

(83) Phan, H.; Hrudka, J. J.; Igimbayeva, D.; Lawson Daku, L. M.; Shatruk, M. A Simple Approach for Predicting the Spin State of Homoleptic Fe(II) Tris-Diimine Complexes. *J. Am. Chem. Soc.* **2017**, *139* (18), 6437–6447. https://doi.org/10.1021/jacs.7b02098.

(84) Craig, D.; Goodwin, H.; Onggo, D. Steric Influences on the Ground State of Iron(II) in the Tris(3,3’-Dimethyl-2,2’-Bipyridine)Iron(II) Ion. *Aust. J. Chem.* **1988**, *41* (8), 1157. https://doi.org/10.1071/CH9881157.

(85) Onggo, D.; Rae, A. D.; Goodwin, H. A. Coordination of the Strong Field Di-Imine 3,3′-Bipyridazine. Structural, Magnetic and Spectroscopic Properties of the Fe(II), Co(II) and Ni(II) Complexes. *Inorganica Chim. Acta* **1990**, *178* (2), 151–163. https://doi.org/10.1016/S0020-1693(00)86776-6.

(86) Stassen, A. F.; de Vos, M.; van Koningsbruggen, P. J.; Renz, F.; Ensling, J.; Kooijman, H.; Spek, A. L.; Haasnoot, J. G.; Gütlich, P.; Reedijk, J. Synthesis, Structure, and Magnetic Properties of a Tris[3-(2-pyridyl)-1,2,4-triazole]iron(II) Spin-Crossover Complex. *Eur. J. Inorg. Chem.* **2000**, *2000* (10), 2231–2237. https://doi.org/10.1002/1099-0682(200010)2000:10%253C2231::AID-EJIC2231%253E3.0.CO;2-B.

(87) Sugiyarto, K.; Craig, D.; Rae, A.; Goodwin, H. Structural and Electronic Properties of Iron(II) Complexes of 2-(1,2,4-Triazol-3-Yl)Pyridine and Substituted Derivatives. *Aust. J. Chem.* **1995**, *48* (1), 35. https://doi.org/10.1071/CH9950035.

(88) Niel, V.; Gaspar, A. B.; Muñoz, M. C.; Abarca, B.; Ballesteros, R.; Real, J. A. Spin Crossover Behavior in the Iron(II)−2-Pyridyl[1,2,3]Triazolo[1,5-*a*]Pyridine System: X-Ray Structure, Calorimetric, Magnetic, and Photomagnetic Studies. *Inorg. Chem.* **2003**, *42* (15), 4782–4788. https://doi.org/10.1021/ic034366z.

(89) Bradford, F. E.; Connor, L. P.; Kilner, C. A.; Halcrow, M. A. Iron Complexes of 3-(Pyrazinyl)-1,2,4-Triazole Ligands. *Polyhedron* **2004**, *23* (13), 2141–2151. https://doi.org/10.1016/j.poly.2004.06.018.

(90) Baker, A.; Goodwin, H. Iron(II) and Nickel(II) Complexes of 4,4′-Bithiazole: Spectral, Magnetic and Structural Studies. *Aust. J. Chem.* **1985**, *38* (6), 851. https://doi.org/10.1071/CH9850851.

(91) Martinez Lorente, M. A.; Dahan, F.; Petrouleas, V.; Bousseksou, A.; Tuchagues, J.-P. New Ferrous Complexes Based on the 2,2’-Biimidazole Ligand: Structural, Moessbauer, and Magnetic Properties of [FeII(bimH2)2(CH3OH)2](OAc)2, [FeII(bimH2)3]CO3, [FeII(bimH)2]n, and {FeII(Bim)}n. *Inorg. Chem.* **1995**, *34* (21), 5346–5357. https://doi.org/10.1021/ic00125a039.

(92) Boinnard, D.; Cassoux, P.; Petrouleas, V.; Savariault, J. M.; Tuchagues, J. P. Iron(II) Complexes of 2,2’-Biimidazole and 2,2’-Bibenzimidazole as Models of the Photosynthetic Mononuclear Non-Heme Ferrous Sites. Synthesis, Molecular and Crystal Structure, and Moessbauer and Magnetic Studies. *Inorg. Chem.* **1990**, *29* (20), 4114–4122. https://doi.org/10.1021/ic00345a041.

(93) García-López, V.; Palacios-Corella, M.; Clemente-León, M.; Coronado, E. Iron(II) Complex of 2-(1H-Pyrazol-1-Yl)Pyridine-4-Carboxylic Acid (ppCOOH) Suitable for Surface Deposition. *J. Coord. Chem.* **2018**, *71* (6), 763–775. https://doi.org/10.1080/00958972.2018.1430790.

(94) Huxel, T.; Leone, S.; Lan, Y.; Demeshko, S.; Klingele, J. 2-Amino-4-(2-pyridyl)Thiazole as Chelating Ligand: A Dinuclear Oxido-Bridged Ferric Complex and Mononuclear 3d Metal Complexes. *Eur. J. Inorg. Chem.* **2014**, *2014* (19), 3114–3124. https://doi.org/10.1002/ejic.201400041.

(95) Ni, Z.; Shores, M. P. Supramolecular Effects on Anion-Dependent Spin-State Switching Properties in Heteroleptic Iron(II) Complexes. *Inorg. Chem.* **2010**, *49* (22), 10727–10735. https://doi.org/10.1021/ic102004c.

(96) Ni, Z.; Fiedler, S. R.; Shores, M. P. Investigation of Anion-Dependence in the Spin-State Switching Properties of $[(H_2\ Bip)_2\ Fe(6\text{-}Mebpy)]X_2$. *Dalton Trans* **2011**, *40* (4), 944–950. https://doi.org/10.1039/C0DT01079K.

(97) Konno, M.; Mikami-Kido, M. Temperature- or Pressure-Induced Structure Changes of a Spin Crossover Fe(II) Complex; [Fe(Bpy)2(NCS)2]. *Bull. Chem. Soc. Jpn.* **1991**, *64* (2), 339–345. https://doi.org/10.1246/bcsj.64.339.

(98) Koenig, E.; Madeja, K.; Watson, K. J. Reversible Quintet-Singlet Transition in Dithiocyanato-Bis(2,2’-Dipyridyl)Iron(II). *J. Am. Chem. Soc.* **1968**, *90* (5), 1146–1153. https://doi.org/10.1021/ja01007a010.

(99) Gallois, B.; Real, J. A.; Hauw, C.; Zarembowitch, J. Structural Changes Associated with the Spin Transition in Bis(Isothiocyanato)Bis(1,10-Phenanthroline)Iron: A Single-Crystal x-Ray Investigation. *Inorg. Chem.* **1990**, *29* (6), 1152–1158. https://doi.org/10.1021/ic00331a009.

(100) Figg, D. C.; Herber, R. H.; Potenza, J. A. Thermally and Optically

Driven Spin-State Transitions in Bis(Isothiocyanato)Bis(5,6-Dimethylphenanthroline)Iron and Related Complexes and the Crystal Structure of Fe(2,9-Dmp)2 (NCS)2.Cntdot.1/4H2O. *Inorg. Chem.* **1992**, *31* (11), 2111–2117. https://doi.org/10.1021/ic00037a025.

(101) Real, J. A.; Munoz, M. C.; Andres, E.; Granier, T.; Gallois, B. Spin-Crossover Behavior in the Fe(Tap)2(NCS)2.Cntdot.nCH3CN System (Tap = 1,4,5,8-Tetraazaphenanthrene; n = 1, 1/2). Crystal Structures and Magnetic Properties of Both Solvates. *Inorg. Chem.* **1994**, *33* (16), 3587–3594. https://doi.org/10.1021/ic00094a023.

(102) Zhong, Z. J.; Tao, J.-Q.; Yu, Z.; Dun, C.-Y.; Liu, Y.-J.; You, X.-Z. A Stacking Spin-Crossover Iron(II) Compound with a Large Hysteresis †. *J. Chem. Soc. Dalton Trans.* **1998**, No. 3, 327–328. https://doi.org/10.1039/a706841g.

(103) Leita, B. A.; Moubaraki, B.; Murray, K. S.; Smith, J. P. Spin-Crossover in Dimeric Hydrogen-Bonded Iron(II) 2-(Pyrazolyl)-Pyridine and 2-(Imidazolyl)-Pyridine Complexes. *Polyhedron* **2005**, *24* (16–17), 2165–2172. https://doi.org/10.1016/j.poly.2005.03.033.

(104) Moliner, N.; Muñoz, M. C.; Van Koningsbruggen, P. J.; Real, J. Spin Crossover in Six-Coordinate [Fe(L)2(NCX)2] Compounds with L = DPQ = 2,3-Bis-(2′-Pyridyl)-Quinoxaline, ABPT = 4-Amino-3,5-Bis(Pyridin-2-Yl)-1,2,4-Triazole and X = S, Se: Synthesis, Magnetic Properties and Single Crystal Studies. *Inorganica Chim. Acta* **1998**, *274* (1), 1–6. https://doi.org/10.1016/S0020-1693(97)05954-9.

(105) Moliner, N.; Muñoz, M. C.; Létard, S.; Létard, J.-F.; Solans, X.; Burriel, R.; Castro, M.; Kahn, O.; Real, J. A. Spin-Crossover in the [Fe(Abpt)2(NCX)2] (X=S, Se) System: Structural, Magnetic, Calorimetric and Photomagnetic Studies. *Inorganica Chim. Acta* **1999**, *291* (1–2), 279–288. https://doi.org/10.1016/S0020-1693(99)00128-0.

(106) Kunkeler, P. J.; Van Koningsbruggen, P. J.; Cornelissen, J. P.; Van Der Horst, A. N.; Van Der Kraan, A. M.; Spek, A. L.; Haasnoot, J. G.; Reedijk, J. Novel Hybrid Spin Systems of 7,7‘,8,8‘-Tetracyanoquinodimethane (TCNQ) Radical Anions and 4-Amino-3,5-Bis(Pyridin-2-Yl)-1,2,4-Triazole (Abpt). Crystal Structure of [Fe(Abpt)$_2$ (TCNQ)$_2$] at 298 and 100 K, Mössbauer Spectroscopy, Magnetic Properties, and Infrared Spectroscopy of the Series [M^{II} (Abpt)$_2$ (TCNQ)$_2$] (M = Mn, Fe, Co, Ni, Cu, Zn). *J. Am. Chem. Soc.* **1996**, *118* (9), 2190–2197. https://doi.org/10.1021/ja943960s.

(107) Zhu, D.; Xu, Y.; Yu, Z.; Guo, Z.; Sang, H.; Liu, T.; You, X. A Novel

Bis(*Trans* -Thiocyanate)Iron(II) Spin-Transition Molecular Material with Bidentate Triaryltriazole Ligands and Its Bis(*Cis*- Thiocyanate)Iron(II) High-Spin Isomer. *Chem. Mater.* **2002**, *14* (2), 838–843. https://doi.org/10.1021/cm010688u.

(108) Galet, A.; Gaspar, A. B.; Muñoz, M. C.; Levchenko, G.; Real, J. A. Pressure Effect and Crystal Structure Reinvestigations on the Spin Crossover System: [Fe(Bt)$_2$ (NCS)$_2$] (Bt = 2,2'-Bithiazoline) Polymorphs **A** and **B**. *Inorg. Chem.* **2006**, *45* (24), 9670–9679. https://doi.org/10.1021/ic060729u.

(109) Real, J. A.; Gallois, B.; Granier, T.; Suez-Panama, F.; Zarembowitch, J. Comparative Investigation of the Spin-Crossover Compounds Fe(Btz)2(NCS)2 and Fe(Phen)2(NCS)2 (Where Btz = 2,2'-Bi-4,5-Dihydrothiazine and Phen = 1,10-Phenanthroline). Magnetic Properties and Thermal Dilatation Behavior and Crystal Structure of Fe(Btz)2(NCS)2 at 293 and 130 K. *Inorg. Chem.* **1992**, *31* (24), 4972–4979. https://doi.org/10.1021/ic00050a013.

(110) Takahashi, K.; Kawakami, T.; Gu, Z.; Einaga, Y.; Fujishima, A.; Sato, O. An Abrupt Spin Transition Based on Short S⋯S Contacts in a Novel Fe(II) Complex Whose Ligand Contains a 1,3-Dithiole Ring. *Chem Commun* **2003**, No. 18, 2374–2375. https://doi.org/10.1039/B308070F.

(111) Chue, C. F.; Lee, Z. C.; Wei, H. H.; Cheng, M. C.; Wang, Y. Mössbauer and Magnetic Studies of Copper(Ii)-Phthalocyanine Effect on the Spin States of Bis(n-o-Tolyl-2-Imidazolaldimine)Iron(Ii) Dithiocyanate. *Polyhedron* **1994**, *13* (15–16), 2259–2264. https://doi.org/10.1016/S0277-5387(00)88134-6.

(112) Ray, U.; Banerjee, D.; Liou, J.-C.; Lin, C.-N.; Lu, T.-H.; Sinha, C. Iron(II) and Nickel(II)-Thiocyanato Complexes of 1-Alkyl-2-(Arylazo)Imidazole: Single Crystal X-Ray Structure of [Fe(MeaaiEt)2(NCS)2] (MeaaiEt = 1-Ethyl-2(p-Tolylazo)Imidazole) and [Ni(MeaaiMe)(NCS)2(H2O)2] · 2DMF (MeaaiMe = 1-Methyl-2(p-Tolylazo)Imidazole). *Inorganica Chim. Acta* **2005**, *358* (4), 1019–1026. https://doi.org/10.1016/j.ica.2004.11.020.

(113) Bonnet, S.; Siegler, M. A.; Costa, J. S.; Molnár, G.; Bousseksou, A.; Spek, A. L.; Gamez, P.; Reedijk, J. A Two-Step Spin Crossover Mononuclear Iron(Ii) Complex with a [HS–LS–LS] Intermediate Phase. *Chem. Commun.* **2008**, No. 43, 5619. https://doi.org/10.1039/b811746b.

(114) Shuku, Y.; Suizu, R.; Awaga, K.; Sato, O. Fe(II) Spincrossover Complex of [1,2,5]Thiadiazolo[3,4-f][1,10]Phenanthroline. *CrystEngComm* **2009**, *11* (10), 2065. https://doi.org/10.1039/b906845g.

(115) Arcis-Castíllo, Z.; Zheng, S.; Siegler, M. A.; Roubeau, O.; Bedoui, S.; Bonnet, S. Tuning the Transition Temperature and Cooperativity of bapbpy-Based

Mononuclear Spin-Crossover Compounds: Interplay between Molecular and Crystal Engineering. *Chem. – Eur. J.* **2011**, *17* (52), 14826–14836. https://doi.org/10.1002/chem.201101301.

(116) Schmidt, S. O.; Kisslinger, S.; Würtele, C.; Bonnet, S.; Schindler, S.; Tuczek, F. Iron(II) Complexes Supported by a Tetradentate Ligand Providing a Strained Equatorial Coordination Environment: Geometric and Electronic-Structural Implications. *Z. Für Anorg. Allg. Chem.* **2013**, *639* (15), 2774–2778. https://doi.org/10.1002/zaac.201300425.

(117) Zheng, S.; Siegler, M. A.; Roubeau, O.; Bonnet, S. Influence of Selenocyanate Ligands on the Transition Temperature and Cooperativity of Bapbpy-Based Fe(II) Spin-Crossover Compounds. *Inorg. Chem.* **2014**, *53* (24), 13162–13173. https://doi.org/10.1021/ic502381m.

(118) Hernández, E. M.; Zheng, S.; Shepherd, H. J.; Yufit, D. S.; Ridier, K.; Bedoui, S.; Nicolazzi, W.; Velázquez, V.; Bonnet, S.; Molnár, G.; Bousseksou, A. Spatially Resolved Investigation and Control of the Bistability in Single Crystals of the [Fe(Bbpya) $(NCS)_2$] Spin Crossover Complex. *J. Phys. Chem. C* **2016**, *120* (48), 27608–27617. https://doi.org/10.1021/acs.jpcc.6b10258.

(119) Costa, J. S.; Lappalainen, K.; De Ruiter, G.; Quesada, M.; Tang, J.; Mutikainen, I.; Turpeinen, U.; Grunert, C. M.; Gütlich, P.; Lazar, H. Z.; Létard, J.-F.; Gamez, P.; Reedijk, J. Remarkable Steric Effects and Influence of Monodentate Axial Ligands L on the Spin-Crossover Properties of *Trans* -[Fe^{II} (N_4 Ligand)L] Complexes. *Inorg. Chem.* **2007**, *46* (10), 4079–4089. https://doi.org/10.1021/ic0624017.

(120) Li, N.; Xue, J.-P.; Liu, J.-L.; Wang, Y.-Y.; Yao, Z.-S.; Tao, J. Switchable on–off Spin-Crossover Properties of Iron(II) Compounds by Trimming Intermolecular Hydrogen Bonds. *Dalton Trans.* **2020**, *49* (4), 998–1001. https://doi.org/10.1039/C9DT04685B.

(121) Yang, Q.; Gao, C.; Wang, Y.-X.; Wang, B.-W.; Wang, Z.-M.; Gao, S. Two Magnetic Switching Complexes Based on the Fe^{II} Ion. *Inorg. Chem.* **2016**, *55* (16), 7805–7807. https://doi.org/10.1021/acs.inorgchem.6b00258.

(122) Yang, Q.; Cheng, X.; Gao, C.; Wang, B.; Wang, Z.; Gao, S. Structural Distortion Controlled Spin-Crossover Behavior. *Cryst. Growth Des.* **2015**, *15* (6), 2565–2567. https://doi.org/10.1021/acs.cgd.5b00175.

(123) Speed, S.; Pointillart, F.; Mulatier, J.; Guy, L.; Golhen, S.; Cador, O.; Le Guennic, B.; Riobé, F.; Maury, O.; Ouahab, L. Photophysical and Magnetic Properties in Complexes Containing 3d/4f Elements and Chiral Phenanthroline-

Based Helicate-Like Ligands. *Eur. J. Inorg. Chem.* **2017**, *2017* (14), 2100–2111. https://doi.org/10.1002/ejic.201601501.

(124) Yang, Q.; Cheng, X.; Wang, Y.; Wang, B.; Wang, Z.; Gao, S. Two-Step Magnetic Switching in a Mononuclear Iron(II) Complex around Room Temperature. *Dalton Trans.* **2015**, *44* (19), 8938–8941. https://doi.org/10.1039/C5DT00585J.

(125) Shepherd, H. J.; Palamarciuc, T.; Rosa, P.; Guionneau, P.; Molnár, G.; Létard, J.; Bousseksou, A. Antagonism between Extreme Negative Linear Compression and Spin Crossover in [Fe(Dpp)$_2$ (NCS)$_2$]·py. *Angew. Chem. Int. Ed.* **2012**, *51* (16), 3910–3914. https://doi.org/10.1002/anie.201108919.

(126) Tao, J.-Q.; Gu, Z.-G.; Wang, T.-W.; Yang, Q.-F.; Zuo, J.-L.; You, X.-Z. Spin-Crossover Iron(II) Complexes [Fe(Medpq)(Py)2(NCS)2] and [Fe(Medpq)(Py)2(NCSe)2]: Syntheses, Characterization and Magnetic Properties. *Inorganica Chim. Acta* **2007**, *360* (15), 4125–4132. https://doi.org/10.1016/j.ica.2007.06.006.

(127) De, S.; Chamoreau, L.-M.; El Said, H.; Li, Y.; Flambard, A.; Boillot, M.-L.; Tewary, S.; Rajaraman, G.; Lescouëzec, R. Thermally-Induced Spin Crossover and LIESST Effect in the Neutral [FeII(Mebik)2(NCX)2] Complexes: Variable-Temperature Structural, Magnetic, and Optical Studies (X = S, Se; Mebik = Bis(1-Methylimidazol-2-Yl)Ketone). *Front. Chem.* **2018**, *6*, 326. https://doi.org/10.3389/fchem.2018.00326.

(128) Wang, J.-L.; Liu, Q.; Lv, X.-J.; Wang, R.-L.; Duan, C.-Y.; Liu, T. Magnetic Fluorescent Bifunctional Spin-Crossover Complexes. *Dalton Trans.* **2016**, *45* (46), 18552–18558. https://doi.org/10.1039/C6DT03714C.

(129) Feltham, H. L. C.; Johnson, C.; Elliott, A. B. S.; Gordon, K. C.; Albrecht, M.; Brooker, S. "Tail" Tuning of Iron(II) Spin Crossover Temperature by 100 K. *Inorg. Chem.* **2015**, *54* (6), 2902–2909. https://doi.org/10.1021/ic503040f.

(130) Liu, W.; Gu, Z.-G.; Wang, R.; Zhou, X.-H.; Zuo, J.-L.; You, X.-Z. Transition Metal Complexes Based on Pyridine Ligand Containing Both Bis(2-Pyridyl) and 1,3-Dithiole-2-Ylidene Units: Syntheses, Structures, and Magnetic Studies. *Inorganica Chim. Acta* **2009**, *362* (8), 2556–2564. https://doi.org/10.1016/j.ica.2008.11.025.

(131) Reger, D. L.; Little, C. A.; Young, V. G.; Pink, M. Variable-Temperature X-Ray Structural Investigation of {Fe[HC(3,5-Me$_2$ Pz)$_3$]$_2$ }(BF$_4$)$_2$ (Pz = Pyrazolyl Ring): Observation of a Thermally Induced Spin State Change from All High Spin to an Equal High Spin-Low Spin Mixture, Concomitant with

the Onset of Nonmerohedral Twinning. *Inorg. Chem.* **2001**, *40* (12), 2870–2874. https://doi.org/10.1021/ic010056+.

(132) Lavrenova, L. G.; Strekalova, A. D.; Virovets, A. V.; Piryazev, D. A.; Daletskii, V. A.; Sheludyakova, L. A.; Mikhailovskaya, T. F.; Vasilevskii, S. F. Spin Crossover in the Coordination Compounds of Iron(II) with Tris(3,5-Dimethylpyrazol-1-Yl)Methane. *Russ. J. Coord. Chem.* **2012**, *38* (8), 507–514. https://doi.org/10.1134/S1070328412070081.

(133) Constable, E. C.; Baum, G.; Bill, E.; Dyson, R.; van Eldik, R.; Fenske, D.; Kaderli, S.; Morris, D.; Neubrand, A.; Neuburger, M.; Smith, D. R.; Wieghardt, K.; Zehnder, M.; Zuberbühler, A. D. Control of Iron(II) Spin States in 2,2′:6′,2″-Terpyridine Complexes through Ligand Substitution. *Chem. – Eur. J.* **1999**, *5* (2), 498–508. https://doi.org/10.1002/(SICI)1521-3765(19990201)5:2%253C498::AID-CHEM498%253E3.0.CO;2-V.

(134) Craig, D. C.; Scudder, M. L.; McHale, W.-A.; Goodwin, H. A. Structural Studies of Complexes of Tridentate Terimine Systems. Crystal Structure of Bis(2,2′:6′,2″-Terpyridine)Ruthenium(II) Perchlorate Hydrate, Bis(2,2′:6′,2″-Terpyridine)- Osmium(II) Perchlorate Hemihydrate and Bis((1,10-Phenanthrolin-2-Yl)(Pyridin-2-Yl)Amine)Iron(II) Tetrafluoroborate Dihydrate. *Aust. J. Chem.* **1998**, *51* (12), 1131. https://doi.org/10.1071/C98118.

(135) Money, V. A.; Radosavljevic Evans, I.; Elhaïk, J.; Halcrow, M. A.; Howard, J. A. K. An X-Ray Powder Diffraction Study of the Spin-Crossover Transition and Structure of Bis(2,6-Dipyrazol-1-Ylpyrazine)Iron(II) Perchlorate. *Acta Crystallogr. B* **2004**, *60* (1), 41–45. https://doi.org/10.1107/S0108768103027897.

(136) Sugiyarto, K. H.; Scudder, M. L.; Craig, D. C.; Goodwin, H. A. Electronic and Structural Properties of the Spin Crossover Systems Bis(2,6-Bis(Pyrazol-3-Yl)Pyridine)Iron(II) Thiocyanate and Selenocyanate. *Aust. J. Chem.* **2000**, *53* (9), 755. https://doi.org/10.1071/CH00087.

(137) Sugiyarto, K. H.; McHale, W.-A.; Craig, D. C.; Rae, A. D.; Scudder, M. L.; Goodwin, H. A. Spin Transition Centres Linked by the Nitroprusside Ion. The Cooperative Transition in Bis(2,6-Bis(Pyrazol-3-Yl)Pyridine)Iron(Ii) Nitroprusside. *Dalton Trans.* **2003**, No. 12, 2443. https://doi.org/10.1039/b301218b.

(138) Coronado, E.; Giménez-López, M. C.; Gimenez-Saiz, C.; Martínez-Agudo, J. M.; Romero, F. M. Synthesis, Structure and Magnetic Properties of Iron (II), Cobalt (II) and Nickel (II) Complexes of 2,6-Bis(Pyrazol-3-Yl)Pyridine and

Paramagnetic Counterions. *Polyhedron* **2003**, *22* (14–17), 2375–2380. https://doi.org/10.1016/S0277-5387(03)00242-0.

(139) Giménez-López, M. C.; Clemente-León, M.; Coronado, E.; Romero, F. M.; Shova, S.; Tuchagues, J. Structural Transformations and Magnetic Effects Induced by Solvent Exchange in the Spin Crossover Complex $[Fe(Bpp)_2][Cr(Bpy)(Ox)_2]_2$. *Eur. J. Inorg. Chem.* **2005**, *2005* (14), 2783–2787. https://doi.org/10.1002/ejic.200500233.

(140) Craig, G. A.; Costa, J. S.; Roubeau, O.; Teat, S. J.; Aromí, G. Local Coordination Geometry and Spin State in Novel Fe^{II} Complexes with 2,6-Bis(Pyrazol-3-yl)pyridine-Type Ligands as Controlled by Packing Forces: Structural Correlations. *Chem. – Eur. J.* **2012**, *18* (37), 11703–11715. https://doi.org/10.1002/chem.201200820.

(141) Jornet-Mollá, V.; Duan, Y.; Giménez-Saiz, C.; Tang, Y.; Li, P.; Romero, F. M.; Xiong, R. A Ferroelectric Iron(II) Spin Crossover Material. *Angew. Chem. Int. Ed.* **2017**, *56* (45), 14052–14056. https://doi.org/10.1002/anie.201707401.

(142) Coronado, E.; Dias, J. C.; Giménez-López, M. C.; Giménez-Saiz, C.; Gómez-García, C. J. Synthesis, Structure and Magnetic Characterization of [Fe(Bpp)2][Cu(Pds)2]2·solv (solv=CH3CN and CH3OH). *J. Mol. Struct.* **2008**, *890* (1–3), 215–220. https://doi.org/10.1016/j.molstruc.2008.04.046.

(143) King, P.; Henkelis, J. J.; Kilner, C. A.; Halcrow, M. A. Four New Spin-Crossover Salts of [Fe(3-Bpp)2]2+ (3-Bpp=2,6-Bis[1H-Pyrazol-3-Yl]Pyridine). *Polyhedron* **2013**, *52*, 1449–1456. https://doi.org/10.1016/j.poly.2012.03.038.

(144) Boča, R.; Baran, P.; Dlháň, L.; Fuess, H.; Haase, W.; Renz, F.; Linert, W.; Svoboda, I.; Werner, R. Crystal Structure and Spin Crossover Studies on Bis(2,6-Bis(Benzimidazol-2-Yl)Pyridine) Iron(II) Perchlorate. *Inorganica Chim. Acta* **1997**, *260* (2), 129–136. https://doi.org/10.1016/S0020-1693(96)05550-8.

(145) Boča, R.; Renz, F.; Boča, M.; Fuess, H.; Haase, W.; Kickelbick, G.; Linert, W.; Vrbová-Schikora, M. Tuning the Spin Crossover above Room Temperature: Iron(II) Complexes of Substituted and Deprotonated 2,6-Bis(Benzimidazol-2-Yl)Pyridine. *Inorg. Chem. Commun.* **2005**, *8* (2), 227–230. https://doi.org/10.1016/j.inoche.2004.12.014.

(146) Takahashi, K.; Okai, M.; Mochida, T.; Sakurai, T.; Ohta, H.; Yamamoto, T.; Einaga, Y.; Shiota, Y.; Yoshizawa, K.; Konaka, H.; Sasaki, A. Contribution of Coulomb Interactions to a Two-Step Crystal Structure Phase Transformation Coupled with a Significant Change in Spin Crossover Behavior for

a Series of Charged Fe^{II} Complexes from 2,6-Bis(2-Methylthiazol-4-Yl)Pyridine. *Inorg. Chem.* **2018**, *57* (3), 1277–1287. https://doi.org/10.1021/acs.inorgchem.7b02721.

(147) Ciszek, J. W.; Keane, Z. K.; Cheng, L.; Stewart, M. P.; Yu, L. H.; Natelson, D.; Tour, J. M. Neutral Complexes of First Row Transition Metals Bearing Unbound Thiocyanates and Their Assembly on Metallic Surfaces. *J. Am. Chem. Soc.* **2006**, *128* (10), 3179–3189. https://doi.org/10.1021/ja055459d.

(148) Baker, A.; Goodwin, H.; Rae, A. The Crystal Structure of Bis[2-(Pyridin-2-Ylamino)-4-(Pyridin-2-Yl)Thiazole]Iron(II) Bis(Tetrafluoroborate) Trihydrate. *Aust. J. Chem.* **1984**, *37* (2), 443. https://doi.org/10.1071/CH9840443.

(149) Childs, B. J.; Cadogan, J. M.; Craig, D. C.; Scudder, M. L.; Goodwin, H. A. Electronic and Structural Properties of Iron(II) and Nickel(II) Cationic Complexes of 2-(Pyrazin-2-Ylamino)-4-(Pyridin-2-Yl)Thiazole. *Aust. J. Chem.* **1997**, *50* (2), 129. https://doi.org/10.1071/C96185.

(150) Mishra, V.; Mukherjee, R.; Linares, J.; Balde, C.; Desplanches, C.; Létard, J.-F.; Collet, E.; Toupet, L.; Castro, M.; Varret, F. Temperature-Dependent Interactions and Disorder in the Spin-Transition Compound $[Fe^{II}(L)_2][ClO_4]_2 \cdot C_7H_8$ Through Structural, Calorimetric, Magnetic, Photomagnetic, and Diffuse Reflectance Investigations. *Inorg. Chem.* **2008**, *47* (17), 7577–7587. https://doi.org/10.1021/ic8002977.

(151) Childs, B. J.; Craig, D. C.; Scudder, M. L.; Goodwin, H. A. Structural and Electronic Studies of the Coordination of 6-(Thiazol-2-Yl)-2,2′-Bipyridine and Related Systems to Fe(II), Co(II) and Ni(II). *Inorganica Chim. Acta* **1998**, *274* (1), 32–41. https://doi.org/10.1016/S0020-1693(97)05987-2.

(152) Sugiyarto, K.; Craig, D.; Rae, A.; Goodwin, H. Structural and Electronic Properties of Iron(II) and Nickel(II) Complexes of 2-Triazolyl-1,10-Phenanthroline Derivatives. *Aust. J. Chem.* **1996**, *49* (4), 505. https://doi.org/10.1071/CH9960505.

(153) Sugiyarto, K.; Craig, D.; Goodwin, H. Structural Characterization of Two Crystalline Forms of Bis[2-(1,5-Dimethyltrazol-3-Yl)-1,10-Phenanthroline]-Iron(II) Perchlorate—a Spin Crossover System. *Aust. J. Chem.* **1996**, *49* (4), 497. https://doi.org/10.1071/CH9960497.

(154) Bousseksou, A.; Verelst, M.; Constant-Machado, H.; Lemercier, G.; Tuchagues, J.-P.; Varret, F. $[Fe^{II}(TRIM)_2]F_2$, the First Example of Spin Conversion Monitored by Molecular Vibrations. *Inorg. Chem.* **1996**, *35* (1), 110–115. https://doi.org/10.1021/ic9506567.

(155) Lemercier, G.; Bréfuel, N.; Shova, S.; Wolny, J. A.; Dahan, F.; Verelst, M.; Paulsen, H.; Trautwein, A. X.; Tuchagues, J. A Range of Spin-Crossover Temperature $T_{1/2}$ >300 K Results from Out-of-Sphere Anion Exchange in a Series of Ferrous Materials Based on the 4-(4-Imidazolylmethyl)-2-(2-imidazolylmethyl)Imidazole (Trim) Ligand, $[Fe(Trim)_2]X_2$ (X=F, Cl, Br, I): Comparison of Experimental Results with Those Derived from Density Functional Theory Calculations. *Chem. – Eur. J.* **2006**, *12* (28), 7421–7432. https://doi.org/10.1002/chem.200501249.

(156) Akiyoshi, R.; Hirota, Y.; Kosumi, D.; Tsutsumi, M.; Nakamura, M.; Lindoy, L. F.; Hayami, S. Ferroelectric Metallomesogens Composed of Achiral Spin Crossover Molecules. *Chem. Sci.* **2019**, *10* (22), 5843–5848. https://doi.org/10.1039/C9SC01229J.

(157) Rajnák, C.; Titiš, J.; Fuhr, O.; Ruben, M.; Boča, R. Low Spin Fe(II) Complexes Formed of Monosubstitued 2,6-Bis(2-Benzimidazolyl)Pyridine Ligands. *Polyhedron* **2017**, *123*, 122–131. https://doi.org/10.1016/j.poly.2016.11.009.

(158) Barrios, L. A.; Bartual-Murgui, C.; Peyrecave-Lleixà, E.; Le Guennic, B.; Teat, S. J.; Roubeau, O.; Aromí, G. Homoleptic versus Heteroleptic Formation of Mononuclear Fe(II) Complexes with Tris-Imine Ligands. *Inorg. Chem.* **2016**, *55* (9), 4110–4116. https://doi.org/10.1021/acs.inorgchem.5b02058.

(159) Bartual-Murgui, C.; Diego, R.; Vela, S.; Teat, S. J.; Roubeau, O.; Aromí, G. A Spin-Crossover Molecular Material Describing Four Distinct Thermal Pathways. *Inorg. Chem.* **2018**, *57* (17), 11019–11026. https://doi.org/10.1021/acs.inorgchem.8b01625.

(160) Okai, M.; Takahashi, K.; Sakurai, T.; Ohta, H.; Yamamoto, T.; Einaga, Y. Novel Fe(II) Spin Crossover Complexes Involving a Chalcogen-Bond and π-Stacking Interactions with a Paramagnetic and Nonmagnetic $M(Dmit)_2$ Anion (M = Ni, Au; Dmit = 4,5-Dithiolato-1,3-Dithiole-2-Thione). *J. Mater. Chem. C* **2015**, *3* (30), 7858–7864. https://doi.org/10.1039/C5TC00859J.

(161) Sugiyarto, K.; Craig, D.; Rae, A.; Goodwin, H. Structural and Electronic Properties of Iron(II) and Nickel(II) Complexes of 2,6-Bis(Triazol-3-Yl)Pyridines. *Aust. J. Chem.* **1993**, *46* (8), 1269. https://doi.org/10.1071/CH9931269.

(162) Seredyuk, M.; Znovjyak, K. O.; Kusz, J.; Nowak, M.; Muñoz, M. C.; Real, J. A. Control of the Spin State by Charge and Ligand Substitution: Two-Step Spin Crossover Behaviour in a Novel Neutral Iron(II) Complex. *Dalton Trans*

2014, *43* (43), 16387–16394. https://doi.org/10.1039/C4DT01885K.
(163) Childs, B. J.; Craig, D. C.; Scudder, M. L.; Goodwin, H. A. Coordination of the Strong Field Terimine System 6-Triazol-3-Yl-2,2′-Bipyridine and Substituted Derivatives. Electronic and Structural Properties of Bis(Ligand)Iron(II) Complexes. *Aust. J. Chem.* **1998**, *51* (10), 895. https://doi.org/10.1071/C97202.
(164) Childs, B. J.; Scudder, M. L.; Craig, D. C.; Goodwin, H. A. Structural and Electronic Properties of the Iron(II) and Nickel(II) Bis(Ligand) Complexes of 6-(5-Methyl-1,2,4-Oxadiazol-3-Yl)-2,2′-Bipyridine— a Terpyridine-Based Tridentate. *Aust. J. Chem.* **1999**, *52* (7), 673. https://doi.org/10.1071/CH98153.
(165) Zhang, W.; Zhao, F.; Liu, T.; Yuan, M.; Wang, Z.-M.; Gao, S. Spin Crossover in a Series of Iron(II) Complexes of 2-(2-Alkyl-2 *H* -Tetrazol-5-Yl)-1,10-Phenanthroline: Effects of Alkyl Side Chain, Solvent, and Anion. *Inorg. Chem.* **2007**, *46* (7), 2541–2555. https://doi.org/10.1021/ic062062h.
(166) Zhu, Y.-Y.; Liu, C.-W.; Yin, J.; Meng, Z.-S.; Yang, Q.; Wang, J.; Liu, T.; Gao, S. Structural Phase Transition in a Multi-Induced Mononuclear Fe^{II} Spin-Crossover Complex. *Dalton Trans.* **2015**, *44* (48), 20906–20912. https://doi.org/10.1039/C5DT03216D.
(167) Wang, Y.-Q.; Pan, Y.; Gao, W.-Q.; Wu, Y.; Liu, C.-H.; Zhu, Y.-Y. Construction of Optical Active Metallo-Supramolecular Polymers from Enantiopure Bis-Pybox Ligands. *Tetrahedron* **2019**, *75* (28), 3809–3814. https://doi.org/10.1016/j.tet.2019.04.023.
(168) Zhu, Y.-Y.; Li, H.-Q.; Ding, Z.-Y.; Lü, X.-J.; Zhao, L.; Meng, Y.-S.; Liu, T.; Gao, S. Spin Transitions in a Series of $[Fe(Pybox)_2]^{2+}$ Complexes Modulated by Ligand Structures, Counter Anions, and Solvents. *Inorg. Chem. Front.* **2016**, *3* (12), 1624–1636. https://doi.org/10.1039/C6QI00417B.
(169) Barrios, L. A.; Peyrecave-Lleixà, E.; Craig, G. A.; Roubeau, O.; Teat, S. J.; Aromí, G. Unusual Crystal Packing in a Family of $[Fe\{2,6\text{-bis(Pyrazol-3-yl)Pyridine}\}_2]^{2+}$ Compounds and the Effect on the Occurrence of Spin Crossover and Its Cooperative Character. *Eur. J. Inorg. Chem.* **2014**, *2014* (35), 6013–6021. https://doi.org/10.1002/ejic.201403009.
(170) Clemente-León, M.; Coronado, E.; Giménez-López, M. C.; Romero, F. M. Structural, Thermal, and Magnetic Study of Solvation Processes in Spin-Crossover $[Fe(Bpp)_2][Cr(L)(Ox)_2]_2 \cdot n\,H_2O$ Complexes. *Inorg. Chem.* **2007**, *46* (26), 11266–11276. https://doi.org/10.1021/ic700910n.
(171) Shiga, T.; Saiki, R.; Akiyama, L.; Kumai, R.; Natke, D.; Renz, F.;

Cameron, J. M.; Newton, G. N.; Oshio, H. A Brønsted-Ligand-Based Iron Complex as a Molecular Switch with Five Accessible States. *Angew. Chem. Int. Ed.* **2019**, *58* (17), 5658–5662. https://doi.org/10.1002/anie.201900909.

(172) Halcrow, M. A.; Capel Berdiell, I.; Pask, C. M.; Kulmaczewski, R. Relationship between the Molecular Structure and Switching Temperature in a Library of Spin-Crossover Molecular Materials. *Inorg. Chem.* **2019**, *58* (15), 9811–9821. https://doi.org/10.1021/acs.inorgchem.9b00843.

(173) Craig, G. A.; Sánchez Costa, J.; Roubeau, O.; Teat, S. J.; Aromí, G. Coupled Crystallographic Order–Disorder and Spin State in a Bistable Molecule: Multiple Transition Dynamics. *Chem. – Eur. J.* **2011**, *17* (11), 3120–3127. https://doi.org/10.1002/chem.201003197.

(174) Roberts, T. D.; Tuna, F.; Malkin, T. L.; Kilner, C. A.; Halcrow, M. A. An Iron(II) Complex Exhibiting Five Anhydrous Phases, Two of Which Interconvert by Spin-Crossover with Wide Hysteresis. *Chem Sci* **2012**, *3* (2), 349–354. https://doi.org/10.1039/C1SC00584G.

(175) De Bruin, B.; Bill, E.; Bothe, E.; Weyhermüller, T.; Wieghardt, K. Molecular and Electronic Structures of Bis(Pyridine-2,6-Diimine)Metal Complexes $[ML_2](PF_6)_n$ (*n* = 0, 1, 2, 3; M = Mn, Fe, Co, Ni, Cu, Zn). *Inorg. Chem.* **2000**, *39* (13), 2936–2947. https://doi.org/10.1021/ic000113j.

(176) Saiki, R.; Miyamoto, H.; Sagayama, H.; Kumai, R.; Newton, G. N.; Shiga, T.; Oshio, H. Substituent Dependence on the Spin Crossover Behaviour of Mononuclear Fe(II) Complexes with Asymmetric Tridentate Ligands. *Dalton Trans.* **2019**, *48* (10), 3231–3236. https://doi.org/10.1039/C9DT00204A.

(177) Roberts, T. D.; Little, M. A.; Kershaw Cook, L. J.; Barrett, S. A.; Tuna, F.; Halcrow, M. A. Iron(II) Complexes of 2,6-Di(1-Alkylpyrazol-3-Yl)Pyridine Derivatives – The Influence of Distal Substituents on the Spin State of the Iron Centre. *Polyhedron* **2013**, *64*, 4–12. https://doi.org/10.1016/j.poly.2013.01.057.

(178) Craig, G. A.; Costa, J. S.; Roubeau, O.; Teat, S. J.; Aromí, G. An Fe^{II} Spin-Crossover Complex Becomes Increasingly Cooperative with Ageing. *Eur. J. Inorg. Chem.* **2013**, *2013* (5–6), 745–752. https://doi.org/10.1002/ejic.201201041.

(179) Nikovskiy, I.; Polezhaev, A.; Novikov, V.; Aleshin, D.; Pavlov, A.; Saffiulina, E.; Aysin, R.; Dorovatovskii, P.; Nodaraki, L.; Tuna, F.; Nelyubina, Y. Towards the Molecular Design of Spin-Crossover Complexes of 2,6-Bis(Pyrazol-3-yl)Pyridines. *Chem. – Eur. J.* **2020**, *26* (25), 5629–5638. https://doi.org/10.1002/chem.202000047.

(180) Pan, Y.; Meng, Y.-S.; Liu, Q.; Gao, W.-Q.; Liu, C.-H.; Liu, T.; Zhu, Y.-

Y. Construction of SCO-Active Fe(II) Mononuclear Complexes from the Thio-Pybox Ligand. *Inorg. Chem.* **2020**, *59* (11), 7398–7407. https://doi.org/10.1021/acs.inorgchem.9b03506.

(181) Blakesley, D. W.; Payne, S. C.; Hagen, K. S. Spin-State Variation in Solid State and Solution of Mononuclear Iron(II) 1,4,7-Trimethyl-1,4,7-Triazacyclonane Complexes. *Inorg. Chem.* **2000**, *39* (9), 1979–1989. https://doi.org/10.1021/ic990584+.

(182) Shongwe, M. S.; Al-Zaabi, U. A.; Al-Mjeni, F.; Eribal, C. S.; Sinn, E.; Al-Omari, I. A.; Hamdeh, H. H.; Matoga, D.; Adams, H.; Morris, M. J.; Rheingold, A. L.; Bill, E.; Sellmyer, D. J. Accessibility and Selective Stabilization of the Principal Spin States of Iron by Pyridyl versus Phenolic Ketimines: Modulation of the$^6A_1 \leftrightarrow ^2T_2$ Ground-State Transformation of the $[FeN_4O_2]^+$ Chromophore. *Inorg. Chem.* **2012**, *51* (15), 8241–8253. https://doi.org/10.1021/ic300732r.

(183) Glijer, D.; Hébert, J.; Trzop, E.; Collet, E.; Toupet, L.; Cailleau, H.; Matouzenko, G. S.; Lazar, H. Z.; Létard, J. F.; Koshihara, S.; Buron-Le Cointe, M. Photoinduced Phenomena and Structural Analysis Associated with the Spin-State Switching in the [Fe II (DPEA) (NCS) 2] Complex. *Phys. Rev. B* **2008**, *78* (13), 134112. https://doi.org/10.1103/PhysRevB.78.134112.

(184) Matouzenko, G. S.; Bousseksou, A.; Lecocq, S.; Van Koningsbruggen, P. J.; Perrin, M.; Kahn, O.; Collet, A. Polymorphism in Spin Transition Systems. Crystal Structure, Magnetic Properties, and Mössbauer Spectroscopy of Three Polymorphic Modifications of $[Fe(DPPA)(NCS)_2]$ [DPPA = (3-Aminopropyl)Bis(2-Pyridylmethyl)Amine]. *Inorg. Chem.* **1997**, *36* (25), 5869–5879. https://doi.org/10.1021/ic971174t.

(185) Yu, F.; Li, B. Pressure-Driven Thermal Spin Transition Behaviors in Mono-Nuclear Iron(II) Compounds: Synthesis, Crystal Structure and Magnetic Properties. *Inorg. Chem. Commun.* **2011**, *14* (9), 1452–1455. https://doi.org/10.1016/j.inoche.2011.05.045.

(186) Matouzenko, G. S.; Létard, J.-F.; Lecocq, S.; Bousseksou, A.; Capes, L.; Salmon, L.; Perrin, M.; Kahn, O.; Collet, A. Two-Step Spin Crossover in a Mononuclear Compound [Fe(DPEA)(bim)](ClO4)2·0.5 H2O[DPEA = (2-Aminoethyl)bis(2-pyridylmethyl)amine, bim = 2,2-Bisimidazole] − Crystal Structure, Magnetic Properties, Mössbauer Spectroscopy, and Photomagnetic Effects. *Eur. J. Inorg. Chem.* **2001**, *2001* (11), 2935–2945. https://doi.org/10.1002/1099-0682(200111)2001:11%253C2935::AID-EJIC2935%253E3.0.CO;2-D.

(187) Matouzenko, G. S.; Bousseksou, A.; Borshch, S. A.; Perrin, M.; Zein, S.; Salmon, L.; Molnar, G.; Lecocq, S. Cooperative Spin Crossover and Order−Disorder Phenomena in a Mononuclear Compound [Fe(DAPP)(Abpt)](ClO_4)$_2$ [DAPP = [Bis(3-Aminopropyl)(2-Pyridylmethyl)Amine], Abpt = 4-Amino-3,5-Bis(Pyridin-2-Yl)-1,2,4-Triazole]. *Inorg. Chem.* **2004**, *43* (1), 227–236. https://doi.org/10.1021/ic034450e.

(188) Létard, J.; Asthana, S.; Shepherd, H. J.; Guionneau, P.; Goeta, A. E.; Suemura, N.; Ishikawa, R.; Kaizaki, S. Photomagnetism of a *Sym-cis* -Dithiocyanato Iron(II) Complex with a Tetradentate *N* , *N* ′-Bis(2-pyridylmethyl)1,2-ethanediamine Ligand. *Chem. – Eur. J.* **2012**, *18* (19), 5924–5934. https://doi.org/10.1002/chem.201102637.

(189) Zhou, J.; Zhu, B.-W.; Luan, J.; Liu, Z.; Fang, J.-K.; Bao, X.; Peng, G.; Tucek, J.; Bao, S.-S.; Zheng, L.-M. In Air a Spin Crossover Active Iron(II) Complex of Amine/$NCBH_3^-$ Ligands Is Converted to a Low Spin Complex of Imine/CN^- Ligands. *Dalton Trans.* **2015**, *44* (47), 20551–20561. https://doi.org/10.1039/C5DT03464G.

(190) Wei, R.-J.; Tao, J.; Huang, R.-B.; Zheng, L.-S. Reversible and Irreversible Vapor-Induced Guest Molecule Exchange in Spin-Crossover Compounds. *Inorg. Chem.* **2011**, *50* (17), 8553–8564. https://doi.org/10.1021/ic201142t.

(191) Yu, F. Magnetic Properties of a Mononuclear Iron(II) Complex with a Typical FeN_6 Coordination Octahedron. *Acta Crystallogr. C* **2012**, *68* (10), m287–m290. https://doi.org/10.1107/S010827011203209X.

(192) Li, B.; Wei, R.-J.; Tao, J.; Huang, R.-B.; Zheng, L.-S.; Zheng, Z. Solvent-Induced Transformation of Single Crystals of a Spin-Crossover (SCO) Compound to Single Crystals with Two Distinct SCO Centers. *J. Am. Chem. Soc.* **2010**, *132* (5), 1558–1566. https://doi.org/10.1021/ja909695f.

(193) Wei, R.-J.; Li, B.; Tao, J.; Huang, R.-B.; Zheng, L.-S.; Zheng, Z. Making Spin-Crossover Crystals by Successive Polymorphic Transformations. *Inorg. Chem.* **2011**, *50* (4), 1170–1172. https://doi.org/10.1021/ic102231j.

(194) Li, B.; Wei, R.-J.; Tao, J.; Huang, R.-B.; Zheng, L.-S. Pressure Effects on a Spin-Crossover Monomeric Compound [Fe(Pmea)(SCN)$_2$] (Pmea = Bis[(2-Pyridyl)Methyl]-2-(2-Pyridyl)Ethylamine). *Inorg. Chem.* **2010**, *49* (2), 745–751. https://doi.org/10.1021/ic902161v.

(195) Kisslinger, S.; Kelm, H.; Zheng, S.; Beitat, A.; Würtele, C.; Wortmann, R.; Bonnet, S.; Herres-Pawlis, S.; Krüger, H.; Schindler, S. Synthesis and

Characterization of Iron(II) Thiocyanate Complexes with Derivatives of the Tris(Pyridine-2-ylmethyl)Amine (Tmpa) Ligand. *Z. Für Anorg. Allg. Chem.* **2012**, *638* (12–13), 2069–2077. https://doi.org/10.1002/zaac.201200237.

(196) Sun, X.-P.; Wei, R.-J.; Yao, Z.-S.; Tao, J. Solvent Effects on the Structural Packing and Spin-Crossover Properties of a Mononuclear Iron(II) Complex. *Cryst. Growth Des.* **2018**, *18* (11), 6853–6862. https://doi.org/10.1021/acs.cgd.8b01079.

(197) Chen, X.-Q.; Cai, Y.-D.; Ye, Y.-S.; Tong, M.-L.; Bao, X. Investigation of SCO Property–Structural Relationships in a Family of Mononuclear Fe(II) Complexes. *Inorg. Chem. Front.* **2019**, *6* (8), 2194–2199. https://doi.org/10.1039/C9QI00577C.

(198) Spiccia, L.; Fallon, G. D.; Grannas, M. J.; Nichols, P. J.; Tiekink, E. R. T. Synthesis and Characterisation of Mononuclear and Binuclear Iron(II) Complexes of Pentadentate and Bis(Pentadentate) Ligands Derived from 1,4,7-Triazacyclononane. *Inorganica Chim. Acta* **1998**, *279* (2), 192–199. https://doi.org/10.1016/S0020-1693(98)00122-4.

(199) Ortega-Villar, N.; Ugalde-Saldívar, V. M.; Muñoz, M. C.; Ortiz-Frade, L. A.; Alvarado-Rodríguez, J. G.; Real, J. A.; Moreno-Esparza, R. Synthesis and Relative Stability of a Series of Compounds of Type $[Fe(II)(Bztpen)X]^+$, Where Bztpen = Pentadentate Ligand, N_5 , and X^- = Monodentate Anion. *Inorg. Chem.* **2007**, *46* (18), 7285–7293. https://doi.org/10.1021/ic0620743.

(200) Fei, B.; Chen, X. Q.; Cai, Y. D.; Fang, J.-K.; Tong, M. L.; Tucek, J.; Bao, X. The Influence of NCE^- (E = S, Se, BH_3) Ligands on the Temperature of Spin Crossover in a Family of Iron(II) Mononuclear Complexes. *Inorg. Chem. Front.* **2018**, *5* (7), 1671–1676. https://doi.org/10.1039/C8QI00303C.

(201) Ma, T.-T.; Sun, X.-P.; Yao, Z.-S.; Tao, J. Homochiral *versus* Racemic Polymorphs of Spin-Crossover Iron(II) Complexes with Reversible LIESST Effect. *Inorg. Chem. Front.* **2020**, *7* (5), 1196–1204. https://doi.org/10.1039/C9QI01590F.

(202) Luan, J.; Zhou, J.; Liu, Z.; Zhu, B.; Wang, H.; Bao, X.; Liu, W.; Tong, M.-L.; Peng, G.; Peng, H.; Salmon, L.; Bousseksou, A. Polymorphism-Dependent Spin-Crossover: Hysteretic Two-Step Spin Transition with an Ordered [HS–HS–LS] Intermediate Phase. *Inorg. Chem.* **2015**, *54* (11), 5145–5147. https://doi.org/10.1021/acs.inorgchem.5b00629.

(203) El Hajj, F.; Sebki, G.; Patinec, V.; Marchivie, M.; Triki, S.; Handel, H.; Yefsah, S.; Tripier, R.; Gómez-García, C. J.; Coronado, E. Macrocycle-Based

Spin-Crossover Materials. *Inorg. Chem.* **2009**, *48* (21), 10416–10423. https://doi.org/10.1021/ic9012476.

(204) Milin, E.; Benaicha, B.; El Hajj, F.; Patinec, V.; Triki, S.; Marchivie, M.; Gómez-García, C. J.; Pillet, S. Magnetic Bistability in Macrocycle-Based Fe^{II} Spin-Crossover Complexes: Counter Ion and Solvent Effects. *Eur. J. Inorg. Chem.* **2016**, *2016* (34), 5305–5314. https://doi.org/10.1002/ejic.201600660.

(205) Drahoš, B.; Trávníček, Z. Spin Crossover Fe(II) Complexes of a Cross-Bridged Cyclam Derivative. *Dalton Trans.* **2018**, *47* (17), 6134–6145. https://doi.org/10.1039/C8DT00414E.

(206) Ortega-Villar, N.; Thompson, A. L.; Muñoz, M. C.; Ugalde-Saldívar, V. M.; Goeta, A. E.; Moreno-Esparza, R.; Real, J. A. Solid- and Solution-State Studies of the Novel μ-Dicyanamide-Bridged Dinuclear Spin-Crossover System $\{[(Fe(Bztpen)]_2\ [\mu\text{-}N(CN)_2\ ]\}(PF_6\ )_3 \cdot n\ H_2\ O$. *Chem. – Eur. J.* **2005**, *11* (19), 5721–5734. https://doi.org/10.1002/chem.200500171.

(207) Chang, H. R.; McCusker, J. K.; Toftlund, H.; Wilson, S. R.; Trautwein, A. X.; Winkler, H.; Hendrickson, D. N. [Tetrakis(2-Pyridylmethyl)Ethylenediamine]Iron(II) Perchlorate, the First Rapidly Interconverting Ferrous Spin-Crossover Complex. *J. Am. Chem. Soc.* **1990**, *112* (19), 6814–6827. https://doi.org/10.1021/ja00175a012.

(208) McCusker, J. K.; Toftlund, H.; Rheingold, A. L.; Hendrickson, D. N. Ligand Conformational Changes Affecting 5T2 .Fwdarw. 1A1 Intersystem Crossing in a Ferrous Complex. *J. Am. Chem. Soc.* **1993**, *115* (5), 1797–1804. https://doi.org/10.1021/ja00058a026.

(209) Matouzenko, G. S.; Luneau, D.; Molnár, G.; Ould-Moussa, N.; Zein, S.; Borshch, S. A.; Bousseksou, A.; Averseng, F. A Two-Step Spin Transition and Order–Disorder Phenomena in the Mononuclear Compound [Fe(Hpy-DAPP)]$(BF_4\ )_2$. *Eur. J. Inorg. Chem.* **2006**, *2006* (13), 2671–2682. https://doi.org/10.1002/ejic.200600068.

(210) Deeney, F. A.; Harding, C. J.; Morgan, G. G.; McKee, V.; Nelson, J.; Teat, S. J.; Clegg, W. Response to Steric Constraint in Azacryptate and Related Complexes of Iron-(II) and -(III) *. *J. Chem. Soc. Dalton Trans.* **1998**, No. 11, 1837–1844. https://doi.org/10.1039/a801046c.

(211) Christiansen, L.; Hendrickson, D. N.; Toftlund, H.; Wilson, S. R.; Xie, C. L. Synthesis and Structure of Metal Complexes of Triaza Macrocycles with Three Pendant Pyridylmethyl Arms. *Inorg. Chem.* **1986**, *25* (16), 2813–2818. https://doi.org/10.1021/ic00236a031.

(212) Sinha, S.; Das, S.; Sikari, R.; Parua, S.; Brandaõ, P.; Demeshko, S.; Meyer, F.; Paul, N. D. Redox Noninnocent Azo-Aromatic Pincers and Their Iron Complexes. Isolation, Characterization, and Catalytic Alcohol Oxidation. *Inorg. Chem.* **2017**, *56* (22), 14084–14100. https://doi.org/10.1021/acs.inorgchem.7b02238.

(213) Soliman, A. A.; Khattab, M. M.; Reissner, M.; Weinberger, P.; Werner, F.; Linert, W. Synthesis, Structure, Spectroscopic and Magnetic Characterization of a Novel Spin-Crossover Iron(II) Complex with 1-Cyclopropyltetrazole Ligands. *Inorganica Chim. Acta* **2007**, *360* (14), 3987–3996. https://doi.org/10.1016/j.ica.2007.05.048.

(214) Hassan, N.; Weinberger, P.; Mereiter, K.; Werner, F.; Molnar, G.; Bousseksou, A.; Valtiner, M.; Linert, W. Comparative Investigations on a Series of [Hexakis(1-(Tetrazol-1-Yl)Alkane-N4)Iron(II)] Bis(Tetrafluoroborate) Spin Crossover Complexes: Methyl- to Butyl-Substituted Species. *Inorganica Chim. Acta* **2008**, *361* (5), 1291–1297. https://doi.org/10.1016/j.ica.2007.08.023.

(215) Müller, D.; Knoll, C.; Seifried, M.; Welch, J. M.; Giester, G.; Reissner, M.; Weinberger, P. Halogenated Alkyltetrazoles for the Rational Design of Fe^{II} Spin-Crossover Materials: Fine-Tuning of the Ligand Size. *Chem. – Eur. J.* **2018**, *24* (20), 5271–5280. https://doi.org/10.1002/chem.201704656.

(216) Tafili-Kryeziu, M.; Weil, M.; Muranaka, T.; Bousseksou, A.; Hasegawa, M.; Jun, A.; Linert, W. Effect of the Counter-Anion on the Spin-Transition Properties of a Family of Fe(Ii) Tetrazole Complexes, [Fe(I4tz)6]X2 (X = ClO4−, PF6−, SbF6−, BF4−). *Dalton Trans.* **2013**, *42* (44), 15796. https://doi.org/10.1039/c3dt52339j.

(217) Gütlich, P.; Gaspar, A. B.; Garcia, Y. Spin State Switching in Iron Coordination Compounds. *Beilstein J. Org. Chem.* **2013**, *9*, 342–391. https://doi.org/10.3762/bjoc.9.39.

(218) Ishikawa, R.; Matsumoto, K.; Onishi, K.; Kubo, T.; Fuyuhiro, A.; Hayami, S.; Inoue, K.; Kaizaki, S.; Kawata, S. Magnetic Properties of Iron(II) and Cobalt(II) Complexes of Tetrakis(2-Pyridyl)Methane. Spin-Crossover Behavior in the Cobalt(II) Complex. *Chem. Lett.* **2009**, *38* (6), 620–621. https://doi.org/10.1246/cl.2009.620.

(219) Lee, Y. H.; Kato, K.; Kubota, E.; Kawata, S.; Hayami, S. Molecular Structure and Magnetic Properties of Pentapyridyl-Based Iron(II) and Cobalt(II) Complexes. *Chem. Lett.* **2012**, *41* (6), 620–621. https://doi.org/10.1246/cl.2012.620.

(220) Nihei, M.; Han, L.; Oshio, H. Magnetic Bistability and Single-Crystal-to-Single-Crystal Transformation Induced by Guest Desorption. *J. Am. Chem. Soc.* **2007**, *129* (17), 5312–5313. https://doi.org/10.1021/ja069120i.

(221) Elhaïk, J.; Kilner, C. A.; Halcrow, M. A. An Iron(II) Complex Salt That Crystallises in Three Crystal Forms, One of Which Undergoes a Sterically Controlled Incomplete Spin-State Transition on Cooling. *CrystEngComm* **2005**, *7* (23), 151–157. https://doi.org/10.1039/B417718E.

(222) Nihei, M.; Han, L.; Tahira, H.; Oshio, H. Syntheses, Structures and Magnetic Properties of Iron(II) Complexes with Bulky Tridentate Ligands. *Inorganica Chim. Acta* **2008**, *361* (14–15), 3926–3930. https://doi.org/10.1016/j.ica.2008.03.118.

(223) Pritchard, R.; Kilner, C. A.; Halcrow, M. A. Unexpected Product Distributions in the Synthesis of 2,6-Bis-(Indazolyl)Pyridine and 2-(Pyrazol-1-Yl)-6-(Indazolyl)Pyridine. *Tetrahedron Lett.* **2009**, *50* (21), 2484–2486. https://doi.org/10.1016/j.tetlet.2009.03.035.

(224) Halcrow, M. A. The Synthesis and Coordination Chemistry of 2,6-Bis(Pyrazolyl)Pyridines and Related Ligands — Versatile Terpyridine Analogues. *Coord. Chem. Rev.* **2005**, *249* (24), 2880–2908. https://doi.org/10.1016/j.ccr.2005.03.010.

(225) Holland, J. M.; Barrett, S. A.; Kilner, C. A.; Halcrow, M. A. Control of the Spin State of Fe(II) 2,6-Di(Pyrazol-1-Yl)Pyridine Complexes by Distal Ligand Substitution. *Inorg. Chem. Commun.* **2002**, *5* (5), 328–332. https://doi.org/10.1016/S1387-7003(02)00398-2.

(226) Pelascini, F.; Wesolek, M.; Peruch, F.; Cian, A. D.; Kyritsakas, N.; Lutz, P. J.; Kress, J. Iron Complexes of Terdentate Nitrogen Ligands: Formation and X-Ray Structure of Three New Dicationic Complexes. *Polyhedron* **2004**, *23* (18), 3193–3199. https://doi.org/10.1016/j.poly.2004.10.001.

(227) Pritchard, R.; Kilner, C. A.; Barrett, S. A.; Halcrow, M. A. Two New 4′,4″-Disubstituted Dipyrazolylpyridine Derivatives, and the Structures and Spin States of Their Iron(II) Complexes. *Inorganica Chim. Acta* **2009**, *362* (12), 4365–4371. https://doi.org/10.1016/j.ica.2009.01.022.

(228) Eichhorn, D. M.; Armstrong, W. H. M{Hydrotris(3-Phenylpyrazol-1-Yl)Borate}2: Sterically Encumbered Iron(II) and Manganese(II) Complexes. *Inorg. Chem.* **1990**, *29* (18), 3607–3612. https://doi.org/10.1021/ic00343a058.

(229) Salmon, L.; Molnár, G.; Cobo, S.; Oulié, P.; Etienne, M.; Mahfoud, T.; Demont, P.; Eguchi, A.; Watanabe, H.; Tanaka, K.; Bousseksou, A. Re-

Investigation of the Spin Crossover Phenomenon in the Ferrous Complex [Fe(HB(Pz)3)2]. *New J. Chem.* **2009**, *33* (6), 1283. https://doi.org/10.1039/b902811k.

(230) Sohrin, Y.; Kokusen, H.; Matsui, M. Control of Ligand Field Strength through Intra- and Interligand Contact. Octahedral Iron(II) Poly(Pyrazolyl)Borate Complexes. *Inorg. Chem.* **1995**, *34* (15), 3928–3934. https://doi.org/10.1021/ic00119a014.

(231) Janiak, C.; Temizdemir, S.; Dechert, S.; Deck, W.; Girgsdies, F.; Heinze, J.; Kolm, M.; Scharmann, T.; Zipffel, O. Binary [Hydrotris(Indazol-1-Yl)Borato]Metal Complexes, M(Tp4Bo)2[1] with M = Fe, Co, Ni, Cu, and Zn: Electronic Properties and Solvent-Dependent Framework Structures through C−H···π Interactions. *Eur. J. Inorg. Chem.* **2000**, *2000* (6), 1229–1241. https://doi.org/10.1002/(SICI)1099-0682(200006)2000:6%253C1229::AID-EJIC1229%253E3.3.CO;2-G.

(232) Morgenstern-Badarau, I.; Lambert, F.; Philippe Renault, J.; Cesario, M.; Maréchal, J.-D.; Maseras, F. Amine Conformational Change and Spin Conversion Induced by Metal-Assisted Ligand Oxidation: From the Seven-Coordinate Iron(II)–TPAA Complex to the Two Oxidized Iron(II)–(Py)3tren Isomers. Characterization, Crystal Structures, and Density Functional Study. *Inorganica Chim. Acta* **2000**, *297* (1–2), 338–350. https://doi.org/10.1016/S0020-1693(99)00363-1.

(233) Struch, N.; Topić, F.; Schnakenburg, G.; Rissanen, K.; Lützen, A. Electron-Deficient Pyridylimines: Versatile Building Blocks for Functional Metallosupramolecular Chemistry. *Inorg. Chem.* **2018**, *57* (1), 241–250. https://doi.org/10.1021/acs.inorgchem.7b02412.

(234) Brewer, G.; Luckett, C.; May, L.; Beatty, A. M.; Scheidt, W. R. Synthesis and Characterization of Tripodal Iron(II) Complexes Prepared from 2-Pyridinecarboxaldehyde and 1-Methyl-2-Imidazolecarboxaldehyde: Stabilization of Iron(II) Cations with N6 Donor Sets. *Inorganica Chim. Acta* **2004**, *357* (8), 2390–2396. https://doi.org/10.1016/j.ica.2004.01.007.

(235) Boubekeur, K.; Deroche, A.; Lambert, F.; Morgenstern-Badarau, I. {Tris[4-(2-Pyridyl)-3-Aza-3-Butenyl]Amine}iron(II) Bis(Hexafluorophosphate), [Fe(Py3tren)](PF6)2. *Acta Crystallogr. C* **1995**, *51* (11), 2244–2246. https://doi.org/10.1107/S010827019500597X.

(236) Brewer, G.; Butcher, R. J.; Viragh, C.; White, G. Supramolecular Assemblies Prepared from an Iron(Ii) Tripodal Imidazole Complex. A Molecular

Scaffolding for the Self Assembly of Icosahedral Complexes of K+, Rb+, Cs+ and NH4+ Cations. *Dalton Trans.* **2007**, No. 37, 4132. https://doi.org/10.1039/b704823h.

(237) Doedens, R. J.; Dahl, L. F. Structure of the Hexapyridineiron (II) Salt of the Tetranuclear Iron Carbonyl Anion, $[Fe_4 (CO)_{13}]^{-2}$, with Comments Concerning the Nonisolation of the Corresponding Neutral Tetranuclear Iron Carbonyl, $Fe_4 (CO)_{14}$. *J. Am. Chem. Soc.* **1966**, *88* (21), 4847–4855. https://doi.org/10.1021/ja00973a018.

(238) Aparici Plaza, L.; Baranowska, K.; Becker, B. Hexakis(1-Methylimidazole-κ N^3)Iron(II) Dichloride Dihydrate. *Acta Crystallogr. Sect. E Struct. Rep. Online* **2006**, *62* (9), m2077–m2079. https://doi.org/10.1107/S160053680603042X.

(239) Katz, B. A.; Strouse, C. E. Spin-State Isomerism of Tris(2-Picolylamine)Iron(II). The Diiodide and the Hydrated Dichloride. *Inorg. Chem.* **1980**, *19* (3), 658–665. https://doi.org/10.1021/ic50205a017.

(240) Mochida, Naotaka; Kimura, Akifumi; Ishida, Takayuki. CCDC 1434211: Experimental Crystal Structure Determination, 2015. https://doi.org/10.5517/CC1K4DWZ.

(241) Healy, P.; Skelton, B.; White, A. Structural Studies in the Iron(III)/Chloride/α,α'-Diimine System. III. Crystal Structures of Tris(2,2'-Bipyridine)Iron(II) μ-Oxo-Bis[Trichloroferrate(III)] and of "Compound A", μ-Oxo-Bis[Cis-Chlorobis(1 ,10-Phenanthroline)Iron(III)] Chloride Solvate. *Aust. J. Chem.* **1983**, *36* (10), 2057. https://doi.org/10.1071/CH9832057.

(242) Heilmann, J.; Lerner, H.-W.; Bolte, M. Tris(2,2′-Bipyridyl)Iron(II) Dibromide 4.5-Hydrate. *Acta Crystallogr. Sect. E Struct. Rep. Online* **2006**, *62* (7), m1477–m1478. https://doi.org/10.1107/S1600536806020903.

(243) Huang, W.; Qian, H. Synthesis and Single-Crystal Structures of Two Copper(II) Complexes and One Iron(II) Complex Prepared by in Situ Ligand Substitution at Room Temperature. *Transit. Met. Chem.* **2006**, *31* (5), 621–629. https://doi.org/10.1007/s11243-006-0039-8.

(244) Dick, S. Crystal Structure of Tris(2,2'-Bipyridine)Iron(II) Bis(Hexafluorophosphate), (C10H8N2)3Fe(PF6)2. *Z. Für Krist. - New Cryst. Struct.* **1998**, *213* (1–4), 370. https://doi.org/10.1524/ncrs.1998.213.14.370.

(245) Huang, W.; Ogawa, T. Structural and Spectroscopic Characterizations of Low-Spin [Fe(4,4′-Dimethyl-2,2′-Bipyridine)3](NCS)2·3H2O Prepared from High-Spin Iron(II) Dithiocyanate Tetrapyridine. *J. Mol. Struct.* **2006**, *785* (1–3),

21–26. https://doi.org/10.1016/j.molstruc.2005.09.035.

(246) Batten, S. R.; Murray, K. S.; Sinclair, N. J. Tris(2,2′-Bipyridyl- *N*, *N* ′)Iron(II) Diperchlorate. *Acta Crystallogr. C* **2000**, *56* (8), e320–e320. https://doi.org/10.1107/S0108270100009185.

(247) Goodwin, H.; Kucharski, E.; White, A. Crystal Structure of Tris(2-Methyl-1,10-Phenanthroline)Iron(II) Tetraphenylborate. *Aust. J. Chem.* **1983**, *36* (6), 1115. https://doi.org/10.1071/CH9831115.

(248) De Munno, G.; Julve, M.; Real, J. A. Synthesis and Crystal Structure of the Low-Spin Iron(II) Complex [Fe(Bpym)3](ClO4)2·1/4H2O (Bpym=2,2′-Bipyrimidine). *Inorganica Chim. Acta* **1997**, *255* (1), 185–188. https://doi.org/10.1016/S0020-1693(96)05340-6.

(249) Van Albada, G. A.; Smeets, W. J. J.; Spek, A. L.; Reedijk, J. Crystal Structure and Spectroscopy of the Low-Spin Iron(II) Compound: Tris(Bipyrimidine)Iron(II) Bis(Triflate), [Fe(Bipym)33](CF3SO3)2. *J. Chem. Crystallogr.* **2000**, *30* (7), 441–444. https://doi.org/10.1023/A:1011339214337.

(250) Harimanow, L. S.; Sugiyarto, K. H.; Craig, D. C.; Scudder, M. L.; Goodwin, H. A. Magnetic, Spectral and Structural Aspects of Spin Transitions in Iron(II) Complexes of 2-(Pyrazol-3-Yl)Pyridine and 3-(Thiazol-2-Yl)Pyrazole. *Aust. J. Chem.* **1999**, *52* (2), 109. https://doi.org/10.1071/C98083.

(251) Onggo, D.; Scudder, M. L.; Craig, D. C.; Goodwin, H. A. [No Title Found]. *Aust. J. Chem.* **2000**, *53* (2), 153. https://doi.org/10.1071/CH99128.

(252) Smithson, R. J.; Kilner, C. A.; Brough, A. R.; Halcrow, M. A. Iron(II) Complexes of (Pyrazol-3-Yl)Pyrazine. Anion-Dependent Formation of a Hydrogen-Bonded, Chiral Nanoporous Lattice. *Polyhedron* **2003**, *22* (5), 725–733. https://doi.org/10.1016/S0277-5387(02)01403-1.

(253) Ni, Z.; Shores, M. P. Magnetic Observation of Anion Binding in Iron Coordination Complexes: Toward Spin-Switching Chemosensors. *J. Am. Chem. Soc.* **2009**, *131* (1), 32–33. https://doi.org/10.1021/ja807379a.

(254) Jones, L. F.; Kilner, C. A.; Halcrow, M. A. A Trinuclear Iron(III) Compound with an Unusual T-Shaped [Fe3(μ 3-O)]7+ Core. *J. Clust. Sci.* **2010**, *21* (3), 279–290. https://doi.org/10.1007/s10876-010-0283-0.

(255) Lathion, T.; Guénée, L.; Besnard, C.; Bousseksou, A.; Piguet, C. Deciphering the Influence of Meridional versus Facial Isomers in Spin Crossover Complexes. *Chem. – Eur. J.* **2018**, *24* (63), 16873–16888. https://doi.org/10.1002/chem.201804161.

(256) Martak, F.; Onggo, D.; Ismunandar; Nugroho, A. A.; Meetsma, A.

CSD Commun. Priv. Commun. **2009**. https://doi.org/CCDC%2520746927.

(257) Field, L. D.; Messerle, B. A.; Soler, L. P.; Hambley, T. W.; Turner, P. Iron(II) Complexes Containing Poly(1-Pyrazolyl)Methane Ligands. *J. Organomet. Chem.* **2002**, *655* (1–2), 146–157. https://doi.org/10.1016/S0022-328X(02)01471-7.

(258) Kulmaczewski, R.; Halcrow, M. A. Structures and Spin States of Crystalline [Fe(NCS)$_2$ L$_2$] and [FeL$_3$]$^{2+}$ Complexes (L = an Annelated 1,10-Phenanthroline Derivative). *CrystEngComm* **2016**, *18* (14), 2570–2578. https://doi.org/10.1039/C6CE00163G.

(259) Vittaya, L.; Leesakul, N.; Pakawatchai, C.; Saithong, S.; Hansongnern, K. Bis[5-Chloro-2-(Phenyldiazenyl-κ *N*^{2})Pyridine-κ *N*]Bis(Thiocyanato-κ *N*)Iron(II). *Acta Crystallogr. Sect. E Struct. Rep. Online* **2012**, *68* (5), m555–m556. https://doi.org/10.1107/S1600536812014286.

(260) Reger, D. L.; Gardinier, J. R.; BakbakCurrent Address: School Of Ch, S.; Semeniuc, R. F.; Bunz, U. H. F.; Smith, M. D. Multitopic Third Generation Tris(Pyrazolyl)Methane Ligands Built on Alkyne Structural Scaffolding: First Preparation of Mixed Tris(Pyrazolyl)Methane/Tris(Pyrazolyl)Borate Ligands. *New J. Chem.* **2005**, *29* (8), 1035. https://doi.org/10.1039/b414770g.

(261) Janiak, C. Clathrates and Coordination Polymers: New Dimensions in Poly(Azolyl)Borate Chemistry with Poly-(Triazolyl)- and -(Tetrazolyl)-Borate Ligands. *J. Chem. Soc. Chem. Commun.* **1994**, No. 4, 545. https://doi.org/10.1039/c39940000545.

(262) Benniston, A. C.; Farrugia, L. J.; Mackie, P. R.; Mallinson, P.; Clegg, W.; Teat, S. J. Properties and Single-Crystal X-Ray Structure of Bis[3,3″-Bis(4-Methylphenyl)-2,2′:6′,2″-Terpyridine]Iron(II) Hexafluorophosphate - Acetonitrile - Diisopropyl Ether (1/1.5/1). *Aust. J. Chem.* **2000**, *53* (8), 707. https://doi.org/10.1071/CH99167.

(263) Belfrekh, N.; Dietrich-Buchecker, C.; Sauvage, J.-P. Unexpected Synthesis of an 8-Shaped Macrocycle Instead of an Interlocking-Ring System. *Inorg. Chem.* **2000**, *39* (22), 5169–5172. https://doi.org/10.1021/ic991502k.

(264) Constable, E. C.; Housecroft, C. E.; Neuburger, M.; Phillips, D.; Raithby, P. R.; Schofield, E.; Sparr, E.; Tocher, D. A.; Zehnder, M.; Zimmermann, Y. Development of Supramolecular Structure through Alkylation of Pendant Pyridyl Functionality. *J. Chem. Soc. Dalton Trans.* **2000**, No. 13, 2219–2228. https://doi.org/10.1039/b000940g.

(265) Smith, C. B.; Constable, E. C.; Housecroft, C. E.; Kariuki, B. M.

Formation of a [1 + 1] Metallomacrocycle from a Heterotritopic Ligand Containing Two Terpy and One Bipy Metal-Binding Domains. *Chem Commun* **2002**, No. 18, 2068–2069. https://doi.org/10.1039/B205233D.

(266) Hayami, S.; Danjobara, K.; Shigeyoshi, Y.; Inoue, K.; Ogawa, Y.; Maeda, Y. Crystal Structure and Mesogenic Property of an Iron(II) Complex with a Terpyridine Derivative Ligand. *Inorg. Chem. Commun.* **2005**, *8* (6), 506–509. https://doi.org/10.1016/j.inoche.2005.03.002.

(267) Pitarch López, J.; Kraus, W.; Reck, G.; Thünemann, A.; Kurth, D. G. Synthesis, Structure and Reactivity of the Homoleptic Iron(II) Complex of the Novel 4′-(4‴-Pyridyl-N-Oxide)-2,2′:6′,2″-Terpyridine Ligand. *Inorganica Chim. Acta* **2005**, *358* (12), 3384–3390. https://doi.org/10.1016/j.ica.2005.04.034.

(268) Dell'Amico, D. B.; Calderazzo, F.; Englert, U.; Labella, L.; Marchetti, F. The First Crystallographically Established Bis-Qtpy (Qtpy = 2,2′:6′,2″:6″,2‴-Quaterpyridine) Metal Complex. *J. Chem. Soc. Dalton Trans.* **2001**, No. 4, 357–358. https://doi.org/10.1039/b100360g.

(269) Yang, M.; Lin, T.-W.; Chou, C.-C.; Lee, H.-C.; Chang, H.-C.; Lee, G.-H.; Leung, M.; Peng, S.-M. New Oligo-α-Pyridylamino Ligands and Their Metal Complexes. *Chem. Commun.* **1997**, No. 23, 2279–2280. https://doi.org/10.1039/A706439J.

(270) Scudder, M. L.; Craig, D. C.; Goodwin, H. A. Hydrogen Bonding Influences on the Properties of Heavily Hydrated Chloride Salts of Iron(Ii) and Ruthenium(Ii) Complexes of 2,6-Bis(Pyrazol-3-Yl)Pyridine, 2,6-Bis(1,2,4-Triazol-3-Yl)Pyridine and 2,2′:6′,2″-Terpyridine. *CrystEngComm* **2005**, *7* (107), 642. https://doi.org/10.1039/b511825e.

(271) Sugiyarto, K.; Craig, D.; Rae, A.; Goodwin, H. Structural, Magnetic and Mössbauer Spectral Studies of Salts of Bis[2,6-Bis(Pyrazol-3-Yl)Pyridine]Iron(II)—a Spin Crossover System. *Aust. J. Chem.* **1994**, *47* (5), 869. https://doi.org/10.1071/CH9940869.

(272) Sugiyarto, K. H.; Weitzner, K.; Craig, D. C.; Goodwin, H. A. Structural, Magnetic and MöSsbauer Studies of Bis(2,6-Bis(Pyrazol-3-Yl)Pyridine)Iron(II) Triflate and Its Hydrates. *Aust. J. Chem.* **1997**, *50* (9), 869. https://doi.org/10.1071/C96206.

(273) Clemente-León, M.; Coronado, E.; Giménez-López, M. C.; Romero, F. M.; Asthana, S.; Desplanches, C.; Létard, J.-F. Structural, Thermal and Photomagnetic Properties of Spin Crossover [Fe(Bpp)2]2+ Salts Bearing [Cr(L)(Ox)2]− Anions. *Dalton Trans.* **2009**, No. 38, 8087.

https://doi.org/10.1039/b902825k.

(274) Jornet-Mollá, V.; Duan, Y.; Giménez-Saiz, C.; Waerenborgh, J. C.; Romero, F. M. Hydrogen-Bonded Networks of $[Fe(Bpp)_2]^{2+}$ Spin Crossover Complexes and Dicarboxylate Anions: Structural and Photomagnetic Properties. *Dalton Trans* **2016**, *45* (44), 17918–17928. https://doi.org/10.1039/C6DT02934E.

(275) Jornet-Mollá, V.; Giménez-Saiz, C.; Romero, F. M. Synthesis, Structure, and Photomagnetic Properties of a Hydrogen-Bonded Lattice of [Fe(Bpp)2]2+ Spin-Crossover Complexes and Nicotinate Anions. *Crystals* **2018**, *8* (11), 439. https://doi.org/10.3390/cryst8110439.

(276) Childs, B.; Craig, D.; Ross, K.; Scudder, M.; Goodwin, H. Structural and Electronic Properties of Bis[2-(Pyrazin-2-Ylamino)-4-(Pyridin-2-Yl)Thiazolato]Iron(II) and Its Solvated Derivatives. *Aust. J. Chem.* **1994**, *47* (5), 891. https://doi.org/10.1071/CH9940891.

(277) Dumitru, F.; Legrand, Y.-M.; Van Der Lee, A.; Barboiu, M. Constitutional Self-Sorting of Homochiral Supramolecular Helical Single Crystals from Achiral Components. *Chem. Commun.* **2009**, No. 19, 2667. https://doi.org/10.1039/b822619a.

(278) Scheer, C.; Chautemps, P.; Gautier-Luneau, I.; Pierre, J.-L.; Serratrice, G.; Saint-Aman, E. Interlocked Ligands in an Octahedral Complex of the Fe11L2 Type, Using Tridentate L Ligands. *Polyhedron* **1996**, *15* (2), 219–224. https://doi.org/10.1016/0277-5387(95)00274-V.

(279) Bonhôte, P.; Ferigo, M.; Stoeckli-Evans, H.; Marty, W. Structures of 2,6-Bis(Aminomethyl)Pyridine (Bamp) Complexes of FeII, NiII, ZnII, MnII, CoIII and CuII. *Acta Crystallogr. C* **1993**, *49* (12), 2102–2107. https://doi.org/10.1107/S010827019300424X.

(280) Butcher, R. J.; Addison, A. W. Structural Aspects of the Bis(2,2′-Dipicolylamine)Iron(II) Cation. *Inorganica Chim. Acta* **1989**, *158* (2), 211–215. https://doi.org/10.1016/S0020-1693(00)80835-X.

(281) Wocadlo, S.; Massa, W.; Folgado, J.-V. Synthesis and Structural, Spectroscopic and Magnetic Characterization of Iron(II) and Iron(III) Complexes of N-2-Pyridinylcarbonyl-2-Pyridinecarboximidate. *Inorganica Chim. Acta* **1993**, *207* (2), 199–206. https://doi.org/10.1016/S0020-1693(00)90710-2.

(282) Ierno, H.; Jordanov, J.; Laugier, J.; Greneche, J.-M. *New J. Chem.* **1997**, *21*, 241.

(283) Boeyens, J. C. A.; Forbes, A.; Hancock, R. D.; Wieghardt, K. Crystallographic Study of the Low-Spin Iron(II) and Iron(III) Bis Complexes of

1,4,7-Triazacyclononane. *Inorg. Chem.* **1985**, *24* (19), 2926–2931. https://doi.org/10.1021/ic00213a011.

(284) Rüttimann, S.; Moreau, C. M.; Williams, A. F.; Bernardinelli, G.; Addison, A. W. Complexes of Structural Analogues of Terpyridyl with Iron and Zinc; the x-Ray Crystal Structure of Bis[2,6-Bis(Benzimidazol2-Yl)Pyridine]Iron(II) Trifluoromethylsulphonate Bis-Ethanol Solvate. *Polyhedron* **1992**, *11* (6), 635–646. https://doi.org/10.1016/S0277-5387(00)83320-3.

(285) Petzold, H.; Hörner, G.; Schnaubelt, L.; Rüffer, T. Slow Spin Crossover in Bis-Meridional Fe^{2+} Complexes through Spin-State Auto-Adaptive N6/N8 Coordination. *Dalton Trans.* **2018**, *47* (48), 17257–17265. https://doi.org/10.1039/C8DT03652G.

(286) Costa, J. S.; Rodríguez-Jiménez, S.; Craig, G. A.; Barth, B.; Beavers, C. M.; Teat, S. J.; Aromí, G. Three-Way Crystal-to-Crystal Reversible Transformation and Controlled Spin Switching by a Nonporous Molecular Material. *J. Am. Chem. Soc.* **2014**, *136* (10), 3869–3874. https://doi.org/10.1021/ja411595y.

(287) Ishida, A. K. *CSD Commun. Priv. Commun.* **2017**. https://doi.org/CCDC%25201559027.

(288) Vasilevsky, I.; Stenkamp, R. E.; Lingafelter, E. C.; Rose, N. J. Syntheses and Structures of μ-Oxo-Bis[Dichloroiron(III)]-Bis-[2,6-Diacetylpyridinedioximate(-1)]Iron(II) and Related Compounds. *J. Coord. Chem.* **1988**, *19* (1–3), 171–187. https://doi.org/10.1080/00958972.1988.9728153.

(289) Wang, K.; Hao, P.; Zhang, D.; Sun, W.-H. Tridentate N^N^N Iron(II) and Cobalt(II) Complexes of Ion-Paired Structures: Synthesis, Characterization and Magnetism. *J. Mol. Struct.* **2008**, *890* (1–3), 95–100. https://doi.org/10.1016/j.molstruc.2008.03.027.

(290) Bao, F.-F.; Xu, X.-X.; Zhou, W.; Pang, C.-Y.; Xi, S.-F.; Gu, Z.-G.; Li, Z.-J. Synthesis, Structures and DNA-Binding of Enantiomers of Fe(II) and Ni(II) Schiff Base Complexes. *Chin. J. Inorg. Chem.* **2014**, *30* (8), 1748–1756. https://doi.org/10.11862/CJIC.2014.252.

(291) Ionkin, A. S.; Marshall, W. J.; Adelman, D. J.; Shoe, A. L.; Spence, R. E.; Xie, T. Nitro-substituted Iron(II) Tridentate Bis(Imino)Pyridine Complexes as High-temperature Catalysts for the Production of A-olefins. *J. Polym. Sci. Part Polym. Chem.* **2006**, *44* (8), 2615–2635. https://doi.org/10.1002/pola.21360.

(292) Duarte, G. M.; Braun, J. D.; Giesbrecht, P. K.; Herbert, D. E. Redox Non-Innocent Bis(2,6-Diimine-Pyridine) Ligand–Iron Complexes as Anolytes for

Flow Battery Applications. *Dalton Trans.* **2017**, *46* (47), 16439–16445. https://doi.org/10.1039/C7DT03915H.

(293) Diebold, A.; Hagen, K. S. Iron(II) Polyamine Chemistry: Variation of Spin State and Coordination Number in Solid State and Solution with Iron(II) Tris(2-Pyridylmethyl)Amine Complexes. *Inorg. Chem.* **1998**, *37* (2), 215–223. https://doi.org/10.1021/ic971105e.

(294) Chen, K.; Que, L. Stereospecific Alkane Hydroxylation by Non-Heme Iron Catalysts: Mechanistic Evidence for an Fe^{V} O Active Species. *J. Am. Chem. Soc.* **2001**, *123* (26), 6327–6337. https://doi.org/10.1021/ja010310x.

(295) Lange, S. J.; Miyake, H.; Que, L. Evidence for a Nonheme Fe(IV)O Species in the Intramolecular Hydroxylation of a Phenyl Moiety. *J. Am. Chem. Soc.* **1999**, *121* (26), 6330–6331. https://doi.org/10.1021/ja990233u.

(296) Zang, Y.; Kim, J.; Dong, Y.; Wilkinson, E. C.; Appelman, E. H.; Que, L. Models for Nonheme Iron Intermediates: Structural Basis for Tuning the Spin States of Fe(TPA) Complexes. *J. Am. Chem. Soc.* **1997**, *119* (18), 4197–4205. https://doi.org/10.1021/ja9638521.

(297) Börzel, H.; Comba, P.; Hagen, K. S.; Lampeka, Y. D.; Lienke, A.; Linti, G.; Merz, M.; Pritzkow, H.; Tsymbal, L. V. Iron Coordination Chemistry with Tetra-, Penta- and Hexadentate Bispidine-Type Ligands. *Inorganica Chim. Acta* **2002**, *337*, 407–419. https://doi.org/10.1016/S0020-1693(02)01100-3.

(298) Bréfuel, N.; Lepetit, C.; Shova, S.; Dahan, F.; Tuchagues, J.-P. Complexation to Fe^{II} , Ni^{II} , and Zn^{II} of Multidentate Ligands Resulting from Condensation of 2-Pyridinecarboxaldehyde with α,ω-Triamines: Selective Imidazolidine/Hexahydropyrimidine Ring Opening Revisited. *Inorg. Chem.* **2005**, *44* (24), 8916–8928. https://doi.org/10.1021/ic050791b.

(299) Kurosaki, H.; Ishikawa, Y.; Ishihara, T.; Yamamoto, T.; Yamaguchi, Y.; Goto, M. Mechanism of Formation of Iron(II) Complexes with Pentadentate Ligands via C–C Bond Formation between Trans-[Fe(2,4-Bis(2-Pyridylmethylimino)Pentane)$(MeCN)_2$]$[ClO_4]_2$ ·MeCN and Various Nitriles. *Dalton Trans* **2005**, No. 6, 1086–1092. https://doi.org/10.1039/B414207A.

(300) Goto, M.; Ishikawa, Y.; Ishihara, T.; Nakatake, C.; Higuchi, T.; Kurosaki, H.; Goedken, T. L. V. L. Synthesis of an Iron(Ii) Complex Versatile for Preparation of Iron(Ii) Complexes with Novel Pentadentate Ligands via C–C Bond Formation with Various Nitriles: 1,3-Dimethyl-1,3-Propanediylidenebis(2-Pyridylmethanamine)Iron(Ii) Perchlorate. *Chem. Commun.* **1997**, No. 6, 539–540. https://doi.org/10.1039/a700283a.

(301) Ye, Y. S.; Chen, X. Q.; De Cai, Y.; Fei, B.; Dechambenoit, P.; Rouzières, M.; Mathonière, C.; Clérac, R.; Bao, X. Slow Dynamics of the Spin-Crossover Process in an Apparent High-Spin Mononuclear Fe^{II} Complex. *Angew. Chem. Int. Ed.* **2019**, *58* (52), 18888–18891. https://doi.org/10.1002/anie.201911538.

(302) Roelfes, G.; Lubben, M.; Chen, K.; Ho, R. Y. N.; Meetsma, A.; Genseberger, S.; Hermant, R. M.; Hage, R.; Mandal, S. K.; Young, V. G.; Zang, Y.; Kooijman, H.; Spek, A. L.; Que, L.; Feringa, B. L. Iron Chemistry of a Pentadentate Ligand That Generates a Metastable Fe^{III} −OOH Intermediate. *Inorg. Chem.* **1999**, *38* (8), 1929–1936. https://doi.org/10.1021/ic980983p.

(303) Van Den Heuvel, M.; Van Den Berg, T. A.; Kellogg, R. M.; Choma, C. T.; Feringa, B. L. Synthesis of a Non-Heme Template for Attaching Four Peptides: An Approach to Artificial Iron(II)-Containing Peroxidases. *J. Org. Chem.* **2004**, *69* (2), 250–262. https://doi.org/10.1021/jo035157z.

(304) De Vries, M. E.; La Crois, R. M.; Roelfes, G.; Kooijman, H.; Spek, A. L.; Hage, R.; Feringa, B. L. A Novel Pentadentate Ligand 2,6-Bis[Methoxybis(2-Pyridyl)Methyl]Pyridine L for Mononuclear Iron(Ii) and Manganese(Ii) Compounds; Synthesis and Crystal Structures of [FeL(MeCN)][ClO4]2 and [(MnL(H2O)][ClO4]2. *Chem. Commun.* **1997**, No. 16, 1549–1550. https://doi.org/10.1039/a702804k.

(305) Goldsmith, C. R.; Jonas, R. T.; Cole, A. P.; Stack, T. D. P. A Spectrochemical Walk: Single-Site Perturbation within a Series of Six-Coordinate Ferrous Complexes. *Inorg. Chem.* **2002**, *41* (18), 4642–4652. https://doi.org/10.1021/ic025616z.

(306) Patra, A. K.; Afshar, R.; Olmstead, M. M.; Mascharak, P. K. The First Non-Heme Iron(III) Complex with a Ligated Carboxamido Group That Exhibits Photolability of a Bound NO Ligand. *Angew. Chem. Int. Ed.* **2002**, *41* (14), 2512–2515. https://doi.org/10.1002/1521-3773(20020715)41:14%253C2512::AID-ANIE2512%253E3.0.CO;2-7.

(307) Goto, M.; Ishikawa, Y.; Ishihara, T.; Nakatake, C.; Higuchi, T.; Kurosaki, H.; Goedken, V. L. Iron(II) Complexes with Novel Pentadentate Ligands via C–C Bond Formation between Various Nitriles and [2,4-Bis(2-Pyridylmethylimino)Pentane]Iron(II) Perchlorate: Synthesis and Structures ‡. *J. Chem. Soc. Dalton Trans.* **1998**, No. 7, 1213–1222. https://doi.org/10.1039/a708589c.

(308) Collinson, S.; Alcock, N. W.; Raghunathan, A.; Kahol, P. K.; Busch,

D. H. Synthesis and Properties of Iron(II) and Manganese(II) Complexes Derived from a Topologically Constrained Pentadentate Ligand. *Inorg. Chem.* **2000**, *39* (4), 757–764. https://doi.org/10.1021/ic981410f.

(309) Roelfes, G.; Vrajmasu, V.; Chen, K.; Ho, R. Y. N.; Rohde, J.-U.; Zondervan, C.; La Crois, R. M.; Schudde, E. P.; Lutz, M.; Spek, A. L.; Hage, R.; Feringa, B. L.; Münck, E.; Que, L. End-On and Side-On Peroxo Derivatives of Non-Heme Iron Complexes with Pentadentate Ligands: Models for Putative Intermediates in Biological Iron/Dioxygen Chemistry. *Inorg. Chem.* **2003**, *42* (8), 2639–2653. https://doi.org/10.1021/ic034065p.

(310) Ugalde-Saldívar, V. M.; Sosa-Torres, M. E.; Ortiz-Frade, L.; Bernès, S.; Höpfl, H. Novel Iron(Ii) Complexes with Hexadentate Nitrogen Ligands Obtained via Intramolecular Redox Reactions. *J. Chem. Soc. Dalton Trans.* **2001**, No. 20, 3099. https://doi.org/10.1039/b100915j.

(311) Ugalde-Saldívar, V. M.; Höpfl, H.; Farfán, N.; Toscano, A. R.; Sosa-Torres, M. E. Comparative Study of the Influence of the Metal Centres: Fe(III), Cu(II) and Zn(II), on the Ring Opening and Oxidative Dehydrogenation Reactions Occurring in a Coordinated Imidazolidine Ligand. *Inorganica Chim. Acta* **2005**, *358* (13), 3545–3558. https://doi.org/10.1016/j.ica.2005.03.052.

(312) Di Vaira, M.; Mani, F.; Stoppioni, P. Synthesis and Crystal Structure of the Iron(II)–Iron(III) Complex [FeL][FeCl4]Cl [L = 1,4,7-Tris(Pyrazol-3-Ylmethyl)-1,4,7-Triazacyclononane]. *J. Chem. Soc. Dalton Trans.* **1997**, No. 8, 1375–1380. https://doi.org/10.1039/a608138j.

(313) Alcock, N. W.; Zhang, D.; Busch, D. H. Tris(2-Pyridylmethyl)Triazacyclododecane Complexes of Fe^{II} and Cu^{II}. *Acta Crystallogr. C* **1999**, *55* (6), 886–889. https://doi.org/10.1107/S0108270198018381.

(314) Lambert, F.; Policar, C.; Durot, S.; Cesario, M.; Yuwei, L.; Korri-Youssoufi, H.; Keita, B.; Nadjo, L. Imidazole and Imidazolate Iron Complexes: On the Way for Tuning 3D-Structural Characteristics and Reactivity. Redox Interconversions Controlled by Protonation State. *Inorg. Chem.* **2004**, *43* (14), 4178–4188. https://doi.org/10.1021/ic0498687.

(315) Sunatsuki, Y.; Ohta, H.; Kojima, M.; Ikuta, Y.; Goto, Y.; Matsumoto, N.; Iijima, S.; Akashi, H.; Kaizaki, S.; Dahan, F.; Tuchagues, J.-P. Supramolecular Spin-Crossover Iron Complexes Based on Imidazole−Imidazolate Hydrogen Bonds. *Inorg. Chem.* **2004**, *43* (14), 4154–4171. https://doi.org/10.1021/ic0498384.

(316) Xu, L.-C.; Zhang, S.-Q.; Li, X.; Tang, M.-J.; Xie, P.-P.; Hong, X.

Towards Data-Driven Design of Asymmetric Hydrogenation of Olefins: Database and Hierarchical Learning. *Angew. Chem. Int. Ed.* **2021**, *60* (42), 22804–22811. https://doi.org/10.1002/anie.202106880.
